# Genomes reveal pervasive distant hybridization in nature among cyprinid fishes

**DOI:** 10.1093/gigascience/giae117

**Published:** 2025-01-30

**Authors:** Li Ren, Xiaolong Tu, Mengxue Luo, Qizhi Liu, Jialin Cui, Xin Gao, Hong Zhang, Yakui Tai, Yiyan Zeng, Mengdan Li, Chang Wu, Wuhui Li, Jing Wang, Dongdong Wu, Shaojun Liu

**Affiliations:** State Key Laboratory of Developmental Biology of Freshwater Fish, Engineering Research Center of Polyploid Fish Reproduction and Breeding of the State Education Ministry, College of Life Sciences, Hunan Normal University, Changsha 410081, China; State Key Laboratory of Genetic Resources and Evolution, Kunming Institute of Zoology, Chinese Academy of Sciences, Kunming 650201, China; Kunming College of Life Science, University of the Chinese Academy of Sciences, Kunming 650204, China; State Key Laboratory of Developmental Biology of Freshwater Fish, Engineering Research Center of Polyploid Fish Reproduction and Breeding of the State Education Ministry, College of Life Sciences, Hunan Normal University, Changsha 410081, China; State Key Laboratory of Developmental Biology of Freshwater Fish, Engineering Research Center of Polyploid Fish Reproduction and Breeding of the State Education Ministry, College of Life Sciences, Hunan Normal University, Changsha 410081, China; State Key Laboratory of Developmental Biology of Freshwater Fish, Engineering Research Center of Polyploid Fish Reproduction and Breeding of the State Education Ministry, College of Life Sciences, Hunan Normal University, Changsha 410081, China; State Key Laboratory of Developmental Biology of Freshwater Fish, Engineering Research Center of Polyploid Fish Reproduction and Breeding of the State Education Ministry, College of Life Sciences, Hunan Normal University, Changsha 410081, China; State Key Laboratory of Developmental Biology of Freshwater Fish, Engineering Research Center of Polyploid Fish Reproduction and Breeding of the State Education Ministry, College of Life Sciences, Hunan Normal University, Changsha 410081, China; State Key Laboratory of Developmental Biology of Freshwater Fish, Engineering Research Center of Polyploid Fish Reproduction and Breeding of the State Education Ministry, College of Life Sciences, Hunan Normal University, Changsha 410081, China; State Key Laboratory of Developmental Biology of Freshwater Fish, Engineering Research Center of Polyploid Fish Reproduction and Breeding of the State Education Ministry, College of Life Sciences, Hunan Normal University, Changsha 410081, China; State Key Laboratory of Developmental Biology of Freshwater Fish, Engineering Research Center of Polyploid Fish Reproduction and Breeding of the State Education Ministry, College of Life Sciences, Hunan Normal University, Changsha 410081, China; State Key Laboratory of Developmental Biology of Freshwater Fish, Engineering Research Center of Polyploid Fish Reproduction and Breeding of the State Education Ministry, College of Life Sciences, Hunan Normal University, Changsha 410081, China; State Key Laboratory of Developmental Biology of Freshwater Fish, Engineering Research Center of Polyploid Fish Reproduction and Breeding of the State Education Ministry, College of Life Sciences, Hunan Normal University, Changsha 410081, China; State Key Laboratory of Developmental Biology of Freshwater Fish, Engineering Research Center of Polyploid Fish Reproduction and Breeding of the State Education Ministry, College of Life Sciences, Hunan Normal University, Changsha 410081, China; State Key Laboratory of Genetic Resources and Evolution, Kunming Institute of Zoology, Chinese Academy of Sciences, Kunming 650201, China; Kunming Natural History Museum of Zoology, Kunming Institute of Zoology, Chinese Academy of Sciences, Kunming 650223, China; State Key Laboratory of Developmental Biology of Freshwater Fish, Engineering Research Center of Polyploid Fish Reproduction and Breeding of the State Education Ministry, College of Life Sciences, Hunan Normal University, Changsha 410081, China

**Keywords:** diet divergence, genetic introgression, phylogenomics, incomplete reproductive isolation

## Abstract

**Background:**

Genomic data have unveiled a fascinating aspect of the evolutionary past, showing that the mingling of different species through hybridization has left its mark on the histories of numerous life forms. However, the relationship between hybridization events and the origins of cyprinid fishes remains unclear.

**Results:**

In this study, we generated *de novo* assembled genomes of 8 cyprinid fishes and conducted phylogenetic analyses on 24 species. Widespread allele sharing across species boundaries was observed within 7 subfamilies of cyprinid fishes. Based on a systematic analysis of multiple tissues, we found that the testis exhibited a conserved pattern of divergence between the herbivorous *Megalobrama amblycephala* and the carnivorous *Culter alburnus*, suggesting a potential link to incomplete reproductive isolation. Significant differences in the expression of 4 genes (*dpp2, ctrl, psb7*, and *ppce*) in the liver and intestine, accompanied by variations in enzyme activities, indicated swift divergence in digestive enzyme secretion. Moreover, we identified introgressed genes linked to organ development in sympatric fishes with analogous feeding habits within the *Cultrinae* and *Leuciscinae* subfamilies.

**Conclusions:**

Our findings highlight the significant role played by incomplete reproductive isolation and frequent gene flow events, particularly those associated with the development of digestive organs, in driving speciation among cyprinid fishes in diverse freshwater ecosystems.

## Introduction

Cyprinidae (order Cypriniformes) is the largest and most diverse family of ray-finned fish, comprising about 13 subfamilies and 370 genera [1, 2]. This family includes popular aquarium fishes like goldfish and koi, as well as the valuable vertebrate model organism, the zebrafish [2]. As a family of freshwater fish, the origin time of Cyprinidae was estimated at 154 million years ago (MYA) [3], and they are now widely distributed in almost all types of water around the world [2]. Their great diversities in feeding and reproductive behaviors, as well as morphology, including body length (ranging from about 8 mm for *Paedocypris progenetica* to approximately 3 m for *Catlocarpio siamensis*) [4, 5] and digestive organs [6, 7], are intriguing to evolutionary biologists due to their phylogenetic relationships and adaptive radiation evolution. However, the narrow distribution ranges or small population sizes of Cyprinidae fish now face threats from human activity, such as overfishing, damming of upland rivers, pollution, habitat destruction, and novel viral infections [8].

Introgression plays a significant and frequent role in adaptive evolution [9]. At least 10% of animal species are involved in hybridization, although most hybrid individuals have low viability or are sterile [10]. While reproductive isolation (RI) is frequently observed in intergeneric hybridization among birds and mammals, it is less common in cyprinid fish species, as evidenced by the documented instances of disrupted isolation [11–13]. Natural selection, including variable water environments and deleterious homozygosity for small population sizes, brings great pressures to the survival of freshwater fish [14, 15]. Hybridization has the potential to generate genetic diversity and create opportunities for novel adaptive radiations, although it has been considered a breakdown of isolating mechanisms [16]. The low viability or sterility of hybrids could reinforce RI through selection for assortative mating and result in adaptive introgression [10]. The rate of introgression depends on the pressures of the freshwater environment and affects fish biodiversity [17]. Natural hybridization involving intergeneric hybridization was always observed among cyprinid fishes [18–20], while bisexual fertile progenies were detected in the laboratory experiments of various hybrid groups [13]. This evidence suggests that prezygotic isolation evolves more rapidly than postzygotic isolation in cyprinid fishes, challenging the assumption of the criticality of variation in dietary niche breadth for speciation. Now, the relationship between biodiversity and introgressive hybridization in cyprinid fishes is still obscure. Phylogenomics from whole genome sequences could provide us with more detailed evidence of it than the fragmented detective technologies, including rDNA [20], mitochondrial DNA, and microsatellites [21].

Diet plays a crucial role in the biodiversity and habitat distribution of fishes and is influenced by differences in foraging behavior and digestive organ morphology [22]. The selection of diet is particularly important for sympatric species [23]. In the case of cyprinid fishes, 4 main categories of diet have been identified. These include herbivorous fishes (e.g., *Megalobrama amblycephala* and *Ctenopharyngodon idella*), carnivorous fishes (e.g., *Culter alburnus* and *Elopichthys bambusa*), filter-feeding fishes (e.g., *Hypophthalmichthys nobilis*), and omnivorous fishes (e.g., *Cyprinus carpio* and *Carassius auratus*) [24]. These fish species are distributed across different water layers to acquire various types of food resources. Cyprinid fishes have evolved unique adaptations for food digestion, such as advanced protrusible pharyngeal teeth, despite the absence of jaw teeth and stomachs [25]. The number and shape of teeth in cyprinid fishes exhibit significant variation and are used as phenotypic features for species classification [2]. In East African cichlids, the number of tooth rows on both jaws has been associated with specific feeding ecologies [26]. However, it remains unclear whether variations in the width of the dietary niche are critical for the biodiversity of cyprinid fishes.

In this study, we obtained the *de novo* assembled genome sequences of 8 cyprinid fish species and conducted comparative genome analyses using a set of 24 high-quality assembled genome sequences. Through gene flow analyses, we investigated hybridization events and their contributions to the speciation of cyprinid fishes. Furthermore, we conducted evolutionary constraints analyses on various tissues and organs and investigated their divergence between *M. amblycephala* and *C. alburnus*. Our findings emphasize the significance of gene flow events in the origin of cyprinid fishes and the functional divergence that drives speciation.

## Methods

### Sample collection

Three sexually mature male *C. alburnus* (NCBI:txid194366) and *M. amblycephala* (NCBI:txid75352), raised in identical controlled conditions for 24 months posthatching, were bred at the Engineering Center of Polyploid Fish Breeding, National Education Ministry, Changsha, Hunan, China. The broodstock were sourced from the Yangtze River (30°25′56″ N, 114°50′32″ E). The parents of *Gobiocypris rarus* (NCBI:txid143606) were obtained from the Liu Sha River, Sichuan Province (coordinates 29°19′31″ N, 102°40′38″ E). We also collected 1 individual of each fish species (*Cirrhinus molitorella* [NCBI:txid172907], *Pseudorasbora parva* [NCBI:txid51549], *Xenocypris davidi* [NCBI:txid291826], *Elopichthys bambusa* [NCBI:txid238031], and *Ctenopharyngodon idella* [NCBI:txid7959]) from Dongting Lake, Hunan, China (coordinates 29°15′8″ N, 112°50′24″ E). These individuals were deeply anesthetized with 300 mg/L tricaine methanesulfonate (Sigma-Aldrich) for 10 minutes (20°C) in a separation tank. After confirming their deaths, the muscle, brain, liver, intestine, kidney, and testis of all samples were collected after dissection.

### DNA isolation and whole genome sequencing

High-quality and high-molecular-weight genomic DNA was isolated from muscle based on the DNA extraction methods. The purification was performed using the QIAGEN Genomic Kit based on the standard operating procedure. The degradation and contamination of the extracted DNA were detected using 1% agarose gels. Then, DNA purity was determined using the NanoDrop One UV-Vis spectrophotometer (Thermo Fisher Scientific) with 260/280 and 260/230 ratios. DNA concentration was measured by the Qubit 4.0 Fluorometer (Invitrogen).

After quality checking, the genomic DNA of *C. alburnus* and *M. amblycephala* was randomly sheared using Megaruptor (Diagenode). DNA was size-selected using an SPRI bead protocol. The purity of the extracted DNA was determined using a NanoDrop spectrophotometer (Thermo Fisher Scientific). All procedures were carried out at room temperature. Large DNA fragments were separated using BluePippin DNA Size Selection System. DNA damage repair and end repair were performed. Barcoded overhang hairpin adapters were ligated to the fragment ends. The connection reaction was performed using the Ligation Sequencing Kit (Oxford Nanopore, SQK-LSK108). A constructed DNA library was quantified using Qubit. Lastly, sequencing was performed using Nanopore sequencing.

The genomic DNA of *C. alburnus* and *M. amblycephala* was utilized for whole genome resequencing. First, high-quality DNA samples were used to prepare single-stranded circular libraries. Subsequently, the circular libraries were transformed into DNA nanoballs (DNBs), which are spherical structures containing millions of copies of the circular DNA templates. Once the DNBs were formed, they were loaded onto patterned nanoarrays. Following the loading of DNBs onto the nanoarrays, combinatorial probe anchor synthesis sequencing was conducted. Finally, DNBSEQ-T7 sequencing was performed using a paired-end approach (150 bp × 2) in accordance with the standard protocol [27].

In total, 15 μg DNA for the 6 fishes (*C. idella, C. molitorella, P. parva, X. davidi, G. rarus*, and *E. bambusa*) was used for the preparation of SMRTbell target-size libraries, which were constructed using PacBio’s standard protocol (Pacific Biosciences) with 15-kb preparation solutions. The main steps for library preparation are listed as follows: (i) The genomic DNA was sheared using g-TUBEs (Covaris), (ii) an A-tailing reaction was used to form an overhang, (iii) the fragments were ligated with the hairpin adapter using the SMRTbell Express Template Prep Kit 2.1 (Pacific Biosciences), (iv) the library was treated with nuclease and purified using AMPure PB Beads, and (v) the SMRTbell library was purified using PB beads. The high-quality library was checked for fragment size using the Agilent 2100 Bioanalyzer (Agilent Technologies). Sequencing Primer V2 and Sequel II Binding Kit 2.1 were used for PacBio Sequel II sequencing.

### Genome assembly and chromosomal organization

The adapter and low-quality bases of the 2 species were filtered before assembly using Fastp (RRID:SCR_016962) (v. 0.21.0) [28]. All clean reads of *C. alburnus* and *M. amblycephala* were used for genome assembly using Nextdenovo (RRID:SCR_025033) (v. 2.3.0) [29]. The parameters “random_round = 20, minimap2_options_cns = -x ava-ont -t 40 -k17 -w17, and nextgraph_options = -a 0” were used in genome assembly. The base errors in the genome generated were fixed using Nextpolish (RRID:SCR_025232) (v. 1.3.0) [30]. For the 6 fishes (*C. idella, C. molitorella, P. parva, X. davidi, G. rarus*, and *E. bambusa*), the HIFI data was used for genome assembly using hifiasm (0.15.4-r347) software (RRID:SCR_021069) [31].

Hi-C libraries of *C. alburnus* and *M. amblycephala* were created from muscle cells. Briefly, cells were fixed with formaldehyde and lysed, and the cross-linked DNA was digested with MobI. Sticky ends were biotinylated and proximity ligated to form chimeric junctions that were enriched for and then physically sheared to a size of 300–700 bp, as illustrated in Rao et al. [32]. Chimeric fragments representing the original cross-linked long-distance physical interactions were then processed into paired-end sequencing libraries. The clean reads of Hi-C were obtained from trimming adapter sequences and low-quality pair-end reads, which were truncated at the putative Hi-C junctions, and then the resulting trimmed reads were aligned to the assembly results with BWA (RRID:SCR_010910) (v. 0.7.17) [33]. Invalid read pairs, including Dangling-End and Self-cycle, Re-ligation, and Dumped products, were filtered by HiC-Pro (RRID:SCR_017643) (v. 2.8.1) [34]. They were used for the correction of scaffolds and the clustering, ordering, and orientation of scaffolds onto chromosomes by LACHESIS (release: 2017-12-21) [35]. After this step, placement and orientation errors exhibiting obvious discrete chromatin interaction patterns were manually adjusted.

### Gene prediction and annotation

For protein-coding gene prediction in the genomes of *C. alburnus* and *M. amblycephala*, we employed 3 integrated methods: *de novo* prediction, homology search, and cDNA-based prediction (muscle, brain, liver, intestine, kidney, and testis). *De novo* gene models were predicted using Augustus (RRID:SCR_008417) (v. 3.4.0) [36] with default parameters. In the homolog-based analysis, protein genes from 5 species (*C. carpio*: GCF_018340385.1, *C. auratus*: GCF_003368295.1, *O. macrolepis*: GCA_012432095.1, *P. tetrazona*: GCF_018831695.1, and *D. rerio*: GCF_000002035.6) obtained from NCBI were used to predict gene regions using GeneWise (v. 2.4.1) with default parameters. The cDNA-based approaches involved using Hisat2 (RRID:SCR_015530) (v. 2.1.0) [37] and TransDecoder (RRID:SCR_017647) (v. 5.5.0) software to predict open reading frames (ORFs). Subsequently, we integrated the results of genome annotation using GETA (v. 2.5.7). Gene functional predictions were assigned using Blast-2.11.0+ against public databases, including Swiss-Prot, the Non-Redundant Protein Sequence Database (NR), and the EuKaryotic Orthologous Groups database; KEGG Orthology annotations were conducted with Kofamscan software; and motifs and domains were predicted using Hmmer [38] software against the PFAM database.

To identify repetitive sequences, we utilized both *de novo–*based and homology-based methods. First, LTR_FINDER_parallel (RRID:SCR_018969) (v. 1.1) [39], LTRharvest (GenomeTools, v. 1.6.1) [40], LTR_retriever (RRID:SCR_017623) (v. 2.9.0) [41], and RepeatModeler (RRID:SCR_015027) (v. 2.0.1) software were employed to build a *de novo* repeat library, which was then merged with the Repbase database. RepeatMasker (RRID:SCR_012954) was subsequently used to predict repeat sequences using the new repeat library database. Tandem repeats were detected using Tandem Repeats Finder (TRF). For transfer RNA (tRNA) identification, we used tRNAscan-SE (RRID:SCR_008637) (v 2.0.7) [42], while ribosomal RBA (rRNA) was annotated using Blastn (RRID:SCR_001598) (BLAST v. 2.2.26, e-value: 1e^−5^) against the human rRNA sequence from the Rfam database. The small nuclear RNA (snRNA) and microRNA (miRNA) were searched using the Rfam database and the Infernal (RRID:SCR_011809) (v. 1.0.2) software.

### Comparative phylogenomics

For the phylogeny analyses, we performed multiple whole genome alignments (WGAs) for 17 (nonpolyploid species) and 24 (including 7 polyploid species) species using cactus (v. 2.1.1), respectively [43]. The WGAs were utilized to construct a phylogenetic tree with *Beaufortia kweichowensis* as the root. To facilitate the analysis, syntenic blocks were concatenated into 10-kb windows. Subsequently, a file containing 6 Mb (17 species) and 4.75 Mb (24 species) sequences for each genome was generated, respectively. To build the maximum likelihood tree, we employed RAxML (RRID:SCR_006086) (v. 8.2.12) [44] with the following parameters: -p 12,345 -# 100 -m GTRGAMMA -s all.phylip -o B.kweichowensis -f a -x 12,345 -k -n tree -T 10. The coalescent species tree estimations were performed using Astral (RRID:SCR_001886) (v. 5.15.5) [45]. For estimating divergence times, we used the MCMCTree (RRID:SCR_025348) in PAML (4.9j) [46] with 4 fossil calibration time points. The conserved scores of the 17 nonpolyploid species were estimated using the phastCons tool from the phast packages [47]. VCF files for each species were generated by aligning whole genomes to the zebrafish genome using Chen’s methods [48]. To investigate gene flow, we exclusively analyzed the 17 nonpolyploid genomes from non-inbred populations using the ABBA-BABA test implemented in the Dsuite (0.4 r38) software [49] with the D-statistic method. The results were visualized using the Fbranch and dtools.py programs in Dsuite. To ensure a sufficient number of informative sites for analysis within each examined window, we employed a Python script named “ABBABABAwindows.py” to detect window D values. We used a window size of 20 kb with a step size of 10 kb, implemented through the script’s parameters “-w 20,000 -m 100 -s 10,000.”

### RNA isolation and messenger RNA sequencing

Total RNA from the brain, liver, intestine, muscle, kidney, and testis organs of 3 individuals (*C. alburnus* and *M. amblycephala*) was isolated and purified according to the TRIzol extraction method, respectively [50]. The RNA concentration was measured using NanoDrop technology. Total RNA samples were treated with DNase I (Invitrogen) to remove any contaminating genomic DNA. The purified RNA was quantified using a 2100 Bioanalyzer system (Agilent). The isolated messenger RNA (mRNA) was fragmented with a fragmentation buffer. The resulting short fragments were reverse transcribed and amplified to produce cDNA. The transcriptome data of 36 samples (3 biological replicates) were obtained using DNA nanoball DNBSEQ-T7 (RRID:SCR_017981) technology according to the standard method [51]. The main steps are listed as follows: (i) single-stranded circular libraries were prepared using MGI Library Prep Kits; (ii) after the hybridization of a DNA anchor, a fluorescent probe is attached to the DNA nanoball using combinatorial probe anchor sequencing chemistry; (iii) the high-resolution imaging system captures the fluorescent signal; and (iv) after digital processing of the optical signal, the sequencer generates high-quality and accurate sequencing information. Low-quality bases and adapters were trimmed out using SOAPnuke with the thresholds “-n 0.01 -l 20 -q 0.4 -A 0.25 –cutAdaptor -Q 2 -G –polyX 50 –minLen 150” [52]. The high-quality reads were used in the next analyses.

### Gene expression profiling

All clean reads of *M. amblycephala* and *C. alburnus* were mapped to their corresponding reference genomes using HISAT2 (v. 2.1.0) [37] with default parameters. Then, the mapped files were handled with SAMtools/BCFtools (RRID:SCR_005227) (v. 1.10) [53], while the unique mapped reads were obtained using htseq-count (RRID:SCR_011867) (v. 0.12.4) [54]. The gene expression value of mRNA sequencing (mRNA-seq) was normalized and calculated based on the transcripts per million (TPM) values. Genes with mapped reads <5 in each sample were not used in our next analyses. Differential expression (DE) analysis was performed using Deseq2 (RRID:SCR_015687) [55] of the R package with the following thresholds: *P* < 0.001 and *P*adj < 0.001. Organ-specific genes (OSGs) were identified using the following criteria: gene expression in the target tissue or organ differs significantly from that in the other 5 tissues and organs. Orphan genes (OGs) were detected based on the thresholds of BLASTx with an e-value of 1e^−5^ and tBLASTx with an e-value of 1e^−5^. The sequences with no BLAST result in the public database were considered potential OGs. Then, the expressed OGs (TPM >10) were considered OGs in the corresponding tissue or organ. GO analysis was performed with a significance threshold (false discovery rate of the Benjamini–Hochberg method <0.05).

### Diversifying selection analysis

In total, 17,337 orthologous gene pairs (OGPs) between *M. amblycephala* and *C. alburnus* were obtained using the all-against-all reciprocal BLASTP (v. 2.8.1) with an e-value of 1e^−6^ based on protein sequences (sequence alignment >70%). Then, transcripts that were shorter than 300 bp were discarded from OGPs. OGPs in the comparison of *M. amblycephala* and zebrafish and the comparison of *M. amblycephala* and zebrafish were obtained based on the above thresholds. We performed DE analyses on the OGPs between *M. amblycephala* and *C. alburnus* in the 6 tissues and organs. DE analysis was performed using Deseq2 [55] with the following thresholds: *P* < 0.001 and *P*adj < 0.001. The Ks and Ka/Ks values were calculated based on the following analysis process: (i) ParaAT2.0 and muscle software were used in sequence alignment of OGPs with the default parameters, and (ii) kaks_calculator3.0 program was used to calculate Ks and Ka/Ks values using the maximum likelihood method [56]. The threshold of *P* < 0.05 was used in our analyses.

### Measurement of enzymatic content

Equal amounts of liver (0.1 g) from *M. amblycephala* and *C. alburnus* (10 individuals in each species) were collected from the Engineering Center of Polyploid Fish Breeding of National Education Ministry in Hunan, China. Then, homogenates were used to determine the activity of trypsin and lipase. A trypsin assay kit (A080-2-2) and a lipase assay kit (A054-1-1) were purchased from Nanjing Jiancheng Bioengineering Institute (Jiangsu, China), and the experimental protocols followed the manufacturer’s instructions. The significant difference was performed using Student’s *t*-test.

### Hematoxylin and eosin staining

A 10-mm-thick section of skeletal muscle from 4 fish species, *M. amblycephala, C. alburnus, C. idella*, and *E. bambusa*, was dissected from the dorsum region. A 10-mm-thick section of intestine was dissected from the abdominal cavity of each of the 4 fish species after removing food residues. The tissue sections were then fixed in Bouin’s solution for 24 hours. After fixation, the tissues were washed with distilled water for 4 hours at room temperature. The fixed tissues were then dehydrated using a series of alcohol concentrations (e.g., 70%, 80%, 90%, and 100%) and embedded in paraffin blocks. The paraffin-embedded tissue blocks were sectioned into 10-μm-thick slices using a microtome. The tissue sections were processed for hematoxylin and eosin (HE) staining according to the manufacturer’s instructions using an HE staining kit. Digital images of the stained sections were captured using a microscope (DX8; Olympus). The samples obtained from 3 individuals were performed for each hybrid variety, and quantitative data on HE staining were collected from them.

## Results

### Genome assembly

A total of 8 species of cyprinid fishes from East Asia were sequenced using PacBio HiFi or Oxford Nanopore technology, resulting in over 602.21 Gb of raw data (Supplementary  Table S1). *De novo* assembled genomes were obtained with contig N50 ranging from 7.57 to 38.12 Mb. Chromosome-scale genomes were assembled for blunt snout bream (*Megalobrama amblycephala*, BSB) and topmouth culter (*Culter alburnus*, TC) using 204.8 Gb Hi-C data. The resulting assemblies exhibited scaffold N50 values of 42.91 Mb and 39.60 Mb, respectively (Table 1 and Supplementary  Tables S1–S2). Assembly quality was assessed using BUSCO, scoring between 94.5% and 98.7% (Supplementary  Table S3). We significantly improved the genome assemblies for both *M. amblycephala* (contig N50 increased from 2.4 to 15.42 Mb [57]) and *C. alburnus* (contig N50 increased from 17.8 to 18.55 Mb [58]). High-quality genome data for *Cirrhinus molitorella, Pseudorasbora parva*, and *Xenocypris davidi* were presented for the first time. Through a combination of *de novo*, protein homology, and cDNA-based prediction, we annotated 26,550 and 27,303 protein-coding genes for *M. amblycephala* and *C. alburnus*, respectively (Supplementary  Tables S4–S5). Repetitive elements comprised 51.81% (568.18 Mb) and 50.49% (544.26 Mb) of the assemblies for *M. amblycephala* and *C. alburnus*, respectively (Supplementary  Table S6). Noncoding RNA was predicted in 8.76% and 7.21% of the genome assemblies for *M. amblycephala* and *C. alburnus*, respectively (Supplementary  Table S7). Furthermore, we obtained high-quality assembled genomes for 15 cyprinid fishes (with an average scaffold N50 of 34.39 Mb) and *Beaufortia kweichowensis* from public databases (Supplementary  Table S8). These 23 cyprinid fishes represent 7 nonpolyploid subfamilies (*Danioninae, Xenocyprinae, Gobioninae, Leuciscinae, Cultrinae, Labeoninae*, and *Hypophthalmichthyinae*) and 3 polyploid subfamilies (*Schizothoracinae, Barbinae*, and *Cyprininae*), with genome sizes ranging from 0.86 to 1.90 Gb and chromosome numbers varying widely from 48 to 150 (Table 1, Supplementary  Tables S1–S2, and S8).

**Table 1:** Assembly statistics of 8 species of cyprinid fishes

Species	Common name	Subfamily	Scaffold N50 (Mb)	Contig N50 (Mb)	Contig length (Mb)	BUSCO completeness (%)
*Megalobrama amblycephala*	Blunt snout bream	*Cultrinae*	42.91	15.42	1,096.68	94.5%
*Culter alburnus*	Topmouth culter	*Cultrinae*	39.60	18.55	1,077.98	98.3%
*Ctenopharyngodon idella*	Grass carp	*Leuciscinae*	/	35.62	901.49	98.5%
*Cirrhinus molitorella*	Mud carp	*Labeoninae*	/	38.12	1,066.79	98.6%
*Pseudorasbora parva*	Stone moroko	*Gobioninae*	/	7.57	1,292.12	98.5%
*Xenocypris davidi*	Bleeker’s yellow tail	*Xenocyprinae*	/	38.11	1,044.52	98.7%
*Gobiocypris rarus*	Raregudgeon	*Danioninae*	/	13.24	1,108.08	98.3%
*Elopichthys bambusa*	Yellow cheek carp	*Leuciscinae*	/	30.38	863.54	98.4%

### Phylogenomic analyses and introgression

**Figure 1:**
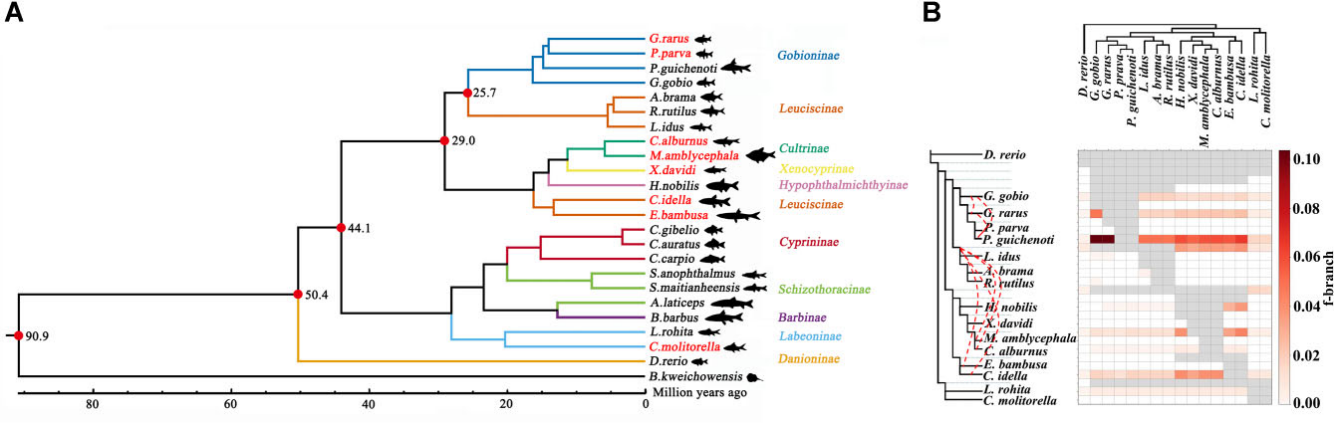
Phylogenomic analyses of cyprinid fish. (A) Time-calibrated phylogenetic tree. Red pot represents fossil calibration time points, which were obtained from “Timetree of Life.” Black number in the branch represents the median of the range of divergence times. The name marked in red represents the species sequenced in this study. (B) Gene flow determined using the f-branch method. Dashed red line represents gene flow (f4-ratio > 0.05) between 2 species.

To investigate the evolutionary relationships among extant cyprinids, we analyzed 24 genomes with butterfly hillstream loach (*B. kweichowensis*) as an outgroup (Fig. 1A and Supplementary  Fig. S1). Our results showed that *Gobiocypris rarus* belongs to the subfamily *Gobioninae*, even though from a morphological perspective, it appears similar to zebrafish (which belongs to the subfamily *Danioninae* of Cyprinidae). Molecular clock analysis with fossil calibration indicated their divergence time ranging from 41.5–61.3 MYA (Fig. 1A and Supplementary  Table S9). Phylogenetic trees reconstructed the evolutionary history of subfamily *Leuciscinae*, showing that 1 group (including *Leuciscus idus, Abramis brama*, and *Rutilus rutilus*) diverged from another group (*Ctenopharyngodon idella* and *Elopichthys bambusa*) ranging from 23.8 to 35.1 MYA. This divergence occurred earlier than the divergence times observed among other subfamilies (*Cultrinae, Gobioninae, Xenocyprinae*, and *Hypophthalmichthyinae*) (Fig. 1A and Supplementary  Table S9). Furthermore, phylogenomics analysis provided evidence regarding the divergence of common ancestors of extant cyprinids, which ranged from 81.9 to 100.0 MYA (Fig. 1A and Supplementary  Table S9). The ancestor of *Danio rerio* (subfamily: *Danioninae*) diverged early in the evolution of extant cyprinids, while the ancestor of *Cirrhinus molitorella* and *Labeo rohita* (subfamily: *Labeoninae*) diverged between 36.5 and 53.6 MYA (Fig. 1A and Supplementary  Table S9). These findings will assist us in understanding the evolutionary process of fish and constructing more reasonable classification relationships within the cyprinids, with the support of data from fields such as fossils, monsoons, and geography [59, 60].

Previous studies have reported phylogenetic discordance across genome regions in nonpolyploid cyprinid fishes from the East Asian region. This discordance has been attributed to incomplete lineage sorting, introgression, and the fish’s demographic history [61]. To investigate this, we conducted gene flow analysis and observed pervasive introgression among the 17 nonpolyploid cyprinid fishes (f4-ratio > 0.0006, *z*-score > 3, and *P* < 0.05) (Fig. 1B). The ABBA-BABA tests [49, 62] revealed strong gene flow events among subfamily *Gobioninae*, including *Paracanthobrama guichenoti* and *Gobiocypris rarus* (f4-ratio = 0.1, *z*-score = 129, and *P* < 0.001), *G. rarus* and *Gobio gobio* (f4-ratio = 0.1, *z*-score = 120, and *P* < 0.001), and *P. guichenoti* and *G. gobio* (f4-ratio = 0.1, *z*-score = 96, and *P* < 0.001) (Fig. 1B and Supplementary  Table S10). Notably, distinct gene flow signals (f4-ratio > 0.05) between 2 species were predominantly detected in 22 groups involving 12 species and 7 subfamilies (*Cultrinae, Danioninae, Gobioninae, Hypophthalmichthyinae, Leuciscinae_1, Leuciscinae_2*, and *Xenocyprinae*) (red dotted line in Fig. 1B and Supplementary  Table S10). Recent studies utilizing mitochondrial genomes and *de novo* nuclear genomes have also highlighted frequent gene flow events during the radiation of cyprinid fishes [60]. Our findings, based on high-quality genome assemblies, support the hypothesis that gene flow is the primary driver of the observed phylogenetic incongruence among nonpolyploid cyprinid fishes.

### Conservation of the reproductive system in speciation

**Figure 2:**
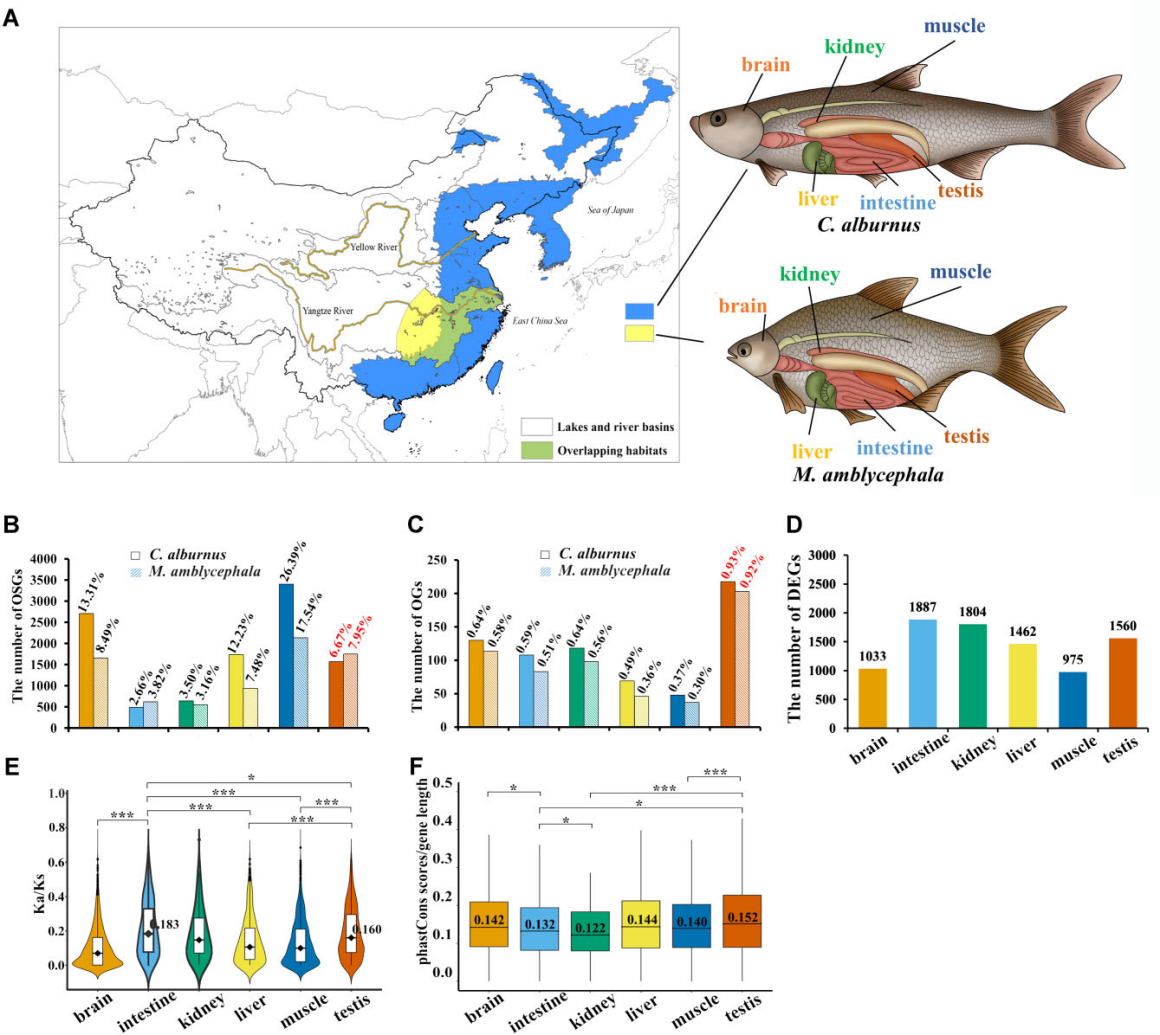
Divergent evolution of *M. amblycephala* and *C. alburnus* in East Asia. (A) Habitat distribution of extant *M. amblycephala* [65] and *C. alburnus*. Partial overlap in the habitats of the 2 species. (B) The number and percentage of OSGs in *M. amblycephala* and *C. alburnus*. (C) The number and percentage of OGs in *M. amblycephala* and *C. alburnus*. (D) Differential expression between *M. amblycephala* and *C. alburnus* in 6 tissues and organs. (E) The distribution of Ka/Ks values relating to OSGs in the 6 tissues and organs. The median value (black dot) was signed in the figure. (F) Conserved scores of OSGs in the 6 tissues and organs. The median value (black line and number) was signed in the figure. The comparisons involve the intestine and testis. “*” represents “0.01 < *P* ≤ 0.05,” “**” represents “0.001 < *P* ≤ 0.01,” and “***” represents “*P* ≤ 0.001.”

The frequent occurrence of gene flow events between cyprinid fishes, including *M. amblycephala* and *C. alburnus*, suggests incomplete reproductive isolation as a potential contributing factor to their speciation in the East Asian region. To investigate the underlying genetic mechanisms, we focused on comparing the genomes of these 2 species, which belong to different genera within the *Cultrinae* subfamily but share overlapping habitats in the middle and lower reaches of the Yangtze River Basin (Fig. 2A). While laboratory experiments have indicated some degree of postzygotic isolation, no natural hybrid populations have been identified in the wild [63]. A conserved synteny analysis revealed a high degree of gene conservation between *M. amblycephala* and *C. alburnus* (Supplementary  Figs. S2–S3), making them a suitable model for studying the genetic basis of their speciation.

To investigate the genetic differences between *M. amblycephala* and *C. alburnus*, we conducted comparative gene expression analyses in 6 tissues: brain, liver, intestine, muscle, kidney, and testis (Supplementary  Tables S11–S12). Our findings revealed that the testis exhibited a higher proportion of OSG, accounting for 6.67% in *C. alburnus* and 7.95% in *M. amblycephala*, compared to the intestine and kidney (Fig. 2B). We focused on OGs [64] and identified a greater number of OGs in the testis of both species, suggesting rapid divergence in this organ (Fig. 2C and Supplementary  Table S13). However, the number of differentially expressed genes (DEGs) between the 2 species was lower in the testis than in the 3 tissues (Fig. 2D, Supplementary  Fig. S4, and Supplementary  Tables S14–S16). To explore functional divergence, we calculated Ka/Ks values for OSGs and found that the testis exhibited lower Ka/Ks values compared to the intestine (*t*-test: *P* = 0.04) but higher values compared to the 3 tissues (liver, brain, and muscle, *t*-test: *P* < 0.001) (Fig. 2E). Similar patterns were observed for DEGs and the shared genes (OSGs and DEGs) (Supplementary  Fig. S5). Finally, to assess the degree of sequence conservation [47], we calculated phastCons scores of OSGs and observed the highest median value in the testis, which was higher than in the intestine, kidney, and muscle (*t*-test: *P* < 0.05) (Fig. 2F). A similar phenomenon was noted when analyzing DEGs using phastCons scores (Supplementary  Fig. S6). *M. amblycephala* and *C. alburnus* exhibited lower genetic variation in their testes compared to their intestines.

### Rapid evolution of digestive system in speciation

The diverse digestive systems of cyprinid fishes allow them to adapt to various food sources, including plankton, aquatic plants, and benthic organisms, contributing to their ecological niche differentiation [66]. Differential expression analyses revealed that the intestine of *C. alburnus* had the lowest number of OSGs (752, 4.15%), while *M. amblycephala*’s intestine had the second lowest (942, 5.95%) (Fig. 2B). The number of OGs in the intestine (0.59% in *C. alburnus* and 0.51% in *M. amblycephala*) was lower compared to the testis, brain, and kidney (Fig. 2C). The study demonstrates that the genetic makeup of the intestine exhibits a higher degree of conservation compared to the other tissues and organs. However, rapid genetic divergence between the herbivorous *M. amblycephala* and the carnivorous *C. alburnus* was observed in their intestines. For instance, the highest number of DEGs between the 2 species was detected in their intestines (Fig. 2D and Supplementary  Tables S14–S15). Moreover, the Ka/Ks values of OSGs were higher in the intestine compared to the brain, kidney, muscle, and testis (Fig. 2E). Lastly, the phastCons scores of OSGs in the intestine were lower than those in the brain, liver, muscle, and testis, although they were higher than in the kidney (Fig. 2F). Similar trends were observed in the phastCons scores of DEGs (Supplementary  Fig. S6). Our findings provide preliminary evidence suggesting a potential rapid divergence in genetic diversity within the digestive organs of *M. amblycephala* and *C. alburnus*.

**Figure 3:**
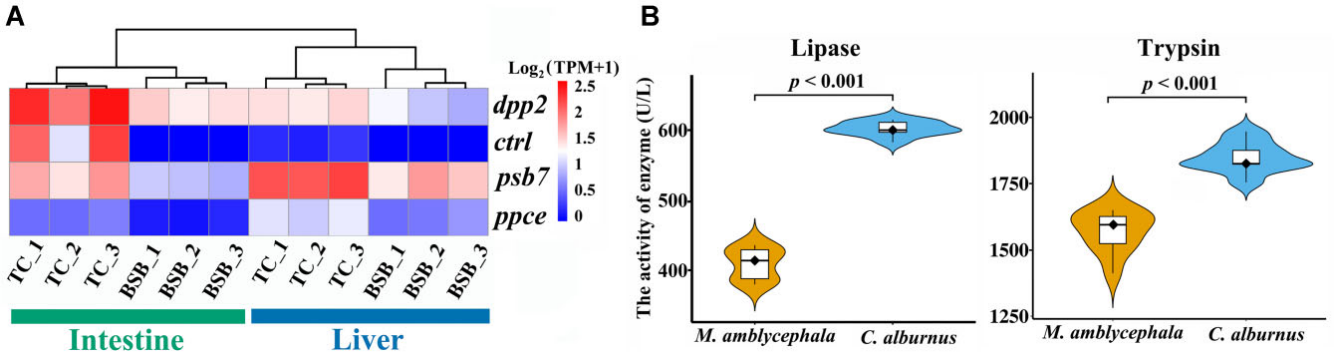
Diet divergences between *M. amblycephala* and *C. alburnus*. (A) The 4 genes relating to differential expression between *M. amblycephala* (BSB) and *C. alburnus* (TC) in both the intestine and liver (3 biological replicates showed “_1,” “_2,” and “_3”). (B) Significant differences in the enzyme activity of lipase and trypsin for the comparison between *M. amblycephala* and *C. alburnus*.

To investigate the genetic basis of diet divergence between herbivorous *M. amblycephala* and carnivorous *C. alburnus*, we conducted a functional analysis of their DEGs in the intestine. These genes associated with digestive enzymes were enriched for hydrolyzing O-glycosyl compounds (GO: 0004553) and peptidase activity (GO: 0008233) in terms of Molecular Function annotation, while carbohydrate metabolic process (GO: 0005975) and lipid catabolic process (GO: 0016042) were enriched for Biological Process annotation (Supplementary  Fig. S7). Among these genes, *dpp2, ctrl, psb7*, and *ppce* were identified as potential genes involved in peptidase activity, exhibiting higher expression in the digestive organs (liver and intestine) of *C. alburnus* compared to *M. amblycephala* (Fig. 3A). After detecting the enzyme activities of trypsin and lipase in digestive organs, we found that the enzyme activities were higher in the carnivorous *C. alburnus* compared to the herbivorous *M. amblycephala* (Fig. 3B). We conducted analyses on positively selected genes (PSGs) (Ka/Ks > 1) between the 2 species and identified 30 of them that belong to OSGs in the 6 tissues and organs (Supplementary  Table S17). Among the share genes of PSGs and OSGs in the intestine, *caspbl* and *vsig* were found to be associated with peptidase activity (GO: 0008233), apoptosis, and immune responses, which are closely related to the types of digested food (Supplementary  Fig. S8) [67, 68]. These findings suggest that the observed genetic diversity may be related to adaptations in digestive enzyme secretion, reflecting potential dietary adjustments.

### Gene flow and its potential impact on feeding habits

**Figure 4:**
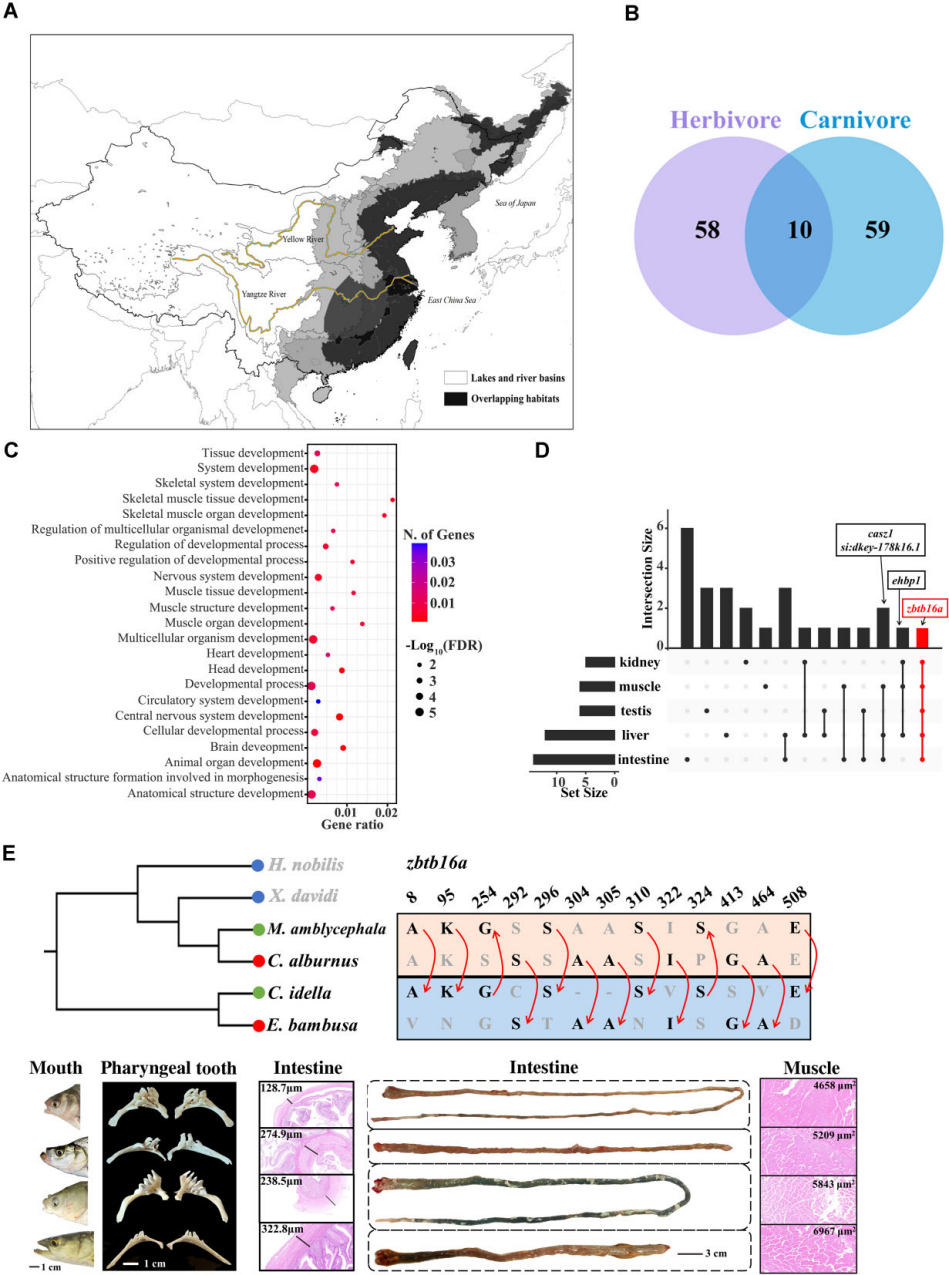
Gene flow in herbivorous and carnivorous fishes. (A) Habitat distribution of *M. amblycephala* [65], *C. alburnus, C. idella*, and *E. bambusa* in East Asia. Their overlap and unique habitats reflect their speciation of endemic East Asian cyprinid fishes in the river-lake ecosystems of East Asia. (B) Venn diagram showing introgressed genes between carnivores and herbivores. (C) The introgressed genes were expressed in different tissues and organs, while there was no introgressed gene expressed in the brain. (D) GO analyses of the shared 10 introgressed genes in carnivores and herbivores. (E) Phylogenomic analyses of the 6 endemic cyprinid species in China; introgressions visualizing at SNPs of *zbtb16a* (potential introgressed SNPs marking red arrow); the morphologies of mouth, pharyngeal teeth, and intestine; microstructure of intestine (thickness of intestinal wall marking in figure); and muscle (average area marking in figure) in the 4 endemic cyprinid species. Blue dot represents filter-feeding fish, green dot represents herbivorous fish, and red dot represents carnivorous fish.

Frequent gene flow events were observed among cyprinid fishes, including *M. amblycephala, C. alburnus, C. idella*, and *E. bambusa*. Among these, significant gene flow was detected between carnivorous *C. alburnus* (subfamily *Cultrinae*) and *E. bambusa* (subfamily *Leuciscinae*) (*z*-score > 45.8, f4-ratio = 0.039, and *P* < 0.001). Additionally, gene flow event was identified between the herbivorous *M. amblycephala* (subfamily Cultrinae) and *C. idella* (subfamily *Leuciscinae*) (*z*-score > 38.3, f4-ratio = 0.044, and *P* < 0.001) (Fig. 1B and Supplementary  Table S10). The overlapping habitats of these 4 species were primarily distributed in the eastern region of China (Fig. 4A).

To investigate the effects of gene flow events on diet diversity, we analyzed the 117 introgressed genomic regions (window size: 20 kb) between the 2 carnivorous fish species, which were associated with 69 genes (Supplementary  Table S18). Similarly, the 102 introgressed regions (window size: 20 kb) between the 2 herbivorous fish species were associated with 68 genes (Supplementary  Table S19). Among these genes, the top 3 molecular function annotations in both carnivores and herbivores were related to transcription regulator activity (GO: 0140110), DNA-binding transcription factor activity (GO: 0003700), and RNA polymerase II–specific activity (GO: 0000981) (Supplementary  Tables S20–S21). Among these genes, 10 introgressed genes were shared between carnivores and herbivores (Fig. 4B). Moreover, 84 categories (38.36%) for biological processes and 16 categories (66.67%) for molecular functions were shared between carnivores and herbivores false discovery rate (FDR < 0.05, Fig. 4C and Supplementary  Figs. S9–S10). These shared introgressed genes were associated with animal organ development, including skeletal muscle organ development (GO: 0060538; *sox6* and *ttn.2*) and head development (GO: 0060322; *zfhx3, tcf7l2*, and *meis1b*) (Supplementary  Fig. S11). *Zbtb16a* (linked to osteogenic differentiation [69]) exhibited the broadest expression pattern among all introgressed genes, being detected in 5 different tissues and organs (Fig. 4D). This suggests that *zbtb16a* may be a hotspot for introgression events between *Cultrinae* and *Leuciscinae*.

There were potential relationships between diet habits and organ development, including mouth and pharyngeal tooth morphologies, intestinal morphology, and skeletal muscle structure (Fig. 4E). Comparative analyses revealed that carnivorous fishes exhibited larger and superior mouths, longer and sharper teeth, shorter intestines, thinner intestine linings, and smaller cross-sectional areas in skeletal muscle fibers compared to herbivorous fishes (Fig. 4). It is noteworthy that some of the introgressed genes have been experimentally validated in zebrafish to have functional associations with dietary traits. For instance, *tp53* and *tle3a* are implicated in intestinal morphology [70, 71], while *grin2bb* and *grin1a* are linked to food intake behavior [72, 73]. Additionally, *znf536, zfhx3, elavl4, hoxc11a, pik3r3b*, and *irf2bpl* have been identified as potential regulators of swimming behavior. These findings suggest that these introgressed genes may relate to organ development and feeding behavior may contribute to diet divergence for these fishes.

## Discussion

The East Asian region, characterized by its unique topography, including the uplift of the Qinghai–Tibet Plateau, abundant rivers, and diverse climatic environments, is home to a multitude of freshwater fish species [60, 74, 75]. Cyprinids represent the largest and most diverse vertebrate group, with over 654 species, including 440 endemics in China [76]. Investigating the genetic mechanisms underlying their rapid speciation is vital for understanding evolutionary radiation in East Asian cyprinids. Our findings suggest that frequent gene flow events among cyprinid fishes have contributed to their rapid adaptive radiation, as evidenced by our analyses of 7 nonpolypoid subfamilies. This prompts the question: How does introgressive hybridization in East Asian cyprinids relate to rapid speciation?

Rapid evolutionary changes in the mammalian testis manifest at the molecular level, contributing to reproductive isolation. Comparative gene expression studies across various mammalian organs reveal that the testis exhibits the highest rates of evolutionary expression change [77, 78]. Therefore, comparative genomic analysis of *M. amblycephala* and *C. alburnus* revealed a lower degree of genetic divergence in the testis compared to the intestine. Additionally, laboratory experiments have demonstrated the production of fertile hybrids between various cyprinid species [13, 63], suggesting the possibility of incomplete RI and the prevalence of introgressive hybridization in East Asian cyprinids. However, further research is needed to elucidate the specific factors hindering and driving rapid speciation in these cyprinid fishes.

The complex and variable inland water ecosystem plays a crucial role in the adaptive evolution of fish [79, 80]. Among these factors, the diversity of food sources gradually influences the feeding habits of different populations, resulting in the adaptive evolution of their digestive and locomotion systems [81]. Our findings suggest that speciation in *M. amblycephala* and *C. alburnus* may have been driven by diet-dependent adaptations. Cyprinid fishes display significant diversity in behavior, habitat, geography, and morphology, including variations in feeding and digestive organs [60, 76]. Robust pharyngeal teeth and toothless jaws enable them to consume a wide range of foods [82, 83]. Our results reveal that rapid genetic divergence between *M. amblycephala* and *C. alburnus* occurs in the intestine. The variations in digestive enzyme secretion and digestive organs reflect their distinct feeding preferences and the effectiveness with which they metabolize different food types [84]. Considering the absence of postzygotic isolation and the overlapping habitat between *M. amblycephala* and *C. alburnus* [63, 85], our results suggest that ecological differentiation driven by dietary differences may be an important factor leading to the rapid formation of these 2 species.

When postzygotic reproductive isolation is no longer a significant barrier to gene flow among East Asian cyprinids, natural selection, including monsoon activities [60], and the uplift of the Qinghai–Tibet Plateau [75], can attenuate prezygotic isolation, providing opportunities for gene flow, thus promoting speciation in cyprinid fishes [86]. To adapt to diverse food supplies in different aquatic environments, feeding habits have diverged in the subfamilies *Cultrinae* (herbivorous *M. amblycephala* and carnivorous *C. alburnus*) and *Leuciscinae* (herbivorous *C. idella* and carnivorous *E. bambusa*). Does gene flow facilitate the divergence of diets for adaptive evolution? Our results demonstrate the introgression of genes associated with skeletal muscle and head development between fishes with the same diet. These changes play crucial roles in feeding and digestive efficiency. Fishes with the same diet in different subfamilies exhibit similar phenotypes involving the mouth, teeth, intestine, and muscle. These results suggest that coevolving interactions of diet habits occur in their speciation through introgressive hybridization. However, further evidence is needed to establish a definitive association between dietary convergent evolution and gene flow.

## Supplementary Material

giae117_GIGA-D-24-00199_Original_Submission

giae117_GIGA-D-24-00199_Revision_1

giae117_Response_to_Reviewer_Comments_Original_Submission

giae117_Reviewer_1_Report_Original_Submission Zhanjiang Liu -- 8/7/2024 Reviewed

giae117_Reviewer_2_Report_Original_Submission Liandong Yang -- 9/3/2024 Reviewed

giae117_Reviewer_2_Report_Revision_1 Liandong Yang -- 10/21/2024 Reviewed

giae117_Reviewer_3_Report_Original_Submission Trevor Krabbenhoft -- 9/30/2024 Reviewed

giae117_Supplemental_Figures_and_Tables

## Data Availability

Genomic sequencing data obtained from PacBio HiFi, Oxford Nanopore, and DNBSEQ-T7 technologies, as well as Hi-C data, have been submitted to the National Center for Biotechnology Information (NCBI) (accession numbers: SRR26190421–SRR26190427, SRR26139312–SRR26139313, SRR26139214–SRR26139215, and SRR26319599–SRR26319600). The assembled genome and annotation files of 8 cyprinid fishes have been deposited on Figshare [87] and the National Genomics Data Center (NGDC) (accession numbers: GWHDOEU00000000, GWHDOEX00000000, GWHDOEV00000000, GWHDOEW00000000, GWHDOEB00000000, GWHDOEC00000000, GWHDOES00000000, and GWHDOET00000000). The raw reads of the mRNA-seq data have been submitted to NGDC (accession number: subCRA017373) and NCBI (accession numbers: SRR26087118–SRR26087153). All additional supporting data are available in the *GigaScience* repository, GigaDB [88–96].

## Additional Files

**Supplementary Table S1**. Summary of whole genome sequencing in 8 species.

**Supplementary Table S2**. Genome assembly of 8 species.

**Supplementary Table S3**. Completeness of the 8 assembled genomes.

**Supplementary Table S4**. Statistics of gene prediction.

**Supplementary Table S5**. Gene function annotation of *M. amblycephala* and *C. alburnus*.

**Supplementary Table S6**. Summary of repeat contents.

**Supplementary Table S7**. The summary of predicted noncoding RNA in *M. amblycephala* and *C. alburnus*.

**Supplementary Table S8**. Information of downloaded genomes.

**Supplementary Table S9**. Range of divergence time in Fig. 1.

**Supplementary Table S10**. D statistic on the species tree based on genome-wide single-nucleotide polymorphisms (SNPs). The outgroup was fixed as *Beaufortia kweichowensis*.

**Supplementary Table S11**. Summary of transcriptome sequencing data.

**Supplementary Table S12**. Summary of transcriptome mapping data.

**Supplementary Table S13**. List of orphan genes (OGs) in *M. amblycephala* and *C. alburnus*.

**Supplementary Table S14**. Summary of differentially expressed genes (DEGs) between *M. amblycephala* and *C. alburnus* in 6 organs.

**Supplementary Table S15**. The gene number of orthologous gene pairs and differential expressed genes (DEGs) in 6 organs.

**Supplementary Table S16**. The summary of organ-specific genes (OSGs), positive selective genes (PSGs), and differential expressed genes (DEGs) in 6 organs.

**Supplementary Table S17**. Summary of expressed positive selective genes (PSGs) between *C. alburnus* (TC) and *M. amblycephala* (BSB) in the 6 organs.

**Supplementary Table S18**. Summary of gene flow between carnivorous *C. alburnus* and *E. bambusa*.

**Supplementary Table S19**. Summary of gene flow between herbivorous *M. amblycephala* and *C. idella*.

**Supplementary Table S20**. GO enrichment of introgressed genes between carnivorous *C. alburnus* and *E. bambusa*.

**Supplementary Table S21**. GO enrichment of introgressed genes between herbivorous *M. amblycephala* and *C. idella*.

**Supplementary Fig. S1**. Phylogenetic trees constructed using multiple whole-genome alignments of 17 (no polyploid species) and 24 (including 7 polyploid species) species with *Beaufortia kweichowensis* as the root, respectively. (A) Concatenation-based method for estimating a phylogenetic tree of 17 species with 10-kb length windows. (B) Coalescent method for estimating a phylogenetic tree of 17 species with 10-kb length windows. (C) Concatenation-based method for estimating a phylogenetic tree of 24 species with 10-kb length windows. (D) Coalescent method for estimating a phylogenetic tree of 24 species with 10-kb length windows. (E) Species tree with estimated divergence time.

**Supplementary Fig. S2**. The Hi-C interaction heatmap of 24 linkage groups in the genomes of *M. amblycephala* and *C. alburnus*.

**Supplementary Fig. S3**. The collinearity analysis between *M. amblycephala* and *C. alburnus*. Twenty-four pairs of homologous chromosomes were determined based on 17,337 orthologous gene pairs.

**Supplementary Fig. S4**. The differential expression between *M. amblycephala* and *C. alburnus* in 6 organs. Upregulated genes in *M. amblycephala* are marked in blue, while the upregulated genes in *C. alburnus* are marked in red.

**Supplementary Fig. S5**. The distribution of Ka/Ks values in the 6 organs. (A) The Ka/Ks values of DEGs. (B) The Ka/Ks values of shared genes between DEGs and OSGs. The median value is indicated by a black dot, and the gene number is provided below each organ name. In the *t*-test, “*” represents 0.01 < *P* ≤ 0.05, “**” represents 0.001 < *P* ≤ 0.01, and “***” represents *P* ≤ 0.001.

**Supplementary Fig. S6**. Conserved scores of DEGs (*M. amblycephala* vs. *C. alburnus*) in the 6 organs. “*” represents 0.01 < *P* ≤ 0.05, “**” represents 0.001 < *P* ≤ 0.01, and “***” represents *P* ≤ 0.001.

**Supplementary Fig. S7**. The DEGs (*M. amblycephala* vs. *C. alburnus*) associated with diet habit. (A) The heatmap of the DEGs in the intestine. The hydrolyzing O-glycosyl compounds and peptidase activity in Molecular Function, as well as carbohydrate metabolic process and lipid catabolic process in Biological Process. (B) The gene distribution of DEGs in the intestine.

**Supplementary Fig. S8**. The alignment of 2 positively selected genes (PSGs) in the intestine.

**Supplementary Fig. S9**. The GO terms of introgressed genes in the carnivorous (*C. alburnus* and *E. bambusa*) and herbivorous *(M. amblycephala* and *C. idella*) fishes.

**Supplementary Fig. S10**. The distribution of enriched functional categories (FDR < 0.05) in Biological Process and Molecular Function for the introgressed genes.

**Supplementary Fig. S11**. Heatmap exhibiting the expression of introgressed genes in the carnivorous (*C. alburnus* and *E. bambusa*) and herbivorous (*M. amblycephala* and *C. idella*) fishes.

## Abbreviations

BSB: blunt snout bream; DE: differential expression; DEGs: differentially expressed genes; MYA: million years ago; OGPs: orthologous gene pairs; OGs: orphan genes; OSG: organ-specific gene; PSGs: positively selected genes; RI: reproductive isolation; TC: topmouth culter; TPM: transcripts per million; TRF: tandem repeats finder.
