# Supplementary material for: Genomes reveal pervasive distant hybridization in nature among cyprinid fishes
Source: Gigascience. 2025 Jan 30;14:giae117. doi: 10.1093/gigascience/giae117 (PMC11779505; doi:10.1093/gigascience/giae117)
Supplement: giae117_GIGA-D-24-00199_Original_Submission [file giae117_giga-d-24-00199_original_submission.pdf]

GigaScience

Genomes reveal pervasive distant hybridization in nature among cyprinid fishes

--Manuscript Draft--

**Manuscript Number:**	GIGA-D-24-00199
**Full Title:**	Genomes reveal pervasive distant hybridization in nature among cyprinid fishes
**Article Type:**	Research

Funding Information:

National Natural Science Foundation of China (32293252)	Not applicable
National Natural Science Foundation of China (32341057)	Not applicable
National Natural Science Foundation of China (U19A2040)	Not applicable
Hunan Provincial Natural Science Foundation (2022JJ10035)	Not applicable
Huxiang Young Talent Project of China (2021RC3093)	Not applicable
National Key Research and Development Plan Program (2023YFD2401602)	Not applicable
Laboratory of Lingnan Modern Agriculture Project (NT2021008)	Not applicable
Special Funds for Construction of Innovative Provinces in Hunan Province (2021NK1010)	Not applicable
Earmarked Fund for China Agriculture Research System (CARS-45)	Not applicable
111 Project (D20007)	Not applicable

Abstract:

Background

Genomic data has unveiled a fascinating aspect of the evolutionary past, showing that the mingling of different species through hybridization has left its mark on the histories of numerous life forms. However, the relationship between hybridization events and the origins of cyprinid fishes remains unclear.

Results

In this study, we generated de novo assembled genomes of eight cyprinid fishes and conducted phylogenetic analyses on 24 species. Widespread allele sharing across species boundaries was observed within seven subfamilies of cyprinid fishes. Interestingly, we noted conservative divergence in the testis and incomplete reproductive isolation between the herbivorous Megalobrama amblycephala and the carnivorous Culter alburnus. Significant differences in the expression of four genes (dpp2, ctrl, psb7, and ppce) in the liver and intestine, accompanied by variations in enzyme activities, indicated swift divergence in digestive enzyme secretion. Moreover, we identified introgressed genes linked to organ development in sympatric fishes with analogous feeding habits within the Cultrinae and Leuciscinae subfamilies.

Conclusions

Our findings highlight the significant role played by incomplete reproductive isolation and frequent gene flow events, particularly those associated with the development of digestive organs, in driving speciation among cyprinid fishes in diverse freshwater ecosystems.

Powered by Editorial Manager® and ProduXion Manager® from Aries Systems Corporation

**Corresponding Author:**	Li Ren<br>Hunan Normal University<br>Changsha, Hunan CHINA
**Corresponding Author Secondary Information:**	
**Corresponding Author's Institution:**	Hunan Normal University
**Corresponding Author's Secondary Institution:**	
**First Author:**	Li Ren
**First Author Secondary Information:**	
**Order of Authors:**	Li Ren
	Xiaolong Tu
	Mengxue Luo
	Qinzhi Liu
	Jialin Cui
	Xin Gao
	Hong Zhang
	Yakui Tai
	Yiyan Zeng
	Mengdan Li
	Chang Wu
	Wuhui Li
	Jing Wang
	Dongdong Wu
	Shaojun Liu
**Order of Authors Secondary Information:**	

**Additional Information:**	
**Question**	**Response**
Are you submitting this manuscript to a special series or article collection?	No
**Experimental design and statistics**<br><br>Full details of the experimental design and statistical methods used should be given in the Methods section, as detailed in our Minimum Standards Reporting Checklist. Information essential to interpreting the data presented should be made available in the figure legends.<br><br>Have you included all the information requested in your manuscript?	Yes

**Resources**<br><br>A description of all resources used, including antibodies, cell lines, animals and software tools, with enough information to allow them to be uniquely identified, should be included in the Methods section. Authors are strongly encouraged to cite Research Resource Identifiers (RRIDs) for antibodies, model organisms and tools, where possible.<br><br>Have you included the information requested as detailed in our Minimum Standards Reporting Checklist?	Yes
**Availability of data and materials**<br><br>All datasets and code on which the conclusions of the paper rely must be either included in your submission or deposited in publicly available repositories (where available and ethically appropriate), referencing such data using a unique identifier in the references and in the "Availability of Data and Materials" section of your manuscript.<br><br>Have you have met the above requirement as detailed in our Minimum Standards Reporting Checklist?	Yes

Manuscript Click here to access/download;Manuscript;manuscript.docx

Genomes reveal pervasive distant hybridization in nature among cyprinid fishes

Li Ren[1,*], Xiaolong Tu[3,4,5,*], Mengxue Luo[1,*], Qinzhi Liu[1,*], Jialin Cui[1], Xin Gao[1], Hong Zhang[1], Yakui Tai[1], Yiyan Zeng[1], Mengdan Li[1], Chang Wu[1], Wuhui Li[1], Jing Wang[1], Dongdong Wu[3,4,†] and Shaojun Liu[1, 2,†]

[1]State Key Laboratory of Developmental Biology of Freshwater Fish, Engineering Research Center of Polyploid Fish Reproduction and Breeding of the State Education Ministry, College of Life Sciences, Hunan Normal University, Changsha, 410081, China.

[2]Guangdong Laboratory for Lingnan Modern Agriculture, South China Agricultural University, Guangzhou, 510642, China.

[3]State Key Laboratory of Genetic Resources and Evolution, Kunming Institute of Zoology, Chinese Academy of Sciences, Kunming, 650201, China.

[4]Kunming Natural History Museum of Zoology, Kunming Institute of Zoology, Chinese Academy of Sciences, Kunming, 650223, China.

[5]Kunming College of Life Science, University of the Chinese Academy of Sciences, Kunming, 650204, China.

†Corresponding author, Email: lsj@hunnu.edu.cn; wudongdong@mail.kiz.ac.cn

*These authors contributed equally to this work.

Abstract

Background: Genomic data has unveiled a fascinating aspect of the evolutionary past, showing that the mingling of different species through hybridization has left its mark on the histories of numerous life forms. However, the relationship between hybridization events and the origins of cyprinid fishes remains unclear.

Results: In this study, we generated *de novo* assembled genomes of eight cyprinid fishes and conducted phylogenetic analyses on 24 species. Widespread allele sharing across species boundaries was observed within seven subfamilies of cyprinid fishes. Interestingly, we noted conservative divergence in the testis and incomplete reproductive isolation between the herbivorous *Megalobrama amblycephala* and the carnivorous *Culter alburnus*. Significant differences in the expression of four genes (*dpp2*, *ctrl*, *psb7*, and *ppce*) in the liver and intestine, accompanied by variations in enzyme activities, indicated swift divergence in digestive enzyme secretion. Moreover, we identified introgressed genes linked to organ development in sympatric fishes with analogous feeding habits within the *Cultrinae* and *Leuciscinae* subfamilies.

Conclusions: Our findings highlight the significant role played by incomplete reproductive isolation and frequent gene flow events, particularly those associated with the development of digestive organs, in driving speciation among cyprinid fishes in diverse freshwater ecosystems.

Keywords: diet divergence, genetic introgression, phylogenomics, incomplete reproductive isolatio

Introduction

Cyprinidae (Order Cypriniformes) is the largest and most diverse family of ray-finned fish, comprising about 13 subfamilies and 370 genera [1, 2]. This family includes popular aquarium fish like goldfish and koi, as well as the valuable vertebrate model organism, the zebrafish [2]. As a family of freshwater fish, the origin time of Cyprinidae was estimated at 154 Mya [3] and they are now widely distributed in almost all types of water around the world [2]. Their great diversities in feeding and reproductive behaviors, as well as morphology, including body length (ranging from about 8 mm for *Paedocypris progenetica* to approximately 3 m for *Catlocarpio siamensis*) [4, 5] and digestive organs [6, 7] are intriguing to evolutionary biologists due to their phylogenetic relationships and adaptive radiation evolution. However, the narrow distribution ranges or small population sizes of Cyprinidae fish now face threats from human activity, such as overfishing, damming of upland rivers, pollution, habitat destruction, and novel viral infections [8].

Hybridization, or introgression, plays a significant and frequent role in adaptive evolution [9]. At least 10% of wild animals are hybrids, although most of them have low viability or are sterile [10]. Reproductive isolation (RI) is a common occurrence in intergeneric hybridization among birds and mammals. However, there are instances where this isolation is found to be disrupted within cyprinid fish species [11-13]. Natural selection, including variable water environments and deleterious homozygosity for small population sizes, brings great pressures to the survival of freshwater fish [14, 15]. Hybridization has the potential to generate genetic diversity and create opportunities for novel adaptive radiations, although it has been considered a breakdown of isolating mechanisms [16]. The low viability or sterility of hybrids could reinforce RI through selection for assortative mating and result in adaptive introgression [10]. The rate of introgression depends on the pressures of the freshwater environment and affects fish biodiversity [17]. Natural hybridization involving intergeneric hybridization was always observed among cyprinid fishes [18-20], while bisexual fertile progenies were detected in the laboratory experiments of various hybrid groups [13]. This evidence

suggests that prezygotic isolation evolves more rapidly than postzygotic isolation in cyprinid fishes, challenging the assumption of the criticality of variation in dietary niche breadth for speciation. Now, the relationship between biodiversity and introgressive hybridization in cyprinid fishes is still obscure. Phylogenomics from whole genome sequences could provide us with more detailed evidence of it than the fragmented detective technologies, including ribosomal DNA (rDNA) [20], mtDNA, and microsatellites [21].

Diet plays a crucial role in the biodiversity and habitat distribution of fishes, and is influenced by differences in foraging behavior and digestive organ morphology [22]. The selection of diet is particularly important for sympatric species [23]. In the case of cyprinid fishes, four main categories of diet have been identified. These include herbivorous fishes (e.g. *Megalobrama amblycephala* and *Ctenopharyngodon idella*), carnivorous fishes (e.g. *Culter alburnus* and *Elopichthys bambusa*), filter-feeding fishes (e.g. *Hypophthalmichthys nobilis*), and omnivorous fishes (e.g. *Cyprinus carpio* and *Carassius auratus*) [24]. These fish species are distributed across different water layers to acquire various types of food resources. Cyprinid fishes have evolved unique adaptations for food digestion, such as advanced protrusible pharyngeal teeth, despite the absence of jaw teeth and stomachs [25]. The number and shape of teeth in cyprinid fishes exhibit significant variation and are used as phenotypic features for species classification [2]. In East African cichlids, the number of tooth rows on both jaws has been associated with specific feeding ecologies [26]. However, it remains unclear whether variations in the width of the dietary niche are critical for the biodiversity of cyprinid fishes.

In this study, we obtained the *de novo* assembled genome sequences of eight cyprinid fish species and conducted comparative genome analyses using a set of 24 high-quality assembled genome sequences. Through gene flow analyses, we investigated hybridization events and their contributions to the speciation of cyprinid fishes. Furthermore, we conducted evolutionary constraints analyses on various tissues and organs and investigated their divergence between *M.*

amblycephala and *C. alburnus*. Our findings emphasize the significance of gene flow events in the origin of cyprinid fishes and the functional divergence that drives speciation.

Methods

Sample collection

After spending twenty-four months in the same suitable environments following hatching, the three sexually mature male individuals of *Culter alburnus* and *Megalobrama amblycephala* were bred in the Engineering Center of Polyploid Fish Breeding of the National Education Ministry in Changsha, Hunan, China. Their parents were collected from the Yangtze River (coordinates 30°25'56" N, 114°50'32" E). The parents of *Gobiocypris rarus* were obtained from the Liu Sha River, Sichuan Province (coordinates 29°19'31" N, 10240'38" E). We also collected one individual of each fish species (*Cirrhinus molitorella, Pseudorasbora parva, Xenocypris davidi, Elopichthys bambusa*, and *Ctenopharyngodon idella*) from Dongting Lake, Hunan, China (coordinates 29°15'8" N, 112°50'24" E). These individuals were deeply anesthetized with 300 mg/L tricaine methanesulfonate (Sigma-Aldrich, St. Louis, MO, USA) for 10 min (20°C) in a separation tank. After confirming their deaths, the muscle, brain, liver, intestine, kidney, and testis of all samples were collected after dissection.

DNA isolation and whole genome sequencing

High-quality and high-molecular-weight genomic DNA was isolated from muscle based on the DNA extraction methods. The purification was performed using the QIAGEN® Genomic Kit based on the standard operating procedure. The degradation and contamination of the extracted DNA were detected using 1% agarose gels. Then, DNA purity was determined using the NanoDrop™ One UV-Vis spectrophotometer (Thermo Fisher Scientific, USA) with 260/280 and 260/230 ratios. DNA concentration was measured by the Qubit® 4.0 Fluorometer (Invitrogen, USA).

After quality checking, the genomic DNA of *C. alburnus* and *M. amblycephala* was randomly sheared using Megaruptor (Diagenode, Denville, NJ, USA). The sheared genomic DNA was enriched and purified using AMPure PB beads according to the manufacturer's recommendations (Pacific Biosciences, Menlo Park, CA, USA). Large DNA fragments were separated using BluePippin DNA Size Selection System. DNA damage repair and end-repair were performed. Barcoded overhang hairpin adapters were ligated to the fragment ends. The connection reaction was performed using Ligation Sequencing Kit. A constructed DNA library was quantified using Qubit. Lastly, sequencing was performed using Nanopore Sequencing.

Fifteen µg of DNA for the six fishes (*C. idella*, *C. molitorella*, *P. parva*, *X. davidi*, *G. rarus*, and *E. bambusa*) was used for the preparation of SMRTbell target-size libraries, which were constructed using PacBio's standard protocol (Pacific Biosciences, CA, USA) with 15 kb preparation solutions. The main steps for library preparation are listed below: 1) The genomic DNA was sheared using g-TUBEs (Covaris, USA); 2) an A-tailing reaction was used to form an overhang; 3) the fragments were ligated with the hairpin adaptor using the SMRTbell Express Template Prep Kit 2.1 (Pacific Biosciences); 4) the library was treated with nuclease and purified using AMPure PB Beads; and 6) the SMRTbell library was purified using PB beads. The high-quality library was checked for fragment size using the Agilent 2100 Bioanalyzer (Agilent Technologies, USA). Sequencing Primer V2 and Sequel II Binding Kit 2.1 were used for PacBio Sequel II sequencing.

The genomic DNA of *C. alburnus* and *M. amblycephala* was utilized for whole-genome re-sequencing. First, high-quality DNA samples were used to prepare single-stranded circular libraries. Subsequently, the circular libraries were transformed into DNA nanoballs (DNBs), which are spherical structures containing millions of copies of the circular DNA templates. Once the DNBs were formed, they were loaded onto patterned nanoarrays. Following the loading of DNBs onto the nanoarrays, combinatorial probe anchor synthesis sequencing was conducted. Finally, DNBSEQ-T7 sequencing was performed using a paired-end approach (150 bp × 2) in accordance with the standard

protocol.

Genome assembly and chromosomal organization

The adapter and low-quality bases of the two species were filtered before assembly using Fastp (v. 0.21.0) [27]. All clean reads of *C. alburnus* and *M. amblycephala* were used for genome assembly using Nextdenovo (v. 2.3.0) [28]. The parameters "random_round = 20, minimap2_options_cns = -x ava-ont -t 40 -k17 -w17, and nextgraph_options = -a 0" were used in genome assembly. The base errors (SNV/Indel) in the genome generated were fixed using Nextpolish (v. 1.3.0) [29]. For the six fishes (*C. idella, C. molitorella, P. parva, X. davidi, G. rarus, and E. bambusa*), the HIFI data was used for genome assembly using hifiasm (0.15.4-r347) software [30].

Hi-C libraries of *C. alburnus* and *M. amblycephala* were created from muscle cells. Briefly, cells were fixed with formaldehyde and lysed, and the cross-linked DNA was digested with MobI. Sticky ends were biotinylated and proximity ligated to form chimeric junctions that were enriched for and then physically sheared to a size of 300–700 bp, as illustrated in Rao et al. [31]. Chimeric fragments representing the original cross-linked long-distance physical interactions were then processed into paired-end sequencing libraries. The clean reads of Hi-C were obtained from trimming of adapter sequences and low quality pair-end reads, which were truncated at the putative Hi-C junctions, and then the resulting trimmed reads were aligned to the assembly results with BWA (v. 0.7.17) [32]. Invalid read pairs, including Dangling-End and Self-cycle, Re-ligation, and Dumped products, were filtered by HiC-Pro (v. 2.8.1) [33]. They were used for the correction of scaffolds and the clustering, ordering, and orientation of scaffolds onto chromosomes by LACHESIS (release: 2017-12-21) [34]. After this step, placement and orientation errors exhibiting obvious discrete chromatin interaction patterns were manually adjusted.

Gene prediction and annotation

For protein-coding gene prediction in the genomes of *C. alburnus* and *M. amblycephala*, we

employed three integrated methods: *de novo* prediction, homology search, and cDNA-based prediction (muscle, brain, liver, intestine, kidney, and testis). *De novo* gene models were predicted using Augustus (v. 3.4.0) [35] with default parameters. In the homolog-based analysis, protein genes from five species (*C. carpio*: GCF_018340385.1, *C. auratus*: GCF_003368295.1, *O. macrolepis*: GCA_012432095.1, *P. tetrazona*: GCF_018831695.1, and *D. rerio*: GCF_000002035.6) obtained from NCBI were used to predict gene regions using GeneWise (v. 2.4.1) with default parameters. The cDNA-based approaches involved using Hisat2 (v. 2.1.0) [36] and TransDecoder (v. 5.5.0, https://github.com/TransDecoder/TransDecoder) software to predict open reading frames (ORFs). Subsequently, we integrated the results of genome annotation using GETA (v. 2.5.7, https://github.com/chenlianfu/geta). Gene functional predictions were assigned using Blast-2.11.0+ against public databases, including Swiss-Prot, the Non-Redundant Protein Sequence Database (NR, ftp://ftp.ncbi.nlm.nih.gov/blast/db/FASTA/), the KOG database (https://ftp.ncbi.nih.gov/pub/COG/KOG/), KO annotations were conducted with Kofamscan software (ftp://ftp.genome.jp/pub/tools/kofam_scan/), and motifs and domains were predicted using Hmmer [37] software against the PFAM database (http://ftp.ebi.ac.uk/pub/databases/Pfam/releases/Pfam33.1/).

To identify repetitive sequences, we utilized both *de novo*-based and homology-based methods. First, LTR_FINDER_parallel (v. 1.1) [38], LTRharvest (GenomeTools, v. 1.6.1) [39], LTR_retriever (v. 2.9.0) [40], and RepeatModeler (v. 2.0.1, http://www.repeatmasker.org/RepeatModeler/) software were employed to build a *de novo* repeat library, which was then merged with the Repbase database. RepeatMasker was subsequently used to predict repeat sequences using the new repeat library database. Tandem repeats were detected using Tandem Repeats Finder (TRF). For tRNA identification, we used tRNAscan-SE (v 2.0.7) [41], while rRNA was annotated using Blastn (BLAST v. 2.2.26, e-value: $1e^{-5}$) against the human rRNA sequence from the Rfam database. The snRNA and miRNA were searched using the Rfam database and the Infernal (v. 1.0.2,

https://github.com/EddyRivasLab/infernal) software.

Comparative phylogenomics

For the phylogeny analyses, we performed multiple whole-genome alignments (WGAs) for 17 (no polyploid species) and 24 (including 7 polyploid species) species using cactus (v. 2.1.1), respectively [42]. The WGAs were utilized to construct a phylogenetic tree with *Beaufortia kweichowensis* as the root. To facilitate the analysis, syntenic blocks were concatenated into 10-kb windows. Subsequently, a file containing 6 Mb (17 species) and 4.75 Mb (24 species) sequences for each genome was generated, respectively. To build the maximum likelihood tree, we employed RAxML (v. 8.2.12) [43] with the following parameters: -p 12345 -# 100 -m GTRGAMMA -s all.phylip -o B.kweichowensis -f a -x 12345 -k -n tree -T 10. The coalescent species tree estimations were performed using Astral (v. 5.15.5) [44]. For estimating divergence times, we used the MCMCTree in PAML (4.9j) [45] with four fossil calibration time points. The conserved scores of the 17 species were estimated using the phastCons tool from the phast packages [46]. For gene flow analysis, the ABBA-BABA test was conducted using the Dtrios program in Dsuite (0.4 r38) [47] software with the D-statistic method. The results were visualized using the Fbranch and dtools.py programs in Dsuite. To ensure a sufficient number of informative sites for analysis within each examined window, we employed a Python script named "ABBABABAwindows.py" to detect window D values. We used a window size of 20 kb with a step size of 10 kb, implemented through the script's parameters "-w 20000 -m 100 -s 10000".

RNA isolation and mRNA-seq

Total RNA from the brain, liver, intestine, muscle, kidney, and testis organs of three individuals (*C. alburnus* and *M. amblycephala*) was isolated and purified according to TRIzol extraction method, respectively [48]. The RNA concentration was measured using NanoDrop technology. Total RNA

samples were treated with DNase I (Invitrogen) to remove any contaminating genomic DNA. The purified RNA was quantified using a 2100 Bioanalyzer system (Agilent, Santa Clara, CA, USA). The isolated mRNA was fragmented with a fragmentation buffer. The resulting short fragments were reverse transcribed and amplified to produce cDNA. The transcriptome data of 36 samples (three biological replicates) was obtained using DNA nanoball (DNBSEQ-T7) technology according to the standard method [49]. The main steps were listed below: 1) single-stranded circular libraries were prepared using MGI Library Prep Kits; 2) after the hybridization of a DNA anchor, a fluorescent probe is attached to the DNA nanoball using combinatorial probe anchor sequencing chemistry; 3) the high-resolution imaging system captures the fluorescent signal; 4) after digital processing of the optical signal, the sequencer generates high-quality and accurate sequencing information. Low-quality bases and adapters were trimmed out using SOAPnuke with the thresholds "-n 0.01 -l 20 -q 0.4 -A 0.25 --cutAdaptor -Q 2 -G --polyX 50 --minLen 150" [50]. The high-quality reads were used in the next analyses.

Gene expression profiling

All clean reads of *M. amblycephala* and *C. alburnus* were mapped to their corresponding reference genomes using HISAT2 (v. 2.1.0) [36] with default parameters. Then, the mapped files were handled with SAMtools (v. 1.10) [51], while the unique mapped reads were obtained using htseq-count (v. 0.12.4) [52]. The gene expression value of mRNA-seq was normalized and calculated based on the transcripts per million (TPM) values. Genes with mapped reads < 5 in each sample were not used in our next analyses. Differential expression (DE) analysis was performed using Deseq2 [53] of R package with the thresholds: p-value < 0.001 and Padj < 0.001. Organ-specific genes (OSGs) were identified using the following criteria: Gene expression in the target tissue or organ differs significantly from that in the other five tissues and organs. Orphan genes (OGs) are detected based on the thresholds of BLASTx with an e-value of $1e^{-5}$ and tBLASTx with an e-value of $1e^{-5}$. The

sequences with no BLAST result in the public database were considered potential OGs. Then, the expressed OGs (TPM > 10) were considered OGs in the corresponding tissue or organ. GO analysis was performed with a significance threshold (false discovery rate of Benjamini–Hochberg method < 0.05).

Diversifying selection analysis

17,337 orthologous gene pairs (OGPs) between *M. amblycephala* and *C. alburnus* were obtained using the all-against-all reciprocal BLASTP (v. 2.8.1) with an e-value of $1e^{-6}$ based on protein sequences (sequence alignment > 70%). Then, transcripts that were shorter than 300 bp were discarded from OGPs. OGPs in the comparison of *M. amblycephala* and zebrafish and the comparison of *M. amblycephala* and zebrafish were obtained based on the above thresholds. We performed DE analyses on the OGPs between *M. amblycephala* and *C. alburnus* in the six tissues and organs. Differential expression (DE) analysis was performed using Deseq2 [53] with the thresholds: *p*-value < 0.001 and Padj < 0.001. The Ks and Ka/Ks values were calculated based on the below analysis process: 1) ParaAT2.0 and muscle software were used in sequence alignment of OGPs with the default parameters; 2) kaks_calculator3.0 program was used to calculate Ks and Ka/Ks values using maximum likelihood method [54]. The threshold of a *p*-value < 0.05 was used in our analyses.

Measurement of enzymatic content

Equal amounts of liver (0.1 g) from *M. amblycephala* and *C. alburnus* (10 individuals in each species) were collected from Engineering Center of Polyploid Fish Breeding of National Education Ministry in Hunan, China. Then, homogenates were used to determine the activity of trypsin and lipase. Trypsin assay kit (A080-2-2) and lipase assay kit (A054-1-1) were purchased from Nanjing Jiancheng Bioengineering Institute (Jiangsu, China), and the experimental protocols followed the

manufacturer’s instructions. The significant difference was performed using Student's *t*-test.

Hematoxylin and eosin staining

A 10 mm-thick section of skeletal muscle from four fish species, *M. amblycephala*, *C. alburnus*, *C. idella*, and *E. bambusa*, was dissected from the dorsum region. A 10 mm-thick section of intestine was dissected from the abdominal cavity of each of the four fish species after removing food residues. The tissue sections were then fixed in Bouin's solution for 24 hours. After fixation, the tissues were washed with distilled water for 4 hours at room temperature. The fixed tissues were then dehydrated using a series of alcohol concentrations (e.g., 70%, 80%, 90%, and 100%) and embedded in paraffin blocks. The paraffin-embedded tissue blocks were sectioned into 10 μm-thick slices using a microtome. The tissue sections were processed for hematoxylin and eosin (HE) staining according to the manufacturer's instructions using an HE staining kit. Digital images of the stained sections were captured using a microscope (DX8; Olympus, Tokyo, Japan). The samples obtained from three individuals were performed for each hybrid variety, and quantitative data on HE staining were collected from them.

Results

Genome assembly

A total of eight species of cyprinid fishes from East Asia were sequenced using PacBio HiFi or Oxford Nanopore technology, resulting in over 602.21 Gb of raw data. *De novo* assembled genomes were obtained with contig N50 ranging from 7.57 Mb to 38.12 Mb. For blunt snout bream *(Megalobrama amblycephala*, BSB) and topmouth culter (*Culter alburnus*, TC) two chromosome-scale genomes were generated using 204.8 Gb Hi-C data. These genomes exhibited scaffold N50 values of 42.91 Mb and 39.60 Mb, respectively (Table 1 and Supplemental File 1: Tables S1-S2). The quality of these assemblies was evaluated using BUSCO, with scores ranging

from 94.5% to 98.7% (Supplemental File 1: Table S3). We significantly improved the genome assembly data for both *M. amblycephala* (contig N50 length increased from 2.4 Mb to 15.42 Mb [55]) and *C. alburnus* (contig N50 length enhanced to 18.55 Mb, surpassing the previous 17.8 Mb [56]). We are unveiling high-quality genome data for the first time for *Cirrhinus molitorella*, *Pseudorasbora parva*, and *Xenocypris davidi*. Through the integration of *de novo*, protein homology, and cDNA-based prediction, a total of 26,550 and 27,303 protein-coding genes were annotated for *M. amblycephala* and *C. alburnus*, respectively (Supplemental File 1: Tables S4-S5). Repetitive elements accounted for 568.18 Mb (51.81% of the genome assembly) and 544.26 Mb (50.49% of the genome assembly) in *M. amblycephala* and *C. alburnus*, respectively (Supplemental File 1: Table S6). Non-coding RNA was predicted in *M. amblycephala* (8.76% of the genome assembly) and *C. alburnus* (7.21% of the genome assembly), respectively (Supplemental File 1: Table S7). Furthermore, we obtained high-quality assembled genome sequences for 15 cyprinid fishes (with an average scaffold N50 of 33.37 Mb) and *Beaufortia kweichowensis* from public databases (Supplemental File 1: Table S8). These 23 cyprinid fishes represent seven subfamilies of non-polyploid cyprinid fish (*Danioninae*, *Xenocyprinae*, *Gobioninae*, *Leuciscinae*, *Cultrinae*, *Labeoninae*, and *Hypophthalmichthyinae*) and three subfamilies of polyploid cyprinid fish (*Schizothoracinae*, *Barbinae*, and *Cyprininae*) (Table 1, Supplemental File 1: Tables S1-S2, and S8). Their genome sizes varied from 0.86 Gb to 1.90 Gb, with corresponding chromosome numbers spanning a wide range of 48 to 150 (Table 1, Supplemental File 1: Tables S1-S2, and S8).

Table 1. Assembly statistics of eight species of cyprinid fishes

Species	Common name	Subfamily	Scaffold N50 (Mb)	Contig N50 (Mb)	Contig length (Mb)	BUSCO Completeness (%)
Megalobrama amblycephala	Blunt snout bream	*Cultrinae*	42.91	15.42	1096.68	94.5%
Culter alburnus	Topmouth culter	*Cultrinae*	39.60	18.55	1077.98	98.3%
Ctenopharyngodon	Grass carp	*Leuciscinae*	/	35.62	901.49	98.5%

idella						
Cirrhinus molitorella	Mud carp	*Labeoninae*	/	38.12	1066.79	98.6%
Pseudorasbora parva	Stone moroko	*Gobioninae*	/	7.57	1292.12	98.5%
Xenocypris davidi	Bleeker's yellow tail	*Xenocyprinae*	/	38.11	1044.52	98.7%
Gobiocypris rarus	Raregudgeon	*Danioninae*	/	13.24	1108.08	98.3%
Elopichthys bambusa	Yellow cheek carp	*Leuciscinae*	/	30.38	863.54	98.4%

Phylogenomic analyses and introgression

To investigate the evolutionary relationships among extant cyprinids, we analyzed 24 genomes with butterfly hillstream loach (*B. kweichowensis*) as an outgroup (Fig. 1A and Supplemental File 2: Fig. S1). Our results showed that *Gobiocypris rarus* belongs to the subfamily *Gobioninae,* even though from a morphological perspective, it appears similar to zebrafish (which belongs to the subfamily *Danioninae* of Cyprinidae) [57]. Molecular clock analysis with fossil calibration indicated their divergence time ranging from 41.5-61.3 million years age (MYA) (Fig. 1A and Supplemental File 1: Table S9). Phylogenetic trees reconstructed the evolutionary history of subfamily *Leuciscinae*, showing that one group (including *Leuciscus idus, Abramis brama,* and *Rutilus rutilus*) diverged from another group (*Ctenopharyngodon idella* and *Elopichthys bambusa*) ranging from 23.8-35.1 MYA. This divergence occurred earlier than the divergence times observed among other subfamilies (*Cultrinae*, *Gobioninae, Xenocyprinae,* and *Hypophthalmichthyinae*) (Fig. 1A and Supplemental File 1: Table S9). Furthermore, phylogenomics analysis provided key evidence regarding the divergence of common ancestors of extant cyprinids, which ranged from 81.9-100.0 MYA (Fig. 1A and Supplemental File 1: Table S9). The ancestor of *Danio rerio* (subfamily: *Danioninae*) diverged early in the evolution of extant cyprinids, while the ancestor of *Cirrhinus molitorella* and *Labeo rohita* (subfamily: *Labeoninae*) diverged between 36.5-53.6 MYA (Fig. 1A and Supplemental File 1: Table S9). These findings will challenge the traditional understanding of cyprinid fish classification based on morphological characteristics. They will assist us in understanding the evolutionary process of

fish and constructing more reasonable classification relationships within the cyprinids, with the support of data from fields such as fossils, monsoons, and geography [58, 59].

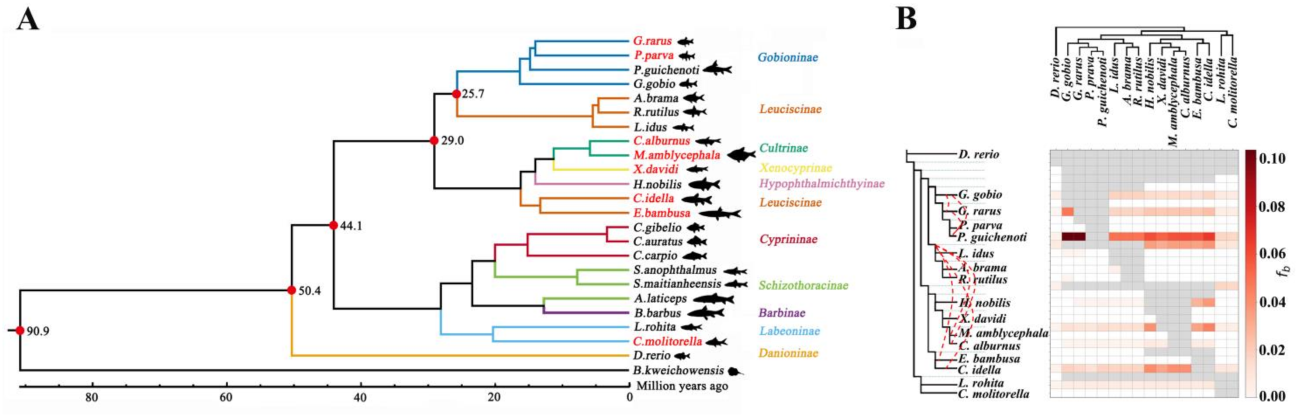

Figure 1: Phylogenomic analyses of cyprinid fish

(A) Time-calibrated phylogenetic tree. Red pot represents fossil calibration time points, which were obtained from “Timetree of Life” (http://timetree.org/). Black number in the branch represents the median of the range of divergence times. The name marked in red represents the species sequenced in this study. (B) Gene flow determined using the f-branch method. Dashed red line represents gene flow (f4-ratio > 0.05) between two species.

Previous studies have reported phylogenetic discordance across genome regions in non-polyploid cyprinid fishes from the East Asian region. This discordance has been attributed to incomplete lineage sorting, introgression, and the fish's demographic history [60]. To investigate this, we conducted gene flow analysis and observed pervasive introgression among these species (f4-ratio > 0.0006, Z-score > 3, and *p*-value < 0.05) (Fig. 1B). The ABBA-BABA tests [47, 61] revealed strong gene flow events among subfamily *Gobioninae*, including *Paracanthobrama guichenoti* and *Gobiocypris rarus* (f4-ratio = 0.1, Z-score = 129 and *p*-value < 0.001), *G. rarus* and *Gobio gobio* (f4-ratio = 0.1, Z-score = 120 and *p*-value < 0.001), and *P. guichenoti* and *G. gobio* (f4-ratio = 0.1, Z-score = 96 and *p*-value < 0.001) (Fig. 1B and Supplemental File 1: Table S10). Notably, distinct

gene flow signals (f4-ratio > 0.05) between two species were predominantly detected in 22 groups involving 12 species and 7 subfamilies (*Cultrinae*, *Danioninae*, *Gobioninae*, *Hypophthalmichthyinae*, *Leuciscinae_1*, *Leuciscinae_2*, and *Xenocyprinae*) (red dotted line in Fig. 1B and Supplemental File 1: Table S10). A recent study involving mitochondrial genomes and *de novo* nuclear genomes has shown frequent gene flow events in the radiation of cyprinid fishes [59]. Therefore, our results based on high-quality genome assemblies suggest that gene flow is the primary factor contributing to the observed phylogenetic discordance among non-polyploid cyprinid fishes.

Conservation of the reproductive system in speciation

The frequent occurrence of gene flow events suggests incomplete reproductive isolation (RI) as a contributing factor to cyprinid fish speciation in the East Asian region. To investigate the underlying reasons, our study primarily focuses on exploring genetic variations related to reproductive function. *M. amblycephala* and *C. alburnus* belong to different genera within the subfamily *Cultrinae*, while their habitats partly overlap in the middle and lower reaches of the Yangtze River Basin (Fig. 2A). Although laboratory experiments suggest some degree of postzygotic isolation, no evidence of natural hybrid populations exists for these species in the wild [62]. A conserved synteny analysis revealed a high degree of conservation in their most homologous genes (Supplemental File 2: Fig. S2-S3). Therefore, conducting a comparative analysis between these two species provides an effective model for our study.

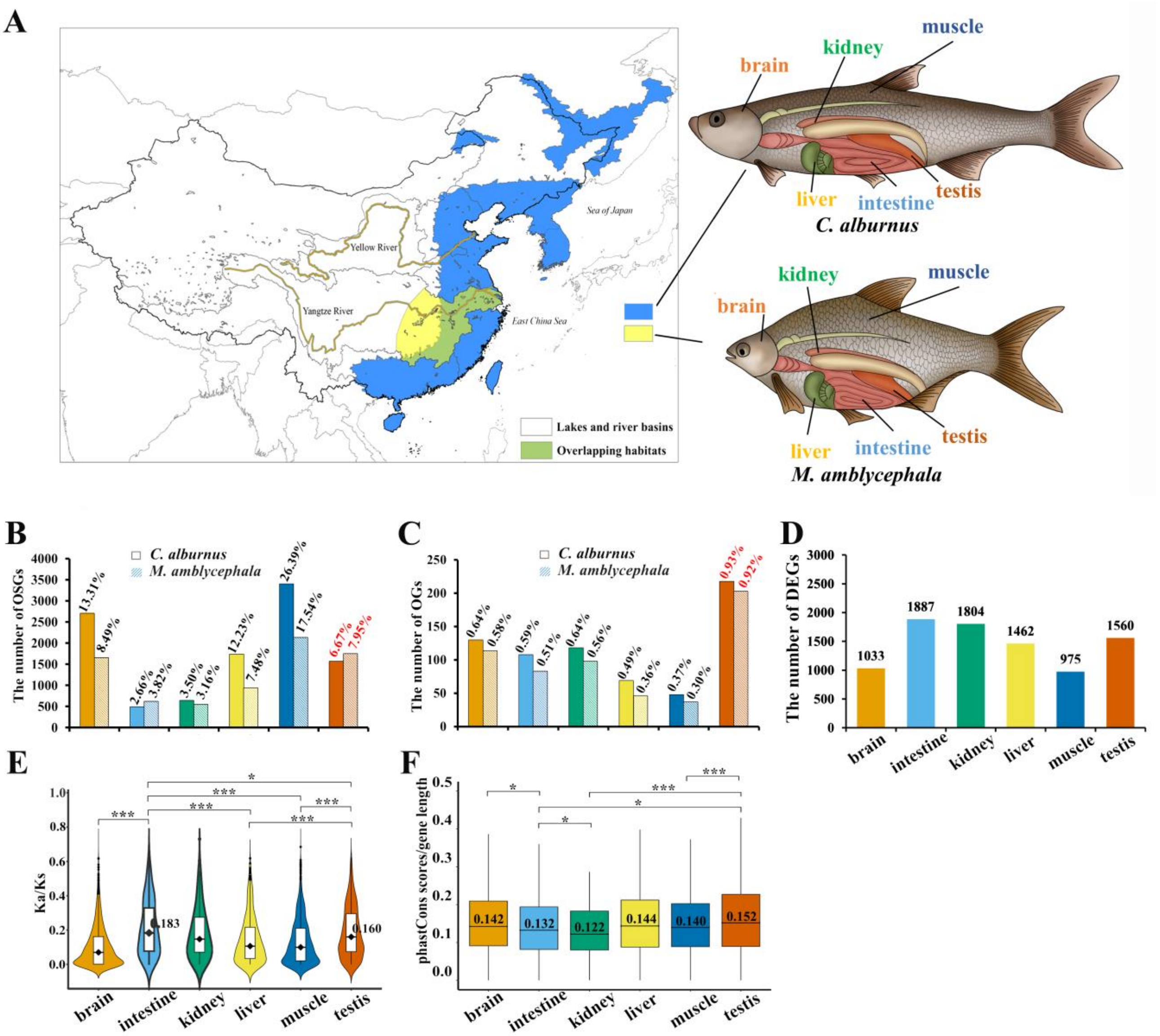

Figure 2: Divergent evolution of *M. amblycephala* and *C. alburnus* in East Asia

(A) Habitat distribution of extant *M. amblycephala* [63] and *C. alburnus* (https://www.iucnredlist.org/species/159755674/159755678). Partly habitat overlaps between the two species. (B) The number and percentage of OSGs in *M. amblycephala* and *C. alburnus*. (C) The number and percentage of OGs in *M. amblycephala* and *C. alburnus*. (D) Differential expression between *M. amblycephala* and *C. alburnus* in six tissues and organs. (E) The distribution of Ka/Ks values relating to OSGs in the six tissues and organs. The median value (black dot) was signed in figure. (F) Conserved scores of OSGs in the six tissues and organs. The median value (black line and number) was signed in figure. The comparisons involve the intestine and testis. "*" represents "0.01

$< p$-value ≤ 0.05", "**" represents "$0.001 < p$-value ≤ 0.01", and "***" represents "p-value ≤ 0.001".

To investigate the genetic differences between these two species, we analyzed the gene expression profiles of six different tissues and organs (brain, liver, intestine, muscle, kidney, and testis) and conducted organ-specific gene (OSG) analysis in both species (Supplemental File 1: Tables S11-S12). Our findings revealed that the testis exhibited a higher proportion of OSGs, accounting for 6.67% in *C. alburnus* and 7.95% in *M. amblycephala*, compared to the intestine and kidney (Fig. 2B). Furthermore, we focused on genetic variations in orphan genes (OGs), which are genes with no homologs in other evolutionary lineages and are found in all genomes [64]. We identified 649 OGs in *C. alburnus* and 655 OGs in *M. amblycephala*, with the testis having the highest number of OGs (0.93% in *C. alburnus* and 0.92% in *M. amblycephala*) among all other tissues and organs (Fig. 2C and Supplemental File 1: Table S13). These results suggest a rapid divergence, specifically in the testis, in comparison to other tissues or organs. However, the number of differentially expressed genes (DEGs) between the two species was fewer in the testis when compared to the intestine, liver, and kidney (Fig. 2D, Supplemental File 2: Fig. S4 and Supplemental File 1: Tables S14-S16). Moreover, to gain insights into functional divergence between *M. amblycephala* and *C. alburnus*, we calculated the Ka/Ks values of OSGs between the two species and found that these values in the testis were lower than those in the intestine (t-test: $p = 0.04$), albeit higher than those in the liver, brain, and muscle (t-test: $p < 0.001$) (Fig. 2E). The results regarding DEGs and the shared genes (OSGs and DEGs) also indicated that the Ka/Ks values in the testis were not the highest among the five tissues and organs (Supplemental File 2: Fig. S5). Finally, to assess the degree of sequence conservation [46], we calculated phastCons scores of OSGs and observed the highest median value in the testis, which was higher than in the intestine, kidney, and muscle (t-test: $p < 0.05$) (Fig. 2F). A similar phenomenon was noted when analyzing DEGs using phastCons scores (Supplemental File 2: Fig. S6). *M. amblycephala* and *C. alburnus* exhibited lower genetic variation

in their testes compared to their intestines.

Rapid evolution of digestive system in speciation

The diversity and adaptability of the fish digestive system enable them to make appropriate adjustments to various types of food, such as plankton, aquatic plants, and benthic organisms. This adaptability further contributes to diet-driven ecological niche differentiation [65]. Differential expression analyses exhibited that the lowest number of OSGs in *C. alburnus* was detected in the intestine (752, 4.15%), while the second lowest OSGs in *M. amblycephala* were found in the intestine (942, 5.95%) (Fig. 2B). The number of OGs in the intestine (0.59% in *C. alburnus* and 0.51% in *M. amblycephala*) was lower compared to the testis, brain, and kidney (Fig. 2C). The study demonstrates that the genetic makeup of the intestine exhibits a higher degree of conservation compared to the other tissues and organs. However, a rapid genetic divergence between the herbivorous *M. amblycephala* and the carnivorous *C. alburnus* was observed in their intestines. For instance, the highest number of DEGs between the two species was detected in their intestines (Fig. 2D and Supplemental File 1: Tables S14-S15). Moreover, the Ka/Ks values of OSGs were higher in the intestine compared to the brain, kidney, muscle, and testis (Fig. 2E). Lastly, the phastCons scores of OSGs in the intestine were lower than those in the brain, liver, muscle, and testis, although they were higher than in the kidney (Fig. 2F). Similar trends were observed in the phastCons scores of DEGs (Supplemental File 2: Fig. S6). These results suggest that rapid genetic diversity between *M. amblycephala* and *C. alburnus* occurs in the digestive organs.

To investigate the genetic basis of diet divergence between herbivorous *M. amblycephala* and carnivorous *C. alburnus*, we conducted a functional analysis of their DEGs in the intestine. These genes associated with digestive enzymes were enriched for hydrolyzing O-glycosyl compounds (GO: 0004553) and peptidase activity (GO: 0008233) in terms of Molecular Function annotation, while carbohydrate metabolic process (GO: 0005975) and lipid catabolic process (GO: 0016042) were

enriched for Biological Process annotation (Supplemental File 2: Fig. S7). Among these genes, *dpp2*, *ctrl*, *psb7*, and *ppce* were identified as key genes involved in peptidase activity, exhibiting higher expression in the digestive organs (liver and intestine) of *C. alburnus* compared to *M. amblycephala* (Fig. 3A). After detecting the enzyme activities of trypsin and lipase in digestive organs, we found that the enzyme activities were higher in the carnivorous *C. alburnus* compared to the herbivorous *M. amblycephala* (Fig. 3B). We conducted analyses on positively selected genes (PSGs) (Ka/Ks > 1) between the two species and identified 30 of them that belong to OSGs in the six tissues and organs (Supplemental File 1: Table S17). Among the share genes of PSGs and OSGs in the intestine, *caspbl* and *vsig* were found to be associated with peptidase activity (GO: 0008233), apoptosis, and immune responses, which are closely related to the types of digested food (Supplemental File 2: Fig. S8) [66, 67]. These results reflect the adaptive evolution of their diets in terms of digestive enzyme secretion.

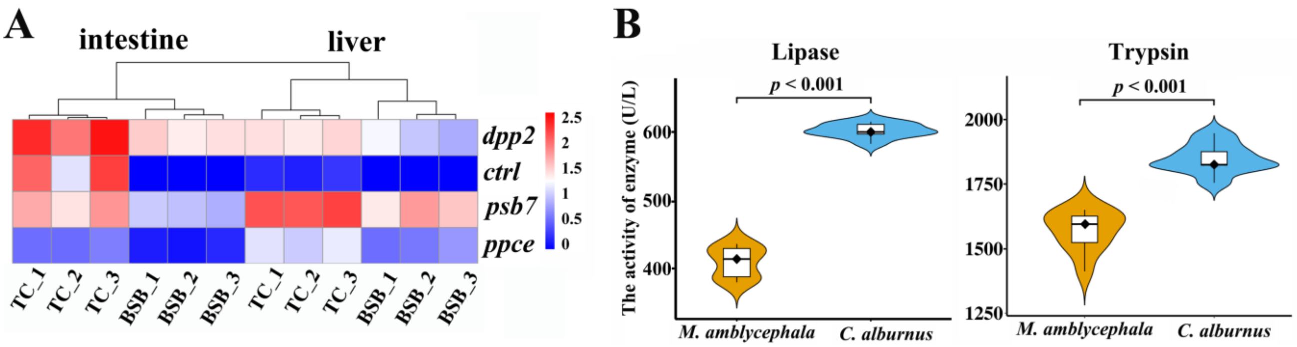

Figure 3: Diet divergences between *M. amblycephala* and *C. alburnus*

(A) The four genes relating to differential expression between *M. amblycephala* and *C. alburnus* in both the intestine and liver (three biological replicates showed “_1”, “_2”, and “_3”). (B) Significant differences in the enzyme activity of lipase and trypsin for the comparison between *M. amblycephala* and *C. alburnus*.

Gene flow and its impact on feeding habits

Frequent gene flow events were observed among cyprinid fishes, including *M. amblycephala*, *C.*

alburnus, *C. idella*, and *E. bambusa*. Significant gene flow events were detected between carnivorous *C. alburnus* (subfamily *Cultrinae*) and *E. bambusa* (subfamily *Leuciscinae*) (Z-score > 45.8, f4–ratio = 0.039, and *p*-value < 0.001). Additionally, gene flow events were identified between the herbivorous *M. amblycephala* (subfamily Cultrinae) and *C. idella* (subfamily *Leuciscinae*) (Z-score > 38.3, f4–ratio = 0.044, and *p*-value < 0.001) (Fig. 1B and Supplemental File 1: Table S10). The overlapping habitats of these four species were primarily distributed in the eastern region of China (Fig. 4A).

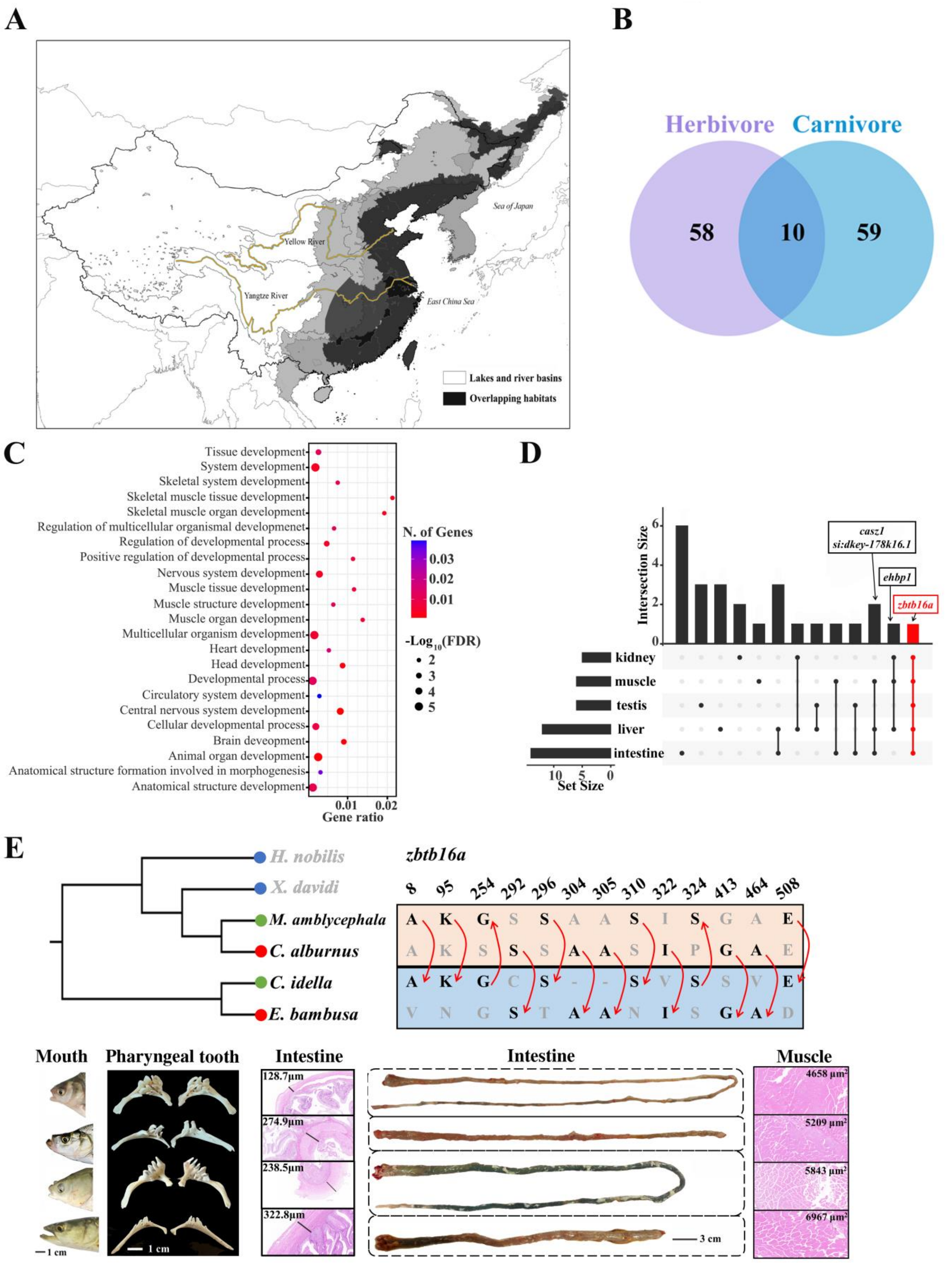

Figure 4: Gene flow in herbivorous and carnivorous fishes

(A) Habitat distribution of *M. amblycephala* [63], *C. alburnus*, *C. idella* (https://www.iucnredlist.org/species/61295/3102796), and *E. bambusa* (https://www.iucnredlist.org/species/166188/159755690) in East Asia. Their overlap and unique habitats reflect their speciation of endemic East Asian cyprinid fishes in the river-lake ecosystems of East Asia. (B) Venn diagram showing introgressed genes between carnivores and herbivores. (C) The introgressed genes were expressed in different tissues and organs, while there was no introgressed gene expressed in the brain. (D) GO analyses of the shared 10 introgressed genes in carnivores and herbivores. (E) Phylogenomic analyses of the six endemic cyprinid species in China, introgressions visualizing at SNPs of *zbtb16a* (potential introgressed SNPs marking red arrow), the morphologies of mouth, pharyngeal teeth, and intestine, microstructure of intestine (thickness of intestinal wall marking in figure) and muscle (average area marking in figure) in the four endemic cyprinid species. Blue dot represents filter-feeding fish, green dot represents herbivorous fish, and red dot represents carnivorous fish.

To investigate the effects of gene flow events on diet diversity, we analyzed the 117 introgressed genomic regions (window size: 20 kb) between the two carnivorous fish species, which were associated with 69 genes (Supplemental File 1: Table S18). Similarly, the 102 introgressed regions (window size: 20 kb) between the two herbivorous fish species were associated with 68 genes (Supplemental File 1: Table S19). Among these genes, the top three molecular function annotations in both carnivores and herbivores were related to transcription regulator activity (GO: 0140110), DNA-binding transcription factor activity (GO: 0003700), and RNA polymerase II-specific activity (GO: 0000981) (Supplemental File 1: Tables S20-S21). Among these genes, 10 introgressed genes were shared between carnivores and herbivores (Fig. 4B). Moreover, 84 categories (38.36%) for biological processes and 16 categories (66.67%) for molecular functions were shared between the two dietary groups (FDR < 0.05, Fig. 4C and Supplemental File 2: Fig.

S9-S10). These shared introgressed genes were associated with animal organ development, including skeletal muscle organ development (GO: 0060538; *sox6* and *ttn.2*) and head development (GO: 0060322; *zfhx3*, *tcf7l2*, and *meis1b*) (Supplemental File 2: Fig. S11). Among all the introgressed genes, only *zbtb16a* was expressed in the five tissues and organs (muscle, liver, intestine, testis, and kidney), while no introgressed gene was expressed in the brain (Fig. 4D).

There were potential relationships between diet habits and organ development, including mouth and pharyngeal tooth morphologies, intestinal morphology, and skeletal muscle structure (Fig. 4E). Comparative analyses revealed that carnivorous fishes exhibited larger and superior mouths, longer and sharper teeth, shorter intestines, thinner intestine linings, and smaller cross-sectional areas in skeletal muscle fibers compared to herbivorous fishes (Fig. 4E). Diversified introgression events between the subfamilies *Cultrinae* and *Leuciscinae* were observed in *zbtb16a*, a gene associated with osteogenic differentiation [68]. It is noteworthy that some of the introgressed genes have been experimentally validated in zebrafish to have functional associations with dietary traits. For instance, *tp53* and *tle3a* are implicated in intestinal morphology [69, 70], while *grin2bb* and *grin1a* are linked to food intake behavior [71, 72]. Additionally, *znf536*, *zfhx3*, *elavl4*, *hoxc11a*, *pik3r3b*, and *irf2bpl* have been identified as potential regulators of swimming behavior. These findings suggest that introgressed genes related to organ development and feeding behavior may contribute to diet divergence for these fishes.

Discussion

The East Asian region, characterized by its unique topography, including the uplift of the Qinghai-Tibet Plateau, abundant rivers, and diverse climatic environments, is home to a multitude of freshwater fish species [59, 73, 74]. Among these fishes, cyprinids comprise the largest and most diverse group of vertebrate animals, with over 654 species documented, including 440 endemic species in China [75]. Understanding the genetic mechanisms behind their rapid speciation is crucial

to unraveling the process of evolutionary radiation in East Asian cyprinids. Our findings suggest that frequent gene flow events among cyprinid fishes have contributed to their rapid adaptive radiation, as evidenced by our analyses of seven non-polypoid subfamilies. The question arises: What is the relationship between introgressive hybridization in East Asian cyprinids and rapid speciation?

The rapid evolutionary changes in the mammalian testis are reflected at the molecular level, and this is an important factor contributing to reproductive isolation. Previous studies comparing gene expression across different mammalian organs have shown that the testis exhibits the highest rates of evolutionary expression change [76, 77]. Therefore, we examined the genetic divergence of different organs in the comparison between *M. amblycephala* and *C. alburnus* (subfamily *Cultrinae*), and found lower divergence in the testis compared to the intestine. Furthermore, in laboratory experiments involving intergeneric and interspecific hybrids among extant cyprinid fishes, fertile progeny of both sexes were frequently obtained [13, 62]. These findings suggest the presence of incomplete RI and help elucidate the reasons behind the frequent occurrence of introgressive hybridization in East Asian cyprinids. But what factors are hindering speciation and driving rapid speciation in East Asian cyprinids?

The complex and variable inland water ecosystem plays a crucial role in the adaptive evolution of fish [78, 79]. Among these factors, the diversity of food sources gradually influences the feeding habits of different populations, resulting in the adaptive evolution of their digestive and locomotion systems [80]. Our findings suggest that speciation in *M. amblycephala* and *C. alburnus* may have been driven by diet-dependent adaptations. Cyprinid fishes display significant diversity in behavior, habitat, geography, and morphology, including variations in feeding and digestive organs [59, 75]. The presence of robust pharyngeal teeth and toothless jaws enables them to consume a wide range of foods [81, 82]. Our results reveal that the rapid genetic divergence between *M. amblycephala* and *C. alburnus* occurs in the intestine. The variations in digestive enzyme secretion and digestive organs reflect their distinct feeding preferences and the effectiveness with which they metabolize different

food types [83]. Considering the absence of post-zygotic isolation and the overlapping habitat between *M. amblycephala* and *C. alburnus* [62, 84], our results suggest that ecological differentiation driven by dietary differences may be an important factor leading to the rapid formation of these two species.

When post-zygotic reproductive isolation is no longer a significant barrier to gene flow among East Asian cyprinids, natural selection, including monsoon activities [59], and the uplift of the Qinghai-Tibet Plateau [74], can attenuate pre-zygotic isolation, providing opportunities for gene flow, thus promoting speciation in cyprinid fishes [85]. To adapt to diverse food supplies in different aquatic environments, feeding habits have diverged in the subfamilies *Cultrinae* (herbivorous *M. amblycephala* and carnivorous *C. alburnus*) and *Leuciscinae* (herbivorous *C. idella* and carnivorous *E. bambusa*). Does gene flow facilitate the divergence of diets for adaptive evolution? Our results demonstrate the introgression of genes associated with skeletal muscle and head development between fishes with the same diet. These changes play crucial roles in feeding and digestive efficiency. Fishes with the same diet in different subfamilies exhibit similar phenotypes involving the mouth, teeth, intestine, and muscle. These results suggest that coevolving interactions of diet habits occur in their speciation through introgressive hybridization. However, further evidence is needed to establish a definitive association between dietary convergent evolution and gene flow.

Additional Files

Supplemental File 1. Table S1. Summary of whole genome sequencing in eight species. Table S2. Genome assembly of eight species. Table S3. Completeness of the eight assembled genomes. Table S4. Statistics of gene prediction. Table S5. Gene function annotation of *M. amblycephala* and *C. alburnus*. Table S6. Summary of repeat contents. Table S7. The summary of predicted non-coding RNA in *M. amblycephala* and *C. alburnus*. Table S8. Information of downloaded genomes. Table S9. Range of divergence time in Fig. 1. Table S10. D statistic on the species tree based on genome-wide single nucleotide polymorphisms (SNPs). The outgroup was fixed as *Beaufortia kweichowensis*. Table S11. Summary of transcriptome sequencing data. Table S12. Summary of transcriptome mapping data. Table S13. List of orphan genes (OGs) in *M. amblycephala* and *C. alburnus*. Table S14. Summary of differential expressed genes (DEGs) between *M. amblycephala* and *C. alburnus* in six organs. Table S15. The gene number of orthologous gene pairs and differential expressed genes (DEGs) in six organs. Table S16. The summary of organ-specific genes (OSGs), positive selective genes (PSGs), and differential expressed genes (DEGs) in six organs. Table S17. Summary of expressed positive selective genes (PSGs) between *C. alburnus* (TC) and *M. amblycephala* (BSB) in the six organs. Table S18. Summary of gene flow between carnivorous *C. alburnus* and *E. bambusa*. Table S19. Summary of gene flow between herbivorous *M. amblycephala* and *C. idella*. Table S20. GO enrichment of introgressed genes between carnivorous *C. alburnus* and *E. bambusa*. Table S21. GO enrichment of introgressed genes between herbivorous *M. amblycephala* and *C. idella*.

Supplemental File 2. Fig. S1. Phylogenetic trees constructed using multiple whole-genome alignments of 17 (no polyploid species) and 24 (including 7 polyploid species) species with *Beaufortia kweichowensis* as the root, respectively. (A) Concatenation-based method for estimating a phylogenetic tree of 17 species with 10-kb length windows. (B) Coalescent method for estimating a phylogenetic tree of 17 species with 10-kb length windows. (C) Concatenation-based method for estimating a phylogenetic tree of 24 species with 10-kb length windows. (D) Coalescent method for

estimating a phylogenetic tree of 24 species with 10-kb length windows. (E) Species tree with estimated divergence time. Fig. S2. The Hi-C interaction heatmap of 24 linkage groups in the genomes of *M. amblycephala* and *C. alburnus*. Fig. S3. The collinearity analysis between *M. amblycephala* and *C. alburnus*. Twenty-four pairs of homologous chromosomes were determined based on 17,337 orthologous gene pairs. Fig. S4. The differential expression between *M. amblycephala* and *C. alburnus* in six organs. Up-regulated genes in *M. amblycephala* are marked in blue, while the up-regulated genes in *C. alburnus* are marked in red. Fig. S5. The distribution of Ka/Ks values in the six organs. (A) The Ka/Ks values of DEGs. (B) The Ka/Ks values of share genes between DEGs and OSGs. The median value is indicated by a black dot, and the gene number is provided below each organ name. In the t-test, "*" represent $0.01 < \text{p-value} \leq 0.05$, "**" represents $0.001 < \text{p-value} \leq 0.01$, "***" represents $\text{p-value} \leq 0.001$. Fig. S6. Conserved scores of DEGs (*M. amblycephala* vs. *C. alburnus*) in the six organs. "*" represent $0.01 < \text{p-value} \leq 0.05$, "**" represents $0.001 < \text{p-value} \leq 0.01$, "***" represents $\text{p-value} \leq 0.001$. Fig. S7. The DEGs (*M. amblycephala* vs. *C. alburnus*) associated with diet habit. (A) The heatmap of the DEGs in the intestine. The hydrolyzing O-glycosyl compounds and peptidase activity in Molecular Function, as well as carbohydrate metabolic process and lipid catabolic process in Biological Process. (B) The gene distribution of DEGs in the intestine. Fig. S8. The alignment of two positively selected genes (PSGs) in intestine. Fig. S9. The GO terms of introgressed genes in the carnivorous (*C. alburnus* and *E. bambusa*) and herbivorous *(M. amblycephala* and *C. idella*) fishes. Fig. S10. The distribution of enriched functional categories (FDR < 0.05) in Biological Process and Molecular Function for the introgressed genes. Fig. S11. Heatmap exhibiting the expression of introgressed genes in the carnivorous (*C. alburnus* and *E. bambusa*) and herbivorous (*M. amblycephala* and *C. idella*) fishes.

Abbreviations

BSB: blunt snout bream; TC: topmouth culter; RI: Reproductive isolation; TRF: Tandem Repeats

Finder; TPM: transcripts per million; DE: Differential expression; OGs: Orphan genes; OGPs: orthologous gene pairs; MYA: million years age; OSG: organ-specific gene; PSGs: positively selected genes.

Acknowledgements

We thank Min Xie at Hunan Fisheries Science Institute for their invaluable assistance in collecting the fish samples.

Author contributions

S.J.L., L.R., and X.L.T. wrote the manuscript. S.J.L., D.D.W., L.R., and Q.Z.L. modified the manuscript and designed the study. X.L.T., L.R., and M.X.L. carried out bioinformatics analyses. J.L.C., X.G., H.Z., Y.K.T., Y.Y.Z., M.D.L., W.H.L., C.W., and J.W. extracted the raw material. All authors read and approved the final manuscript.

Funding

This research was supported by National Natural Science Foundation of China (32293252, 32341057, and U19A2040), Hunan Provincial Natural Science Foundation (2022JJ10035), Huxiang Young Talent Project of China (2021RC3093), National Key Research and Development Plan Program (2023YFD2401602), Laboratory of Lingnan Modern Agriculture Project (NT2021008), Special Funds for Construction of Innovative Provinces in Hunan Province (2021NK1010), earmarked fund for China Agriculture Research System (CARS-45), and 111 Project (D20007).

Competing Interests

The authors have declared that no competing interests exist.

Data Availability

Genomic sequencing data obtained from PacBio HiFi, Oxford Nanopore, and DNBSEQ-T7 technologies, as well as Hi-C data, have been submitted to the National Center for Biotechnology Information (NCBI) (accession numbers: SRR26190421-SRR26190427, SRR26139312-SRR26139313, SRR26139214-SRR26139215, and SRR26319599-SRR26319600). The assembled genome and annotation files of eight cyprinid fishes have been deposited on figshare (DOI: 10.6084/m9.figshare.24125487) and the National Genomics Data Center (NGDC) (accession numbers: GWHDOEU00000000, GWHDOEX00000000, GWHDOEV00000000, GWHDOEW00000000, GWHDOEB00000000, GWHDOEC00000000, GWHDOES00000000, and GWHDOET00000000). The raw reads of the mRNA-seq data have been submitted to NGDC (accession number: subCRA017373) and NCBI (accession numbers: SRR26087118-SRR26087153).

Animal ethics declarations

All procedures performed on animals were approved by the academic committee at Hunan Normal University, Hunan, China (approval number: 2020C034).

References

1. Froese R and Pauly D. FishBase. World Wide Web electronic publication. In: 2014.
2. Nelson J, Grande T and Wilson M. Fishes of the World, Fifth Edition. 2016.
3. Yang L, Sado T, Vincent Hirt M, Pasco-Viel E, Arunachalam M, Li JB, et al. Phylogeny and polyploidy: Resolving the classification of cyprinine fishes (Teleostei: Cypriniformes). Mol Phylogenet Evol. 2015;85:97-116. doi:doi: 10.1016/j.ympev.2015.01.014.
4. He FZ, Zarfl C, Bremerich V, David JN, Hogan Z, Kalinkat G, et al. The global decline of freshwater megafauna. Global Change Biol. 2019;25 11:3883-92.
5. Jacquemin SJ and Pyron M. A century of morphological variation in Cyprinidae fishes. BMC Ecology. 2016;16 1:48. doi:10.1186/s12898-016-0104-x.
6. German DP, Nagle BC, Villeda JM, Ruiz AM, Thomson AW, Contreras Balderas S, et al. Evolution of herbivory in a carnivorous clade of minnows (teleostei: cyprinidae): effects on gut size and digestive physiology. Physiological and biochemical zoology : PBZ. 2010;83 1:1-18. doi:10.1086/648510.
7. Yue PQ, Shan XH and Lin RD. Fauna sinica, osteichthyes, cypriniformes III. Science, Beijing (in Chinese). 2000.
8. Haenen O, Way K, Gorgoglione B, Ito T, Paley R, Bigarré L, et al. Novel viral infections threatening Cyprinid fish. Bulletin of the European Association of Fish Pathologists. 2016;36 1:11-23.
9. Brauer CJ, Sandoval-Castillo J, Gates K, Hammer MP, Unmack PJ, Bernatchez L, et al. Natural hybridization reduces vulnerability to climate change. Nat Clim Change. 2023;13:282–9. doi:10.1038/s41558-022-01585-1.
10. Mallet J. Hybridization as an invasion of the genome. Trends Ecol Evol. 2005;20 5:229-37. doi:10.1016/j.tree.2005.02.010.
11. Schumer M, Powell DL, Delclós PJ, Squire M, Cui R, Andolfatto P, et al. Assortative mating and persistent reproductive isolation in hybrids. Proc Natl Acad Sci U S A. 2017;114 41:10936-41. doi:doi:10.1073/pnas.1711238114.
12. Birkhead TR and Brillard JP. Reproductive isolation in birds: postcopulatory prezygotic barriers. Trends Ecol Evol. 2007;22 5:266-72. doi:10.1016/j.tree.2007.02.004.
13. Wang S, Tang CC, Tao M, Qin QB, Zhang C, Luo KK, et al. Establishment and application of distant hybridization technology in fish. Sci China Life Sci. 2019;62 1:22-45. doi:10.1007/s11427-018-9408-x.
14. Su GH, Logez M, Xu J, Tao SG, Villéger S and Brosse S. Human impacts on global freshwater fish biodiversity. Science. 2021;371 6531:835-8.
15. Dias MS, Oberdorff T, Hugueny B, Leprieur F, Jézéquel C, Cornu JF, et al. Global imprint of historical connectivity on freshwater fish biodiversity. Ecology Letters. 2014;17 9:1130-40.
16. Seehausen O. Hybridization and adaptive radiation. Trends Ecol Evol. 2004;19 4:198-207. doi:10.1016/j.tree.2004.01.003.
17. Geiger MF, Herder F, Monaghan MT, Almada V, Barbieri R, Bariche M, et al. Spatial heterogeneity in the Mediterranean Biodiversity Hotspot affects barcoding accuracy of its freshwater fishes. Mol Ecol Resour. 2014;14 6:1210-21.
18. Costedoat C, Pech N, Salducci M-D, Chappaz R and Gilles A. Evolution of mosaic hybrid zone between invasive and endemic species of Cyprinidae through space and time. Biological Journal of the Linnean Society. 2005;85 2:135-55.
19. Broughton RE, Vedala KC, Crowl TM and Ritterhouse LL. Current and historical hybridization with differential introgression among three species of cyprinid fishes (genus Cyprinella). Genetica. 2011;139:699-707.
20. Pereira CSA, Aboim MA, Ráb P and Collares-Pereira MJ. Introgressive hybridization as a promoter of genome

reshuffling in natural homoploid fish hybrids (Cyprinidae, Leuciscinae). Heredity. 2014;112 3:343-50. doi:10.1038/hdy.2013.110.

21. Aboim M, Mavárez J, Bernatchez L and Coelho M. Introgressive hybridization between two Iberian endemic cyprinid fish: a comparison between two independent hybrid zones. Journal of Evolutionary Biology. 2010;23 4:817-28.

22. Rønnestad I, Yufera M, Ueberschär B, Ribeiro L, Sæle Ø and Boglione C. Feeding behaviour and digestive physiology in larval fish: current knowledge, and gaps and bottlenecks in research. Reviews in Aquaculture. 2013;5:S59-S98.

23. Kuang ZR, Li F, Duan QJ, Tian CC, Nevo E and Li KX. Host diet shapes functionally differentiated gut microbiomes in sympatric speciation of blind mole rats in Upper Galilee, Israel. Front Microbiol. 2022;13:1062763. doi:10.3389/fmicb.2022.1062763.

24. Chen HY, Li CQ, Liu T, Chen SY and Xiao H. A Metagenomic Study of Intestinal Microbial Diversity in Relation to Feeding Habits of Surface and Cave-Dwelling Sinocyclocheilus Species. Microb Ecol. 2020;79 2:299-311. doi:10.1007/s00248-019-01409-4.

25. Sibbing F. Food capture and oral processing. Cyprinid fishes: systematics, biology and exploitation. 1991:377-412.

26. Hulsey CD, Machado-Schiaffino G, Keicher L, Ellis-Soto D, Henning F and Meyer A. The integrated genomic architecture and evolution of dental divergence in East African cichlid fishes (Haplochromis chilotes x H. nyererei). G3: Genes, Genomes, Genetics. 2017;7 9:3195-202.

27. Chen SF, Zhou YQ, Chen YR and Gu J. fastp: an ultra-fast all-in-one FASTQ preprocessor. Bioinformatics. 2018;34 17:i884-i90. doi:10.1093/bioinformatics/bty560.

28. J. H, Z. W, Z.Y. S, B.X. H, A.O. A, F. L, et al. An efficient error correction and accurate assembly tool for noisy long reads. bioRxiv. 2023:531669. doi:10.1101/2023.03.09.531669 %J bioRxiv.

29. Hu J, Fan JP, Sun ZY and Liu SL. NextPolish: a fast and efficient genome polishing tool for long-read assembly. Bioinformatics. 2020;36 7:2253-5. doi:10.1093/bioinformatics/btz891.

30. Cheng HY, Jarvis ED, Fedrigo O, Koepfli KP, Urban L, Gemmell NJ, et al. Haplotype-resolved assembly of diploid genomes without parental data. Nat Biotechnol. 2022;40 9:1332-5. doi:10.1038/s41587-022-01261-x.

31. Rao SSP, Huntley MH, Durand NC, Stamenova EK, Bochkov ID, Robinson JT, et al. A 3D Map of the Human Genome at Kilobase Resolution Reveals Principles of Chromatin Looping. Cell. 2014;159 7:1665-80. doi:10.1016/j.cell.2014.11.021.

32. Li H and Durbin R. Fast and accurate long-read alignment with Burrows-Wheeler transform. Bioinformatics. 2010;26 5:589-95. doi:10.1093/bioinformatics/btp698.

33. Servant N, Varoquaux N, Lajoie BR, Viara E, Chen CJ, Vert JP, et al. HiC-Pro: an optimized and flexible pipeline for Hi-C data processing. Genome Biol. 2015;16:259. doi:10.1186/s13059-015-0831-x.

34. Burton JN, Adey A, Patwardhan RP, Qiu R, Kitzman JO and Shendure J. Chromosome-scale scaffolding of de novo genome assemblies based on chromatin interactions. Nature biotechnology. 2013;31 12:1119-25. doi:10.1038/nbt.2727.

35. Stanke M, Diekhans M, Baertsch R and Haussler D. Using native and syntenically mapped cDNA alignments to improve de novo gene finding. Bioinformatics. 2008;24 5:637-44. doi:10.1093/bioinformatics/btn013.

36. Kim D, Paggi JM, Park C, Bennett C and Salzberg SL. Graph-based genome alignment and genotyping with HISAT2 and HISAT-genotype. Nat Biotechnol. 2019;37 8:907-15. doi:10.1038/s41587-019-0201-4.

37. Eddy SR. A probabilistic model of local sequence alignment that simplifies statistical significance estimation. PLoS Comput Biol. 2008;4 5:e1000069. doi:10.1371/journal.pcbi.1000069.

38. Ou SJ and Jiang N. LTR_FINDER_parallel: parallelization of LTR_FINDER enabling rapid identification of

long terminal repeat retrotransposons. Mob DNA. 2019;10:48. doi:10.1186/s13100-019-0193-0.
39. Ellinghaus D, Kurtz S and Willhoeft U. LTRharvest, an efficient and flexible software for de novo detection of LTR retrotransposons. Bmc Bioinformatics. 2008;9:18. doi:10.1186/1471-2105-9-18.
40. Ou SJ and Jiang N. LTR_retriever: A Highly Accurate and Sensitive Program for Identification of Long Terminal Repeat Retrotransposons. Plant Physiol. 2018;176 2:1410-22. doi:10.1104/pp.17.01310.
41. Chan PP, Lin BY, Mak AJ and Lowe TM. tRNAscan-SE 2.0: improved detection and functional classification of transfer RNA genes. Nucleic Acids Res. 2021;49 16:9077-96. doi:10.1093/nar/gkab688.
42. Armstrong J, Hickey G and Diekhans M. Progressive Cactus is a multiple-genome aligner for the thousand-genome era. Nature. 2020;587 7833:246-51. doi:10.1038/s41586-020-2871-y.
43. Höhler D, Pfeiffer W, Ioannidis V, Stockinger H and Stamatakis A. RAxML Grove: an empirical phylogenetic tree database. Bioinformatics. 2022;38 6:1741-2. doi:10.1093/bioinformatics/btab863.
44. Zhang C, Scornavacca C, Molloy EK and Mirarab S. ASTRAL-Pro: Quartet-Based Species-Tree Inference despite Paralogy. Mol Biol Evol. 2020;37 11:3292-307. doi:10.1093/molbev/msaa139.
45. Yang ZH. PAML 4: phylogenetic analysis by maximum likelihood. Mol Biol Evol. 2007;24 8:1586-91. doi:10.1093/molbev/msm088.
46. Cooper GM, Stone EA, Asimenos G, Green ED, Batzoglou S and Sidow A. Distribution and intensity of constraint in mammalian genomic sequence. Genome research. 2005;15 7:901-13. doi:10.1101/gr.3577405.
47. Malinsky M, Matschiner M and Svardal H. Dsuite - Fast D-statistics and related admixture evidence from VCF files. Mol Ecol Resour. 2021;21 2:584-95. doi:10.1111/1755-0998.13265.
48. Rio DC, Ares M, Hannon GJ and Nilsen TW. Purification of RNA using TRIzol (TRI reagent). Cold Spring Harb Protoc. 2010;2010 6:pdb.prot5439. doi:10.1101/pdb.prot5439.
49. Patterson J, Carpenter EJ, Zhu Z, An D, Liang X, Geng C, et al. Impact of sequencing depth and technology on de novo RNA-Seq assembly. BMC genomics. 2019;20 1:604. doi:10.1186/s12864-019-5965-x.
50. Chen YX, Chen YS, Shi CM, Huang ZB, Zhang Y, Li SK, et al. SOAPnuke: a MapReduce acceleration-supported software for integrated quality control and preprocessing of high-throughput sequencing data. GigaScience. 2017;7 1:1-6. doi:10.1093/gigascience/gix120.
51. Li H, Handsaker B, Wysoker A, Fennell T, Ruan J, Homer N, et al. The sequence alignment/map format and SAMtools. Bioinformatics. 2009;25 16:2078-9.
52. Srinivasan KA, Virdee SK and McArthur AG. Strandedness during cDNA synthesis, the stranded parameter in htseq-count and analysis of RNA-Seq data. Brief Funct Genomics. 2020;19 5-6:339-42. doi:10.1093/bfgp/elaa010.
53. Varet H, Brillet-Gueguen L, Coppee JY and Dillies MA. SARTools: A DESeq2- and EdgeR-Based R Pipeline for Comprehensive Differential Analysis of RNA-Seq Data. PLoS One. 2016;11 6:e0157022. doi:10.1371/journal.pone.0157022.
54. Zhang Z. KaKs_Calculator 3.0: Calculating Selective Pressure on Coding and Non-coding Sequences. Genomics Proteomics Bioinformatics. 2022; doi:10.1016/j.gpb.2021.12.002.
55. Liu H, Chen C, Lv M, Liu N, Hu Y, Zhang H, et al. A Chromosome-Level Assembly of Blunt Snout Bream (Megalobrama amblycephala) Genome Reveals an Expansion of Olfactory Receptor Genes in Freshwater Fish. Molecular Biology and Evolution. 2021;38 10:4238-51. doi:10.1093/molbev/msab152.
56. Jiang H, Qian Y, Zhang Z, Meng M, Deng Y, Wang G, et al. Chromosome-level genome assembly and whole-genome resequencing of topmouth culter (Culter alburnus) provide insights into the intraspecific variation of its semi-buoyant and adhesive eggs. Molecular ecology resources. 2023;23 8:1841-52. doi:10.1111/1755-0998.13845.
57. Ye M. DESCRIPTION OF A NEW GENUS AND SPECIES OF DANIONINAE FROM CHINA

(CYPRINIFORMES: CYPRINIDAE). 1983.
58. Chen F, Xue G, Wang YK, Zhang HC, Clift PD, Xing YW, et al. Evolution of the Yangtze River and its biodiversity. Innovation (Cambridge (Mass)). 2023;4 3:100417. doi:10.1016/j.xinn.2023.100417.
59. Feng CG, Wang K, Xu WJ, Yang LD, Wanghe KY, Sun N, et al. Monsoon boosted radiation of the endemic East Asian carps. Sci China Life Sci. 2023;66 3:563-78. doi:10.1007/s11427-022-2141-1.
60. Feng SH, Bai M, Rivas-Gonzalez I, Li C, Liu SP, Tong YJ, et al. Incomplete lineage sorting and phenotypic evolution in marsupials. Cell. 2022;185 10:1646-60.e18. doi:10.1016/j.cell.2022.03.034.
61. Martin SH, Davey JW and Jiggins CD. Evaluating the use of ABBA-BABA statistics to locate introgressed loci. Mol Biol Evol. 2015;32 1:244-57. doi:10.1093/molbev/msu269.
62. Ren L, Li WH, Qin QB, Dai H, Han FM, Xiao J, et al. The subgenomes show asymmetric expression of alleles in hybrid lineages of Megalobrama amblycephala x Culter alburnus. Genome Res. 2019;29 11:1805-15. doi:10.1101/gr.249805.119.
63. Chen J, Liu H, Gooneratne R, Wang Y and Wang WM. Population Genomics of Megalobrama Provides Insights into Evolutionary History and Dietary Adaptation. Biology (Basel). 2022;11 2:186. doi:10.3390/biology11020186.
64. Tautz D and Domazet-Lošo T. The evolutionary origin of orphan genes. Nat Rev Genet. 2011;12 10:692-702. doi:10.1038/nrg3053.
65. Hayden B, Palomares MLD, Smith BE and Poelen JH. Biological and environmental drivers of trophic ecology in marine fishes - a global perspective. Sci Rep. 2019;9 1:11415. doi:10.1038/s41598-019-47618-2.
66. Zhou X, Khan S, Huang DB and Li L. V-Set and immunoglobulin domain containing (VSIG) proteins as emerging immune checkpoint targets for cancer immunotherapy. Front Immunol. 2022;13:938470. doi:10.3389/fimmu.2022.938470.
67. Miao EA, Rajan JV and Aderem A. Caspase-1-induced pyroptotic cell death. Immunological reviews. 2011;243 1:206-14.
68. Felthaus O, Gosau M and Morsczeck C. ZBTB16 induces osteogenic differentiation marker genes in dental follicle cells independent from RUNX2. Journal of periodontology. 2014;85 5:e144-51. doi:10.1902/jop.2013.130445.
69. Sribudiani Y, Chauhan RK, Alves MM, Petrova L, Brosens E, Harrison C, et al. Identification of Variants in RET and IHH Pathway Members in a Large Family With History of Hirschsprung Disease. Gastroenterology. 2018;155 1:118-29.e6. doi:10.1053/j.gastro.2018.03.034.
70. Rai K, Sarkar S, Broadbent TJ, Voas M, Grossmann KF, Nadauld LD, et al. DNA demethylase activity maintains intestinal cells in an undifferentiated state following loss of APC. Cell. 2010;142 6:930-42. doi:10.1016/j.cell.2010.08.030.
71. Zoodsma JD, Keegan EJ, Moody GR and Bhandiwad AA. Disruption of grin2B, an ASD-associated gene, produces social deficits in zebrafish. 2022;13 1:38. doi:10.1186/s13229-022-00516-3.
72. Zoodsma JD, Chan K, Bhandiwad AA, Golann DR, Liu G, Syed SA, et al. A Model to Study NMDA Receptors in Early Nervous System Development. The Journal of neuroscience : the official journal of the Society for Neuroscience. 2020;40 18:3631-45. doi:10.1523/jneurosci.3025-19.2020.
73. Li DY, Jiang XD, Gong W and Li CY. Tectonic uplift along the northeastern margin of the Qinghai–Tibetan Plateau: Constraints from the lithofacies sequence and deposition rate of the Qaidam Basin. Tectonophysics. 2022;827:229279. doi:10.1016/j.tecto.2022.229279.
74. Hren MT, Sheldon ND, Grimes ST, Collinson ME, Hooker JJ, Bugler M, et al. Terrestrial cooling in Northern Europe during the eocene-oligocene transition. Proceedings of the National Academy of Sciences of the United States of America. 2013;110 19:7562-7. doi:10.1073/pnas.1210930110.

75. Xing YC, Zhang CG, Fan E and Zhao YH. Freshwater fishes of China: species richness, endemism, threatened species and conservation. Divers Distrib. 2016;22.
76. Murat F, Mbengue N, Winge SB, Trefzer T, Leushkin E, Sepp M, et al. The molecular evolution of spermatogenesis across mammals. Nature. 2023;613 7943:308-16. doi:10.1038/s41586-022-05547-7.
77. Brawand D, Soumillon M, Necsulea A, Julien P, Csardi G, Harrigan P, et al. The evolution of gene expression levels in mammalian organs. Nature. 2011;478 7369:343-8. doi:10.1038/nature10532.
78. Carruthers M, Edgley DE, Saxon AD, Gabagambi NP, Shechonge A, Miska EA, et al. Ecological Speciation Promoted by Divergent Regulation of Functional Genes Within African Cichlid Fishes. Mol Biol Evol. 2022;39 11:msac251. doi:10.1093/molbev/msac251.
79. Olave M, Nater A, Kautt AF and Meyer A. Early stages of sympatric homoploid hybrid speciation in crater lake cichlid fishes. Nature communications. 2022;13 1:5893. doi:10.1038/s41467-022-33319-4.
80. He S, Li L, Lv LY, Cai WJ, Dou YQ, Li J, et al. Mandarin fish (Sinipercidae) genomes provide insights into innate predatory feeding. Commun Biol. 2020;3 1:361. doi:10.1038/s42003-020-1094-y.
81. Jawad LA, Agha GF, Abdullah SMA, Aguilar G and Qasim AM. Morphology and morphometry of pharyngeal bone and teeth in cyprinid species from the Kurdistan Region, Iraq. Anatomical record. 2022;305 11:3356-66. doi:10.1002/ar.24906.
82. Gu QH, Yuan H, Zhong H, Wei ZH, Shu YQ, Wang J, et al. Spatiotemporal characteristics of the pharyngeal teeth in interspecific distant hybrids of cyprinid fish: Phylogeny and expression of the initiation marker genes. Front Genet. 2022;13:983444. doi:10.3389/fgene.2022.983444.
83. Hartenstein V and Martinez P. Structure, development and evolution of the digestive system. Cell Tissue Res. 2019;377 3:289-92. doi:10.1007/s00441-019-03102-x.
84. Xiao J, Kang XW, Xie LH, Qin QB, He ZL, Hu FZ, et al. The fertility of the hybrid lineage derived from female Megalobrama amblycephala x male Culter alburnus. Anim Reprod Sci. 2014;151 1-2:61-70. doi:10.1016/j.anireprosci.2014.09.012.
85. Owens GL and Samuk K. Adaptive introgression during environmental change can weaken reproductive isolation. Nat Clim Change. 2019;10 1:58-62. doi:10.1038/s41558-019-0628-0.

Figure 1

Click here to access/download;Figure;Figure 1.tif

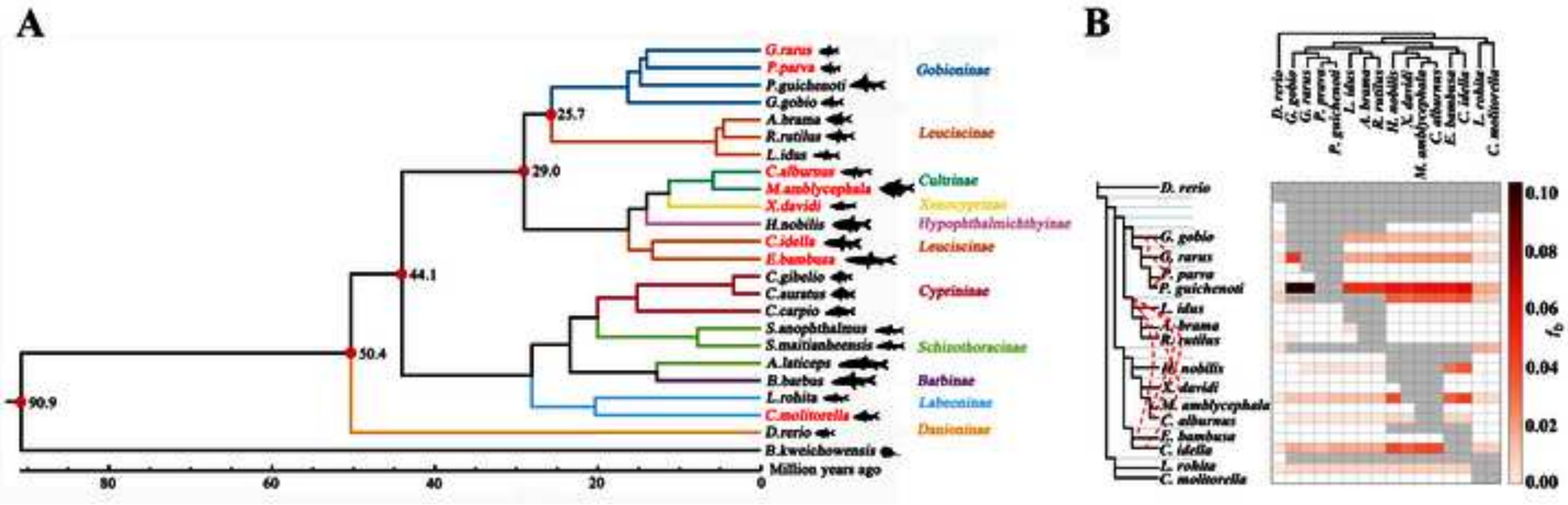

Figure 2

Click here to access/download;Figure;Figure 2.tif

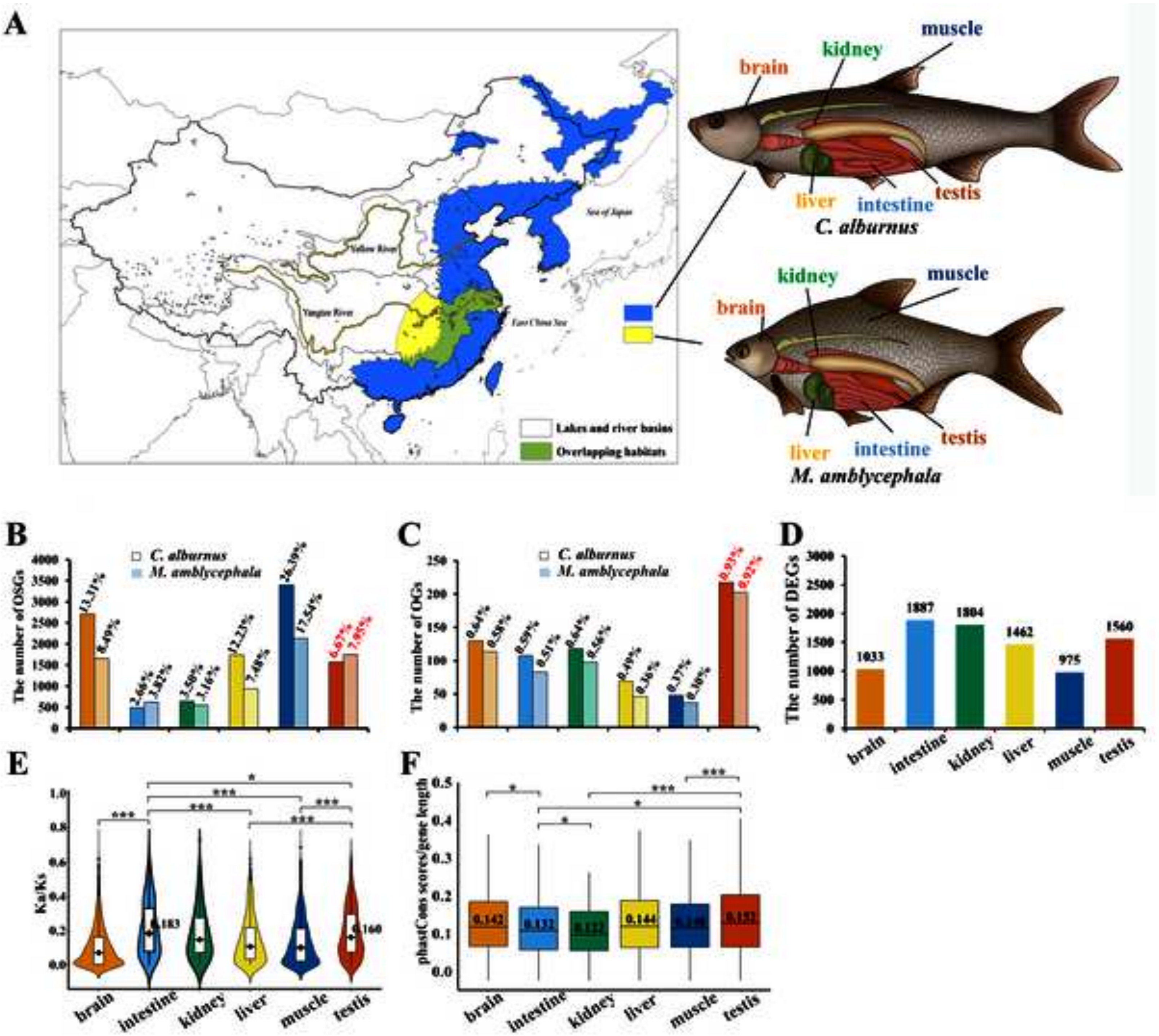

Figure 3

Click here to access/download;Figure;Figure 3.tif

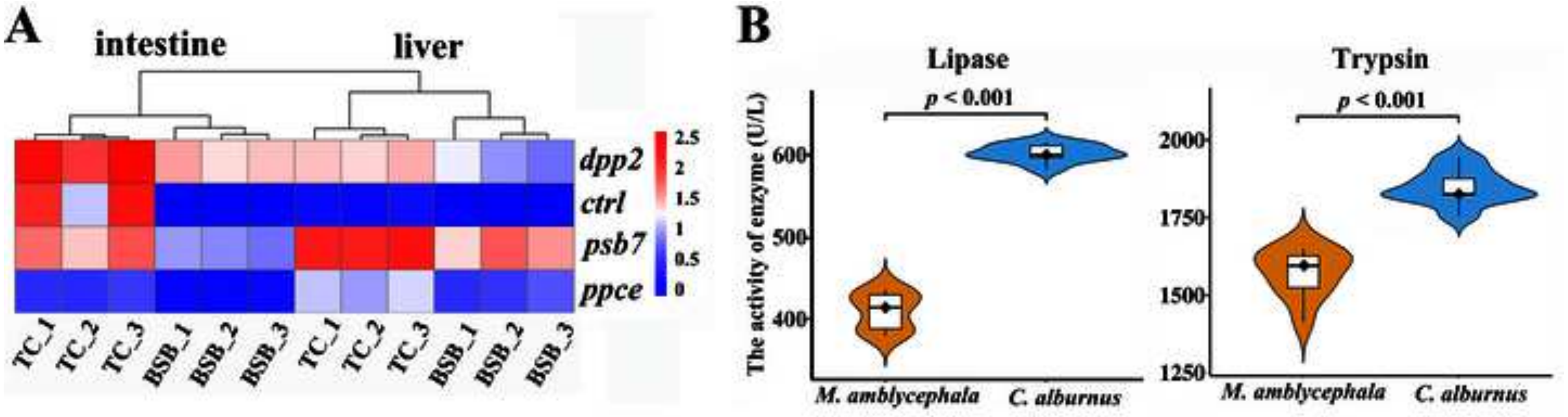

Figure 4

Click here to access/download;Figure;Figure 4.tif

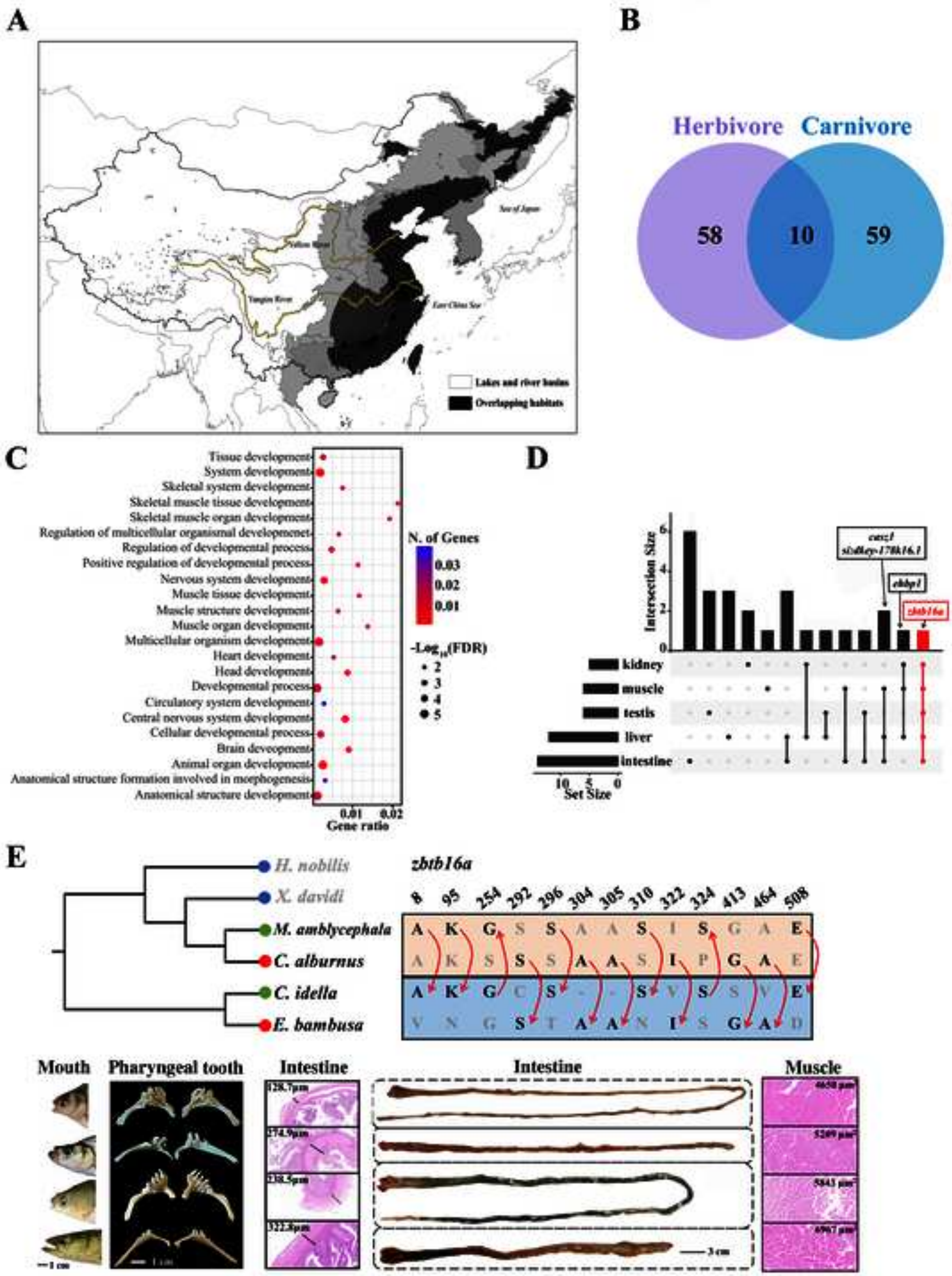

Click here to access/download
Supplementary Material
Supplementary Figures.docx

Click here to access/download
Supplementary Material
Supplementary Tables.xlsx
