# Supplementary material for: Genomes reveal pervasive distant hybridization in nature among cyprinid fishes
Source: Gigascience. 2025 Jan 30;14:giae117. doi: 10.1093/gigascience/giae117 (PMC11779505; doi:10.1093/gigascience/giae117)
Supplement: giae117_GIGA-D-24-00199_Revision_1 [file giae117_giga-d-24-00199_revision_1.pdf]

GigaScience

Genomes reveal pervasive distant hybridization in nature among cyprinid fishes

--Manuscript Draft--

**Manuscript Number:**	GIGA-D-24-00199R1
**Full Title:**	Genomes reveal pervasive distant hybridization in nature among cyprinid fishes
**Article Type:**	Data Note
**Funding Information:**	National Natural Science Foundation of China (32293252) — Not applicable<br>National Natural Science Foundation of China (32341057) — Not applicable<br>National Natural Science Foundation of China (U19A2040) — Not applicable<br>Hunan Provincial Natural Science Foundation (2022JJ10035) — Not applicable<br>Huxiang Young Talent Project of China (2021RC3093) — Not applicable<br>National Key Research and Development Plan Program (2023YFD2401602) — Not applicable<br>Laboratory of Lingnan Modern Agriculture Project (NT2021008) — Not applicable<br>Special Funds for Construction of Innovative Provinces in Hunan Province (2021NK1010) — Not applicable<br>Earmarked Fund for China Agriculture Research System (CARS-45) — Not applicable<br>111 Project (D20007) — Not applicable

Abstract:

Abstract

Background: Genomic data has unveiled a fascinating aspect of the evolutionary past, showing that the mingling of different species through hybridization has left its mark on the histories of numerous life forms. However, the relationship between hybridization events and the origins of cyprinid fishes remains unclear.

Results: In this study, we generated de novo assembled genomes of eight cyprinid fishes and conducted phylogenetic analyses on 24 species. Widespread allele sharing across species boundaries was observed within seven subfamilies of cyprinid fishes. Based on a systematic analysis of multiple tissues, we found that the testis exhibited a conserved pattern of divergence between the herbivorous Megalobrama amblycephala and the carnivorous Culter alburnus, suggesting a potential link to incomplete reproductive isolation. Significant differences in the expression of four genes (dpp2, ctrl, psb7, and ppce) in the liver and intestine, accompanied by variations in enzyme activities, indicated swift divergence in digestive enzyme secretion. Moreover, we identified introgressed genes linked to organ development in sympatric fishes with analogous feeding habits within the Cultrinae and Leuciscinae subfamilies.

Conclusions: Our findings highlight the significant role played by incomplete reproductive isolation and frequent gene flow events, particularly those associated with the development of digestive organs, in driving speciation among cyprinid fishes in diverse freshwater ecosystems.

**Corresponding Author:**	Li Ren<br>Hunan Normal University<br>Changsha, Hunan CHINA
**Corresponding Author Secondary Information:**	

Powered by Editorial Manager® and ProduXion Manager® from Aries Systems Corporation

**Corresponding Author's Institution:**	Hunan Normal University
**Corresponding Author's Secondary Institution:**	
**First Author:**	Li Ren
**First Author Secondary Information:**	
**Order of Authors:**	Li Ren
	Xiaolong Tu
	Mengxue Luo
	Qinzhi Liu
	Jialin Cui
	Xin Gao
	Hong Zhang
	Yakui Tai
	Yiyan Zeng
	Mengdan Li
	Chang Wu
	Wuhui Li
	Jing Wang
	Dongdong Wu
	Shaojun Liu
**Order of Authors Secondary Information:**	
**Response to Reviewers:**	Dear Dr. Hongfang Zhang,<br>Please find the revised manuscript entitled “Genomes reveal pervasive distant hybridization in nature among cyprinid fishes” (GIGA-D-24-00199R1), which we would like to submit for publication as original research in GigaScience.<br>The comments provided by the editors and reviewers have enabled us to further improve the quality of our manuscript. In the revised version of the manuscript, we have made comprehensive revisions to enhance the conciseness and biological perspective of the entire text. We have highlighted the revised sections in yellow. Detailed explanations and our point-by-point responses to each of the comments are provided in the following pages.<br><br>We are looking forward to hearing from you at your earliest convenience.<br><br>Sincerely!<br><br>Corresponding author: Shaojun Liu<br>State Key Laboratory of Developmental Biology of Freshwater Fish, Hunan Normal University, Changsha, 410081, P.R. of China<br><br>Corresponding author: Dongdong Wu<br>State Key Laboratory of Genetic Resources and Evolution, Kunming Institute of Zoology, Chinese Academy of Sciences, Kunming, 650201, China<br><br><br>Responses to the comments of the editors:<br>Please include a point-by-point within the 'Response to Reviewers' box in the submission system. Please ensure you describe additional experiments that were carried out and include a detailed rebuttal of any criticisms or requested revisions that you disagreed with. Please also ensure that your revised manuscript conforms to the journal style, which can be found in the Instructions for Authors on the journal homepage. If the data and code has been modified in the revision process please be

sure to update the public versions of this too.
Answer
We appreciate your positive feedback. We have addressed all of the reviewers' comments and made the necessary revisions to the manuscript. Furthermore, we have ensured that the manuscript adheres to the journal's formatting requirements. Please note that we have not updated the code.

Responses to the comments of the reviewer 1:
This paper reported their work of genome sequencing and assembly of eight cyprinid fishes and phylogenetic analyses using genomic sequences from a couple of dozens of species. They found widespread allele sharing across species boundaries within seven subfamilies of cyprinid fishes. The major findings are incomplete reproductive isolation and frequent gene flow among some of these species. In spite of being expected, this research provided empirical evidence, which is significant. As such, it is suitable for publication in GigaScience. A number of minor concerns, however, need to be addressed before publication:
1) The authors need to tone down in drawing conclusions related to genes, gene expression and behavior (e.g., food selection) and organ development. Although the differences in genes and gene expression may eventually be responsible for the observed biological differences in traits, a direct link of gene expression, as presented with differentially expressed genes, to traits is still far-fetched.
Answer 1
Thank you for your insightful comments. We acknowledge that our study has demonstrated correlations between gene expression and phenotypic traits, but these correlations do not necessarily establish a causal relationship. We have revised the manuscript to more accurately reflect the limitations of our study and have tempered the claims regarding the functional significance of the identified genes.
The details are as follows:
"Our findings provide preliminary evidence suggesting a potential rapid divergence in genetic diversity within the digestive organs of M. amblycephala and C. alburnus." (page 17, lines 5-7)
"Among these genes, dpp2, ctrl, psb7, and ppce were identified as potential genes involved in peptidase activity, exhibiting higher expression in the digestive organs (liver and intestine) of C. alburnus compared to M. amblycephala (Fig. 3a)." (page 17, lines 13-16)
"These findings suggest that the observed genetic diversity may be related to adaptations in digestive enzyme secretion, reflecting potential dietary adjustments." (page 17, lines 23-24)
"Gene flow and its potential impact on feeding habits" (page 18, line 2)
"Zbtb16a (linked to osteogenic differentiation (Felthaus et al. 2014)) exhibited the broadest expression pattern among all introgressed genes, being detected in five different tissues and organs (Fig. 4d). This suggests that zbtb16a may be a hotspot for introgression events between Cultrinae and Leuciscinae." (page 18, lines 24-25, page 19, lines 1-2)

2) The readability of the paper needs to be improved. The paper, as it is written the way it stands now, is fairly technical. The authors are suggested to present the major findings in biologist's language such that its readability is enhanced.
Answer 2
We appreciate your kind comments. The Discussion section has been revised to incorporate a more biological viewpoint.
The major modifications are as follows:
"Among these, significant gene flow was detected between carnivorous C. alburnus (subfamily Cultrinae) and E. bambusa (subfamily Leuciscinae) (Z-score > 45.8, f4–ratio = 0.039, and p-value < 0.001)." (page 18, lines 4-6)
"Moreover, 84 categories (38.36%) for biological processes and 16 categories (66.67%) for molecular functions were shared between carnivores and herbivores (FDR < 0.05, Fig. 4c and Supplemental File 2: Figs. S9-S10)." (page 18, lines 19-22)
"Zbtb16a (linked to osteogenic differentiation (Felthaus et al. 2014)) exhibited the broadest expression pattern among all introgressed genes, being detected in five different tissues and organs (Fig. 4d). This suggests that zbtb16a may be a hotspot for introgression events between Cultrinae and Leuciscinae. " (page 18, lines 24-25, page 19, lines 1-2)

Powered by Editorial Manager® and ProduXion Manager® from Aries Systems Corporation

"Chromosome-scale genomes were assembled for blunt snout bream (Megalobrama amblycephala, BSB) and topmouth culter (Culter alburnus, TC) using 204.8 Gb Hi-C data. The resulting assemblies exhibited scaffold N50 values of 42.91 Mb and 39.60 Mb, respectively (Table 1 and Supplemental File 1: Tables S1-S2)." (page 13, lines 1-4)

Responses to the comments of the reviewer 2:
Reviewer #2: This study aims to unravel the intricate relationship between hybridization events and the evolutionary origins of cyprinid fish. By assembling the high-quality genomes of eight cyprinid fish species and integrating publicly available genomic data, this study sheds light on the pivotal role of gene flow events in the evolution of cyprinid fish. Through comprehensive multi-tissue transcriptomic analyses, the study delves into the intricate interplay among reproductive isolation, gene flow dynamics, and the development of digestive organs. This is an intriguing study exploring the mutual influence between the origin of hybridization and dietary adaptation in cyprinid fishes.
Answer
We are grateful for the insightful comments. These suggestions have greatly enhanced the quality of our manuscript.

To enhance the research's value and readability, it is advisable to address some minor issues before publication:
1. The figure legends of both main and supplemental figures are generally uninformative and lack adequate detail. A few examples:
Scale of the heatmaps. Are these z-scores?
Several figures feature TC and BSB acronyms. While I understand each of these refers to either M. amblycephala or C. alburnus, I cannot find anywhere in the manuscript or supplemental material any information on which is which.
Answer 1
For Figure 1B, Scale of the heatmap is f-branch value. We have revised it in the Figure 1B.

We have added a description of the full Latin names corresponding to the acronyms used in the legends of Figures 3, S2, S7, S10, and S11.
"Figure 3: Diet divergences between M. amblycephala and C. alburnus. (a) The four genes relating to differential expression between M. amblycephala (BSB) and C. alburnus (TC) in both the intestine and liver (three biological replicates showed "_1", "_2", and "_3")." (page 31, lines 20-23 in main text)
"Figure S2. The Hi-C interaction heatmap of 24 linkage groups in the genomes of Megalobrama amblycephala and Culter alburnus." (page 2, lines 16-17 in Supplementary Figures)
"Figure S7. The DEGs (M. amblycephala vs. C. alburnus) associated with diet habit. a. The heatmap of the DEGs in the intestine. The hydrolyzing O-glycosyl compounds and peptidase activity in Molecular Function, as well as carbohydrate metabolic process and lipid catabolic process in Biological Process. TC: C. alburnus, BSB: M. amblycephala." (page 4, lines 40-41 in Supplementary Figures)
"Figure S10. The distribution of enriched functional categories (FDR < 0.05) in Biological Process and Molecular Function for the introgressed genes. Carnivore: C. alburnus and Elopichthys bambusa, herbivore: M. amblycephala and Ctenopharyngodon idella." (page 6, lines 53-55 in Supplementary Figures)
"Figure S11. Heatmap exhibiting the expression of introgressed genes in the carnivorous (C. alburnus and E. bambusa) and herbivorous (M. amblycephala and C. idella) fishes. TC: C. alburnus, BSB: M. amblycephala." (page 6, lines 58 in Supplementary Figures)

2. Add a sentence in the legend describing the color scheme used in Figure 3.
Answer 2
In Figure 3A, we have implemented a new color scheme to differentiate between liver and intestine samples. Furthermore, we have added a scale bar with appropriate units to the heatmap to provide a quantitative measure of gene expression levels.

Figure 3: Diet divergences between M. amblycephala and C. alburnus
(A) The four genes relating to differential expression between M. amblycephala (BSB) and C. alburnus (TC) in both the intestine and liver (three biological replicates showed

Powered by Editorial Manager® and ProduXion Manager® from Aries Systems Corporation

“_1”, “_2”, and “_3”). (B) Significant differences in the enzyme activity of lipase and trypsin for the comparison between M. amblycephala and C. alburnus.

3. Please unify Mya in the introduction and results sections (MYA). Pay attention to the writing as sometimes it is a full name and sometimes it is an abbreviation.
Answer 3
We appreciate your feedback. To ensure consistency, we have standardized the age terminology throughout the manuscript, using "million years ago" (MYA) in all relevant instances.

You can consider including a "List of Abbreviations" section at the beginning of the manuscript to list all abbreviations used and their full forms. This would improve readability for future readers.
Go through the entire manuscript one more time to ensure consistency in terminology, punctuation, and formatting.
Answer 4
To enhance clarity and readability, we have added a section of abbreviations at the end of the manuscript, providing the full terms for all abbreviations used. (page 23, lines 24-25, page 24, lines 1-3)

Responses to the comments of the reviewer 3:
Reviewer #3: Ren et al. present new cyprinid genome assemblies and RNA-seq expression data to evaluate hybridization occurrence across lineages in this ecologically diverse and species family.
Assembly Quality: The genome assemblies appear to be high quality. I have noted some confusion about methods and results which could be clarified.
Answer 1
Thanks for your comments on the genome assembly. In the revised manuscript, we have highlighted the revised methods and results in yellow.

The data was analyzed extensively, which I appreciate, but the manuscript is very long and the extensive analyses make the paper difficult to read and follow in detail. Honing down a tighter story in favor of fewer, but more relevant analyses would be helpful.
Answer 2
Your comments have significantly improved the clarity of our manuscript. We have made substantial revisions to the presentation of our analyses, particularly in the results section, which are now highlighted in yellow. These changes should enhance the reader's understanding of our findings.

Hybridization tests: forgive my ignorance on how these tests work, but I wonder if there is a possibility that significant departures from null for ABBA-BABA or f4 tests could instead be the result of which sex was sequenced for each species, e.g., if there is a bias introduced by some genome assemblies coming from male vs. female parents? I might have missed the SNP calling methods but this could affect the results - did these come from phased assemblies? Or mapping reads to collapsed haploid assemblies? For the polyploid species, this could also impact hybridization tests. How much of this signal of introgressive hybridization is due to long (or short) histories of artificial breeding (sometimes intentional) in these species?
Answer 3
As described in the hybridization tests on page 15, lines 13-17, while no natural hybrid populations of M. amblycephala and C. alburnus were found in overlapping habitats, we successfully produced fertile F1 hybrids through artificial breeding. Subsequent self-crossing of these F1 hybrids generated F2 generations, establishing multi-generational hybrid lines between the two species. These results indicate the absence of reproductive isolation between M. amblycephala and C. alburnus.
Genome assemblies were constructed using DNA from non-inbred individuals. The absence of sex chromosomes in these cyprinid fish reduced potential biases associated with sex-specific genomic regions. SNPs were identified using a modified version of Chen's method (Chen and Qiu 2019), aligning whole genomes to the zebrafish reference (page 9, lines 16-20). Our analysis focused solely on diploid genomes from non-inbred populations to avoid complications introduced by phased assemblies or inbreeding. It has come to our attention that previous studies have employed ABBA-BABA or f4 ratio tests to investigate gene flow without accounting for

Powered by Editorial Manager® and ProduXion Manager® from Aries Systems Corporation

sex-specific effects (Flury et al. 2023).
While phylogenetic analyses were conducted for the all 24 species, ABBA-BABA tests were restricted to 17 non-polyploid cyprinid fish. The detail decryption is as follow:
“The conserved scores of the 17 non-polyploid species were estimated using the phastCons tool from the phast packages (Cooper et al. 2005). VCF files for each species were generated by aligning whole genomes to the zebrafish genome using Chen's methods (Chen and Qiu 2019). To investigate gene flow, we exclusively analyzed the 17 non-polyploid genomes from non-inbred populations using the ABBA-BABA test implemented in the Dsuite (0.4 r38) software (Malinsky et al. 2021) with the D-statistic method” (page 9, lines 15-20)
To further clarify our findings, we rephrased the results section as follows:
“To investigate this, we conducted gene flow analysis and observed pervasive introgression among the 17 non-polyploid cyprinid fishes (f4-ratio > 0.0006, Z-score > 3, and p-value < 0.05) (Fig. 1b).” (page 14, lines 19-21)
You raised an interesting question about the potential impact of artificial breeding on our observed introgression signals. While our samples were collected from wild populations, as detailed in the 'sample collection' section of the Methods, we cannot entirely rule out the possibility of escaped farmed individuals. Addressing this question definitively would require additional studies and a more complex experimental design. However, this is an intriguing area for future research.

Conclusion (pg 2, line 15-17): Why does gene flow drive speciation? The mechanism here is unclear, as pure introgression simply homogenizes existing variation (although more generally I recognize the *potential* creative role of hybridization). Diet can play a role in habitat preferences, but this only matters if those differences prevent reproduction (and persist at that time), but I do not see evidence of actual mechanistic drivers of reproductive isolation (the authors correctly acknowledge this), but the divergence of dietary enzymes is likely even in the absence of hybridization, particularly as copy numbers of these expand and contract (see amylase gene copy number variation in domestic mammals, for example). That variation is not related to hybridization, so the link is tenuous.
Answer 4
We appreciate your insightful comments on the role of gene flow in driving speciation. We agree that gene flow is a complex phenomenon and its impact on speciation can vary among species. Previous studies have shown that gene flow can promote speciation in some cases, such as through ecological niche divergence or genomic conflict. However, in other cases, gene flow can hinder speciation, for example, by homogenizing existing variation.
In our study, we found frequent introgression among East Asian Cyprinidae, suggesting that gene flow may have a significant impact on the speciation of these species. However, we were unable to fully determine the specific mechanisms by which gene flow drives speciation in these species. Future studies may need to further explore potential mechanisms, such as ecological niche divergence, genomic conflict, or hybrid vigor.
We thank you for your comments and agree that differences in dietary enzymes may be an important factor in the speciation of East Asian Cyprinidae. However, our study did not directly demonstrate a causal relationship between dietary enzyme differences and speciation. Future research may need to further explore the link between dietary enzyme differences and speciation.

Minor comments:
1. Pg2, line 9: "conservative divergence in the testis" is unclear. I assume this means minimal expression differences?
Answer 1
Our comparative analysis of six tissues in M. amblycephala and C. alburnus indicated that, contrary to the reviewer's suggestion, the testis showed a relatively conserved pattern of evolutionary change. This conclusion was drawn from an assessment of multiple metrics, including gene expression divergence, rates of nonsynonymous to synonymous substitutions (Ka/Ks), and evolutionary conservation scores.
The detailed revisions are as follows:
“Based on a systematic analysis of multiple tissues, we found that the testis exhibited a conserved pattern of divergence between the herbivorous Megalobrama amblycephala and the carnivorous Culter alburnus, suggesting a potential link to incomplete reproductive isolation.” (page 2, lines 8-11)

2. Pg3, line 13 - the terms 'hybridization' and 'introgression' are conflated here - they are not synonymous.
Answer 2
Thank you for pointing out the error in our previous statement. We have revised the sentence to:
“Introgression plays a significant and frequent role in adaptive evolution” (page 3, line 13)

3. Pg3, line 14 - the Mallet citation refers to 10% of animal *species* being involved in hybridization, rather than 10% of wild animals (individuals).
Answer 3
We have revised this sentence according to the kind comment as follow:
"At least 10% of animal species are involved in hybridization, although most hybrid individuals have low viability or are sterile (Mallet 2005)" (page 3, lines 13-15)

4. Pg3, line 15 - The sentence is unclear. I think the authors are trying to say that intergeneric hybrids are rare in birds and mammals because of reproductive isolation, but this is tautological, unless the authors have a different definition of reproductive isolation.
Answer 4
We have revised this sentence according to the kind comment as follow:
“While reproductive isolation (RI) is frequently observed in intergeneric hybridization among birds and mammals, it is less common in cyprinid fish species, as evidenced by the documented instances of disrupted isolation” (page 3, lines 15-17)

5. Pg5, line 7 - I don't quite understand. The three males are hybrid offspring of the two parental species? Which was the maternal vs paternal species?
Answer 5
To clarify, we have rephrased the sentence as follows:
“Three sexually mature male Culter alburnus and Megalobrama amblycephala, raised in identical controlled conditions for 24 months post-hatching, were bred at the Engineering Center of Polyploid Fish Breeding, National Education Ministry, Changsha, Hunan, China. The broodstock were sourced from the Yangtze River (30°25'56" N, 114°50'32" E).” (page 5, lines 6-9)

Pg6, line 8 - this says nanopore sequencing, but later it says PacBio was conducted. Above (line 3) it says Ampure beads, manufacturer PacBio, but this also is incorrect (Beckman-Coulter, Inc.).
Answer 6
There was an error in the methodology regarding the sequencing platform used for C. alburnus and M. amblycephala. While we employed Nanopore sequencing for these two species, the other six fish species (C. idella, C. molitorella, P. parva, X. davidi, G. rarus, and E. bambusa) were sequenced using PacBio Sequel II. Specifically, the statement regarding the use of AMPure PB beads for purifying the genomic DNA of C. alburnus and M. amblycephala was incorrect. We have corrected this error in the methods section as follow:
“After quality checking, the genomic DNA of C. alburnus and M. amblycephala was randomly sheared using Megaruptor (Diagenode, Denville, NJ, USA). DNA was size-selected using a SPRI bead protocol. The purity of the extracted DNA was determined using a Nanodrop spectrophotometer (Thermo Fisher Scientific). All procedures were carried out at room temperature. Large DNA fragments were separated using BluePippin DNA Size Selection System. DNA damage repair and end-repair were performed. Barcoded overhang hairpin adapters were ligated to the fragment ends. The connection reaction was performed using Ligation Sequencing Kit (Oxford Nanopore, SQK-LSK108). A constructed DNA library was quantified using Qubit. Lastly, sequencing was performed using Nanopore Sequencing.” (page 5, line 25, page 6, lines 1-8)

7. Was 'ligation sequencing kit' (line 7) Oxford Nanopore Technologies, Inc? Or another kit? Pg 12, lists Oxford Nanopore, but no mention of this is in the methods, including which platform.
Answer 7
Thank you for your kind comments! We have incorporated the information about the

Powered by Editorial Manager® and ProduXion Manager® from Aries Systems Corporation

	'ligation sequencing kit' and revised the sentence as follows:<br>“The connection reaction was performed using Ligation Sequencing Kit (Oxford Nanopore, SQK-LSK108).” (page 6, lines 6-7)<br><br>8. Pg6, line 25 - No citation given for the 'standard protocol' for DNBSEQ-T7 - could the authors provide one?<br>Answer 8<br>Thank you for your valuable comment! We have added a reference to the literature: “Comparison between MGI and Illumina sequencing platforms for whole genome sequencing.” (page 27, lines 20-21)<br><br>9. Pg7, line9 - some of these species already have high quality reference genomes - what is the rationale for additional sequencing? What sex were the individuals that were sequenced for genome assembly? Were haplotigs purged from draft assemblies or evaluated? Contamination from microbiome?<br>Answer 9<br>Prior to our study, high-quality genome assemblies generated using PacBio HiFi reads were absent for these six species. These high-quality assemblies are essential for conducting comprehensive comparative genomic analyses. Currently, to our knowledge, only C. idella and G. rarus among these six species have publicly high-quality genome assemblies produced using PacBio HiFi reads.<br>Due to the immature stage of some collected fish, we were unable to collect sex information for these species.<br>The assembled genome is not a haplotype genome. Comparisons of our assembly with published genomes of the same or closely related species indicate that its size is consistent with the expected genome size for this species, rather than representing a haploid genome.<br>We have rigorously assessed our assembly for microbial contamination through both annotation analyses and National Genomics Data Center (NGDC) submission checks. These analyses have confirmed the absence of significant microbial contamination in our data.<br><br>10. Pg8, line 4 - its unclear why the reference genomes of e.g., Ctenopharyngodon idella, were not used for genome annotation, given these are the species being assembled.<br>Answer 10<br>Yes, there is a PacBio Sequel-based genome assembly of Ctenopharyngodon idella available online, released in February 2023 (https://ftp.ncbi.nlm.nih.gov/genomes/all/GCF/019/924/925/GCF_019924925.1_HZGC01/). However, we had already completed our analysis by that time. Therefore, when we conducted genome annotation, we did not use the annotation file from this specific genome version.<br><br>11. Pg 12, line 20 - how were these data split across the various species and sequencing technologies?<br>Answer 11<br>We have presented a detailed illustration of this in Supplementary Table 1.<br>Moreover, we have elaborated on this point in the revised manuscript as follows:<br>“A total of eight species of cyprinid fishes from East Asia were sequenced using PacBio HiFi or Oxford Nanopore technology, resulting in over 602.21 Gb of raw data (Supplemental File 1: Tables S1).” (page 12, lines 23-25)<br><br>12. Pg 14, line 19-20 - what about the various genome papers that have been published and included genome scale phylogenomics? Are the results concordant with previous findings? (Molecular vs morphology discordance is expected).<br>Answer 12<br>By integrating our phylogenomic analysis with previous genomic studies, we have estimated divergence times for major lineages within the Cyprinidae family.<br>Our analysis suggested that the common ancestor of extant cyprinids diverged between 81.9 and 100.0 MYA (Fig. 1a, page 14, lines 9-11), which is consistent with the estimated divergence time of 81.9-100.1 MYA for the most recent common ancestor of Nemacheilidae and Cyprinidae in a previous study (Xu et al. 2023).<br>Furthermore, we calculated that the ancestor of Danio rerio (subfamily: Danioninae) diverged early in the evolution of extant cyprinids, while the ancestor of Cirrhinus

	molitorella and Labeo rohita (subfamily: Labeoninae) diverged between 36.5 and 53.6 MYA (Fig. 1a and Supplemental File 1: Table S9, page 14 lines 11-14). These divergence times are comparable to the estimates of 40.4–48.6 MYA (Xu et al. 2023), ~37 MYa (Feng et al. 2023), and ~60 Mya (Xu et al. 2019) reported in previous studies. Additionally, we calculated that the divergence time of Leuciscinae (C. idella and E. bambusa), Hypophthalmichthyinae, Xenocypridinae, and Cultrinae is about 17 MYA. (Fig. 1a, page 14, lines 7-8). These results are generally consistent with previous estimates of 11.6–12.7 MYA (Xu et al. 2023), 15.8–14.5 Ma (Feng et al. 2023), and 12.69 Ma (Wang et al. 2024).<br>Finally, our analysis indicates that Cyprininae diverged from other subfamilies approximately 20 MYA (Fig. 1a), which is comparable to the estimate of 25.18 MYA (95% CI: 20.75–28.03 Ma) reported in a previous study (Li and Guo 2020).<br><br>13. Table 1 - it would be helpful to have duplicate and single copy (and missing and fragmented) busco information here to evaluate possible ploidy issues.<br>Answer 13<br>The BUSCO analysis results for the eight assembled genomes are summarized in Supplementary Table 3.<br><br>14. FigS1 - Branch support is needed here to evaluate phylogenetic support.<br>Answer 14<br>As shown in Figure S1, the bootstrap support values for all branches in the phylogenetic trees were 100%. We have included the above information in the legend of Figure S1.<br><br>Reference:<br>Chen L, Qiu Q. 2019. Large-scale ruminant genome sequencing provides insights into their evolution and distinct traits. 364(6446).<br>Cooper GM, Stone EA, Asimenos G, Green ED, Batzoglou S, Sidow A. 2005. Distribution and intensity of constraint in mammalian genomic sequence. Genome research 15(7): 901-913.<br>Felthaus O, Gosau M, Morsczeck C. 2014. ZBTB16 induces osteogenic differentiation marker genes in dental follicle cells independent from RUNX2. Journal of periodontology 85(5): e144-151.<br>Feng C, Wang K, Xu W, Yang L, Wanghe K, Sun N, Wu B, Wu F, Yang L, Qiu Q et al. 2023. Monsoon boosted radiation of the endemic East Asian carps. Science China Life sciences 66(3): 563-578.<br>Flury JM, Meusemann K, Martin S, Hilgers L, Spanke T, Böhne A, Herder F, Mokodongan DF, Altmüller J, Wowor D et al. 2023. Potential Contribution of Ancient Introgression to the Evolution of a Derived Reproductive Strategy in Ricefishes. Genome biology and evolution 15(8).<br>Li X, Guo B. 2020. Substantially adaptive potential in polyploid cyprinid fishes: evidence from biogeographic, phylogenetic and genomic studies. Proceedings Biological sciences / The Royal Society 287(1920): 20193008.<br>Malinsky M, Matschiner M, Svardal H. 2021. Dsuite - Fast D-statistics and related admixture evidence from VCF files. Mol Ecol Resour 21(2): 584-595.<br>Mallet J. 2005. Hybridization as an invasion of the genome. Trends Ecol Evol 20(5): 229-237.<br>Wang C, Yang L, Lu Y, Fang C, Gan X, Chen Y, He S. 2024. Genomic features for adaptation and evolutionary dynamics of four major Asian domestic carps. Science China Life sciences 67(6): 1308-1310.<br>Xu M-R-X, Liao Z-Y, Brock JR, Du K, Li G-Y, Chen Z-Q, Wang Y-H, Gao Z-N, Agarwal G, Wei KHC et al. 2023. Maternal dominance contributes to subgenome differentiation in allopolyploid fishes. Nature communications 14(1): 8357.<br>Xu P, Xu J, Liu GJ, Chen L, Zhou ZX, Peng WZ, Jiang YL, Zhao ZX, Jia ZY, Sun YH et al. 2019. The allotetraploid origin and asymmetrical genome evolution of the common carp Cyprinus carpio. Nature communications 10.
**Additional Information:**	
**Question**	**Response**
Are you submitting this manuscript to a special series or article collection?	No

Powered by Editorial Manager® and ProduXion Manager® from Aries Systems Corporation

**Experimental design and statistics**<br><br>Full details of the experimental design and statistical methods used should be given in the Methods section, as detailed in our Minimum Standards Reporting Checklist. Information essential to interpreting the data presented should be made available in the figure legends.<br><br>Have you included all the information requested in your manuscript?	Yes
**Resources**<br><br>A description of all resources used, including antibodies, cell lines, animals and software tools, with enough information to allow them to be uniquely identified, should be included in the Methods section. Authors are strongly encouraged to cite Research Resource Identifiers (RRIDs) for antibodies, model organisms and tools, where possible.<br><br>Have you included the information requested as detailed in our Minimum Standards Reporting Checklist?	Yes
**Availability of data and materials**<br><br>All datasets and code on which the conclusions of the paper rely must be either included in your submission or deposited in publicly available repositories (where available and ethically appropriate), referencing such data using a unique identifier in the references and in the "Availability of Data and Materials" section of your manuscript.<br><br>Have you have met the above requirement as detailed in our Minimum Standards Reporting Checklist?	Yes

Powered by Editorial Manager® and ProduXion Manager® from Aries Systems Corporation

Manuscript
Click here to access/download;Manuscript;Manuscript-Nov 27.docx

Genomes reveal pervasive distant hybridization in nature among cyprinid fishes

Li Ren[1,*], Xiaolong Tu[2,3,4,*], Mengxue Luo[1,*], Qinzhi Liu[1,*], Jialin Cui[1], Xin Gao[1], Hong Zhang[1], Yakui Tai[1], Yiyan Zeng[1], Mengdan Li[1], Chang Wu[1], Wuhui Li[1], Jing Wang[1], Dongdong Wu[2,3,†] and Shaojun Liu[1,†]

[1]State Key Laboratory of Developmental Biology of Freshwater Fish, Engineering Research Center of Polyploid Fish Reproduction and Breeding of the State Education Ministry, College of Life Sciences, Hunan Normal University, Changsha, 410081, China.

[2]State Key Laboratory of Genetic Resources and Evolution, Kunming Institute of Zoology, Chinese Academy of Sciences, Kunming, 650201, China.

[3]Kunming Natural History Museum of Zoology, Kunming Institute of Zoology, Chinese Academy of Sciences, Kunming, 650223, China.

[4]Kunming College of Life Science, University of the Chinese Academy of Sciences, Kunming, 650204, China.

†Corresponding author, Email: lsj@hunnu.edu.cn; wudongdong@mail.kiz.ac.cn

*These authors contributed equally to this work.

Li Ren [0000-0003-4613-0380]; Xiaolong Tu [0000-0001-5312-7089]; Mengxue Luo [0009-0003-5224-1738]; Qinzhi Liu [0009-0004-5532-8457]; Jialin Cui [0000-0001-5901-8107]; Xin Gao [0000-0001-7162-4177]; Hong Zhang [0009-0001-7442-3079]; Yakui Tai [0009-0004-4525-5813]; Yiyan Zeng [0009-0007-7706-532X]; Mengdan Li [0009-0003-5896-7564]; Chang Wu [0000-0002-7315-767X]; Wuhui Li [0000-0002-1960-2533]; Jing Wang[0000-0003-4380-4052]; Dongdong Wu [0000-0001-7101-7297]; Shaojun Liu [0000-0001-5761-8571].

Abstract

Background: Genomic data has unveiled a fascinating aspect of the evolutionary past, showing that the mingling of different species through hybridization has left its mark on the histories of numerous life forms. However, the relationship between hybridization events and the origins of cyprinid fishes remains unclear.

Results: In this study, we generated *de novo* assembled genomes of eight cyprinid fishes and conducted phylogenetic analyses on 24 species. Widespread allele sharing across species boundaries was observed within seven subfamilies of cyprinid fishes. Based on a systematic analysis of multiple tissues, we found that the testis exhibited a conserved pattern of divergence between the herbivorous *Megalobrama amblycephala* and the carnivorous *Culter alburnus*, suggesting a potential link to incomplete reproductive isolation. Significant differences in the expression of four genes (*dpp2*, *ctrl*, *psb7*, and *ppce*) in the liver and intestine, accompanied by variations in enzyme activities, indicated swift divergence in digestive enzyme secretion. Moreover, we identified introgressed genes linked to organ development in sympatric fishes with analogous feeding habits within the *Cultrinae* and *Leuciscinae* subfamilies.

Conclusions: Our findings highlight the significant role played by incomplete reproductive isolation and frequent gene flow events, particularly those associated with the development of digestive organs, in driving speciation among cyprinid fishes in diverse freshwater ecosystems.

Keywords: diet divergence, genetic introgression, phylogenomics, incomplete reproductive isolation

Introduction

Cyprinidae (Order Cypriniformes) is the largest and most diverse family of ray-finned fish, comprising about 13 subfamilies and 370 genera [1, 2]. This family includes popular aquarium fishes like goldfish and koi, as well as the valuable vertebrate model organism, the zebrafish [2]. As a family of freshwater fish, the origin time of Cyprinidae was estimated at 154 million years ago (MYA) [3] and they are now widely distributed in almost all types of water around the world [2]. Their great diversities in feeding and reproductive behaviors, as well as morphology, including body length (ranging from about 8 mm for *Paedocypris progenetica* to approximately 3 m for *Catlocarpio siamensis*) [4, 5] and digestive organs [6, 7] are intriguing to evolutionary biologists due to their phylogenetic relationships and adaptive radiation evolution. However, the narrow distribution ranges or small population sizes of Cyprinidae fish now face threats from human activity, such as overfishing, damming of upland rivers, pollution, habitat destruction, and novel viral infections [8].

Introgression plays a significant and frequent role in adaptive evolution [9]. At least 10% of animal species are involved in hybridization, although most hybrid individuals have low viability or are sterile [10]. While reproductive isolation (RI) is frequently observed in intergeneric hybridization among birds and mammals, it is less common in cyprinid fish species, as evidenced by the documented instances of disrupted isolation [11-13]. Natural selection, including variable water environments and deleterious homozygosity for small population sizes, brings great pressures to the survival of freshwater fish [14, 15]. Hybridization has the potential to generate genetic diversity and create opportunities for novel adaptive radiations, although it has been considered a breakdown of isolating mechanisms [16]. The low viability or sterility of hybrids could reinforce RI through selection for assortative mating and result in adaptive introgression [10]. The rate of introgression depends on the pressures of the freshwater environment and affects fish biodiversity [17]. Natural hybridization involving intergeneric hybridization was always observed among cyprinid fishes [18-20], while bisexual fertile progenies were detected in the laboratory experiments of various hybrid groups [13].

This evidence suggests that prezygotic isolation evolves more rapidly than postzygotic isolation in cyprinid fishes, challenging the assumption of the criticality of variation in dietary niche breadth for speciation. Now, the relationship between biodiversity and introgressive hybridization in cyprinid fishes is still obscure. Phylogenomics from whole genome sequences could provide us with more detailed evidence of it than the fragmented detective technologies, including ribosomal DNA (rDNA) [20], mtDNA, and microsatellites [21].

Diet plays a crucial role in the biodiversity and habitat distribution of fishes, and is influenced by differences in foraging behavior and digestive organ morphology [22]. The selection of diet is particularly important for sympatric species [23]. In the case of cyprinid fishes, four main categories of diet have been identified. These include herbivorous fishes (e.g. *Megalobrama amblycephala* and *Ctenopharyngodon idella*), carnivorous fishes (e.g. *Culter alburnus* and *Elopichthys bambusa*), filter-feeding fishes (e.g. *Hypophthalmichthys nobilis*), and omnivorous fishes (e.g. *Cyprinus carpio* and *Carassius auratus*) [24]. These fish species are distributed across different water layers to acquire various types of food resources. Cyprinid fishes have evolved unique adaptations for food digestion, such as advanced protrusible pharyngeal teeth, despite the absence of jaw teeth and stomachs [25]. The number and shape of teeth in cyprinid fishes exhibit significant variation and are used as phenotypic features for species classification [2]. In East African cichlids, the number of tooth rows on both jaws has been associated with specific feeding ecologies [26]. However, it remains unclear whether variations in the width of the dietary niche are critical for the biodiversity of cyprinid fishes.

In this study, we obtained the *de novo* assembled genome sequences of eight cyprinid fish species and conducted comparative genome analyses using a set of 24 high-quality assembled genome sequences. Through gene flow analyses, we investigated hybridization events and their contributions to the speciation of cyprinid fishes. Furthermore, we conducted evolutionary constraints analyses on various tissues and organs and investigated their divergence between *M. amblycephala* and *C. alburnus*. Our findings emphasize the significance of gene flow events in the origin of cyprinid fishes and the

functional divergence that drives speciation.

Methods

Sample collection

Three sexually mature male *Culter alburnus* (NCBI:txid194366) and *Megalobrama amblycephala* (NCBI:txid75352), raised in identical controlled conditions for 24 months post-hatching, were bred at the Engineering Center of Polyploid Fish Breeding, National Education Ministry, Changsha, Hunan, China. The broodstock were sourced from the Yangtze River (30°25'56" N, 114°50'32" E). The parents of *Gobiocypris rarus* (NCBI:txid143606) were obtained from the Liu Sha River, Sichuan Province (coordinates 29°19'31" N, 10240'38" E). We also collected one individual of each fish species (*Cirrhinus molitorella* (NCBI:txid172907), *Pseudorasbora parva* (NCBI:txid51549), *Xenocypris davidi* (NCBI:txid291826), *Elopichthys bambusa* (NCBI:txid238031), and *Ctenopharyngodon idella* (NCBI:txid7959)) from Dongting Lake, Hunan, China (coordinates 29°15'8" N, 112°50'24" E). These individuals were deeply anesthetized with 300 mg/L tricaine methanesulfonate (Sigma-Aldrich, St. Louis, MO, USA) for 10 min (20°C) in a separation tank. After confirming their deaths, the muscle, brain, liver, intestine, kidney, and testis of all samples were collected after dissection.

DNA isolation and whole genome sequencing

High-quality and high-molecular-weight genomic DNA was isolated from muscle based on the DNA extraction methods. The purification was performed using the QIAGEN® Genomic Kit based on the standard operating procedure. The degradation and contamination of the extracted DNA were detected using 1% agarose gels. Then, DNA purity was determined using the NanoDrop™ One UV-Vis spectrophotometer (Thermo Fisher Scientific, USA) with 260/280 and 260/230 ratios. DNA concentration was measured by the Qubit® 4.0 Fluorometer (Invitrogen, USA).

After quality checking, the genomic DNA of *C. alburnus* and *M. amblycephala* was randomly sheared using Megaruptor (Diagenode, Denville, NJ, USA). DNA was size-selected using a SPRI bead protocol. The purity of the extracted DNA was determined using a Nanodrop spectrophotometer (Thermo Fisher Scientific). All procedures were carried out at room temperature. Large DNA fragments were separated using BluePippin DNA Size Selection System. DNA damage repair and end-repair were performed. Barcoded overhang hairpin adapters were ligated to the fragment ends. The connection reaction was performed using Ligation Sequencing Kit (Oxford Nanopore, SQK-LSK108). A constructed DNA library was quantified using Qubit. Lastly, sequencing was performed using Nanopore Sequencing.

The genomic DNA of *C. alburnus* and *M. amblycephala* was utilized for whole-genome re-sequencing. First, high-quality DNA samples were used to prepare single-stranded circular libraries. Subsequently, the circular libraries were transformed into DNA nanoballs (DNBs), which are spherical structures containing millions of copies of the circular DNA templates. Once the DNBs were formed, they were loaded onto patterned nanoarrays. Following the loading of DNBs onto the nanoarrays, combinatorial probe anchor synthesis sequencing was conducted. Finally, DNBSEQ-T7 sequencing was performed using a paired-end approach (150 bp × 2) in accordance with the standard protocol [27].

Fifteen μg of DNA for the six fishes (*C. idella*, *C. molitorella*, *P. parva*, *X. davidi*, *G. rarus*, and *E. bambusa*) was used for the preparation of SMRTbell target-size libraries, which were constructed using PacBio's standard protocol (Pacific Biosciences, CA, USA) with 15 kb preparation solutions. The main steps for library preparation are listed below: 1) The genomic DNA was sheared using g-TUBEs (Covaris, USA); 2) an A-tailing reaction was used to form an overhang; 3) the fragments were ligated with the hairpin adaptor using the SMRTbell Express Template Prep Kit 2.1 (Pacific Biosciences); 4) the library was treated with nuclease and purified using AMPure PB Beads; and 6) the SMRTbell library was purified using PB beads. The high-quality library was checked for fragment size using the Agilent 2100 Bioanalyzer (Agilent Technologies, USA). Sequencing Primer V2 and

Sequel II Binding Kit 2.1 were used for PacBio Sequel II sequencing.

Genome assembly and chromosomal organization

The adapter and low-quality bases of the two species were filtered before assembly using Fastp (RRID:SCR_016962) (v. 0.21.0) [28]. All clean reads of *C. alburnus* and *M. amblycephala* were used for genome assembly using Nextdenovo (RRID:SCR_025033) (v. 2.3.0) [29]. The parameters "random_round = 20, minimap2_options_cns = -x ava-ont -t 40 -k17 -w17, and nextgraph_options = -a 0" were used in genome assembly. The base errors (SNV/Indel) in the genome generated were fixed using Nextpolish (RRID:SCR_025232) (v. 1.3.0) [30]. For the six fishes (*C. idella, C. molitorella, P. parva, X. davidi, G. rarus, and E. bambusa*), the HIFI data was used for genome assembly using hifiasm (0.15.4-r347) software (RRID:SCR_021069) [31].

Hi-C libraries of *C. alburnus* and *M. amblycephala* were created from muscle cells. Briefly, cells were fixed with formaldehyde and lysed, and the cross-linked DNA was digested with MobI. Sticky ends were biotinylated and proximity ligated to form chimeric junctions that were enriched for and then physically sheared to a size of 300–700 bp, as illustrated in Rao et al. [32]. Chimeric fragments representing the original cross-linked long-distance physical interactions were then processed into paired-end sequencing libraries. The clean reads of Hi-C were obtained from trimming adapter sequences and low-quality pair-end reads, which were truncated at the putative Hi-C junctions, and then the resulting trimmed reads were aligned to the assembly results with BWA (RRID:SCR_010910) (v. 0.7.17) [33]. Invalid read pairs, including Dangling-End and Self-cycle, Re-ligation, and Dumped products, were filtered by HiC-Pro (RRID:SCR_017643) (v. 2.8.1) [34]. They were used for the correction of scaffolds and the clustering, ordering, and orientation of scaffolds onto chromosomes by LACHESIS (release: 2017-12-21) [35]. After this step, placement and orientation errors exhibiting obvious discrete chromatin interaction patterns were manually adjusted.

Gene prediction and annotation

For protein-coding gene prediction in the genomes of *C. alburnus* and *M. amblycephala*, we employed three integrated methods: *de novo* prediction, homology search, and cDNA-based prediction (muscle, brain, liver, intestine, kidney, and testis). *De novo* gene models were predicted using Augustus (RRID:SCR_008417) (v. 3.4.0) [36] with default parameters. In the homolog-based analysis, protein genes from five species (*C. carpio*: GCF_018340385.1, *C. auratus*: GCF_003368295.1, *O. macrolepis*: GCA_012432095.1, *P. tetrazona*: GCF_018831695.1, and *D. rerio*: GCF_000002035.6) obtained from NCBI were used to predict gene regions using GeneWise (v. 2.4.1) with default parameters. The cDNA-based approaches involved using Hisat2 (RRID:SCR_015530) (v. 2.1.0) [37] and TransDecoder (RRID:SCR_017647) (v. 5.5.0,) software to predict open reading frames (ORFs). Subsequently, we integrated the results of genome annotation using GETA (v. 2.5.7). Gene functional predictions were assigned using Blast-2.11.0+ against public databases, including Swiss-Prot, the Non-Redundant Protein Sequence Database (NR), the KOG database, KO annotations were conducted with Kofamscan software, and motifs and domains were predicted using Hmmer [38] software against the PFAM database.

To identify repetitive sequences, we utilized both *de novo*-based and homology-based methods. First, LTR_FINDER_parallel (RRID:SCR_018969) (v. 1.1) [39], LTRharvest (GenomeTools, v. 1.6.1) [40], LTR_retriever (RRID:SCR_017623) (v. 2.9.0) [41], and RepeatModeler (RRID:SCR_015027) (v. 2.0.1) software were employed to build a *de novo* repeat library, which was then merged with the Repbase database. RepeatMasker (RRID:SCR_012954) was subsequently used to predict repeat sequences using the new repeat library database. Tandem repeats were detected using Tandem Repeats Finder (TRF). For tRNA identification, we used tRNAscan-SE (RRID:SCR_008637) (v 2.0.7) [42], while rRNA was annotated using Blastn (RRID:SCR_001598) (BLAST v. 2.2.26, e-value: $1e^{-5}$) against the human rRNA sequence from the Rfam database. The snRNA and miRNA were searched using the Rfam database and the Infernal

(RRID:SCR_011809) (v. 1.0.2) software.

Comparative phylogenomics

For the phylogeny analyses, we performed multiple whole-genome alignments (WGAs) for 17 (non-polyploid species) and 24 (including 7 polyploid species) species using cactus (v. 2.1.1), respectively [43]. The WGAs were utilized to construct a phylogenetic tree with *Beaufortia kweichowensis* as the root. To facilitate the analysis, syntenic blocks were concatenated into 10-kb windows. Subsequently, a file containing 6 Mb (17 species) and 4.75 Mb (24 species) sequences for each genome was generated, respectively. To build the maximum likelihood tree, we employed RAxML (RRID:SCR_006086) (v. 8.2.12) [44] with the following parameters: -p 12345 -# 100 -m GTRGAMMA -s all.phylip -o B.kweichowensis -f a -x 12345 -k -n tree -T 10. The coalescent species tree estimations were performed using Astral (RRID:SCR_001886) (v. 5.15.5) [45]. For estimating divergence times, we used the MCMCTree (RRID:SCR_025348) in PAML (4.9j) [46] with four fossil calibration time points. The conserved scores of the 17 non-polyploid species were estimated using the phastCons tool from the phast packages [47]. VCF files for each species were generated by aligning whole genomes to the zebrafish genome using Chen's methods [48]. To investigate gene flow, we exclusively analyzed the 17 non-polyploid genomes from non-inbred populations using the ABBA-BABA test implemented in the Dsuite (0.4 r38) software [49] with the D-statistic method. The results were visualized using the Fbranch and dtools.py programs in Dsuite. To ensure a sufficient number of informative sites for analysis within each examined window, we employed a Python script named "ABBABABAwindows.py" to detect window D values. We used a window size of 20 kb with a step size of 10 kb, implemented through the script's parameters "-w 20000 -m 100 -s 10000".

RNA isolation and mRNA-seq

Total RNA from the brain, liver, intestine, muscle, kidney, and testis organs of three individuals

(*C. alburnus* and *M. amblycephala*) was isolated and purified according to the TRIzol extraction method, respectively [50]. The RNA concentration was measured using NanoDrop technology. Total RNA samples were treated with DNase I (Invitrogen) to remove any contaminating genomic DNA. The purified RNA was quantified using a 2100 Bioanalyzer system (Agilent, Santa Clara, CA, USA). The isolated mRNA was fragmented with a fragmentation buffer. The resulting short fragments were reverse transcribed and amplified to produce cDNA. The transcriptome data of 36 samples (three biological replicates) was obtained using DNA nanoball DNBSEQ-T7 (RRID:SCR_017981) technology according to the standard method [51]. The main steps were listed below: 1) single-stranded circular libraries were prepared using MGI Library Prep Kits; 2) after the hybridization of a DNA anchor, a fluorescent probe is attached to the DNA nanoball using combinatorial probe anchor sequencing chemistry; 3) the high-resolution imaging system captures the fluorescent signal; 4) after digital processing of the optical signal, the sequencer generates high-quality and accurate sequencing information. Low-quality bases and adapters were trimmed out using SOAPnuke with the thresholds "-n 0.01 -l 20 -q 0.4 -A 0.25 --cutAdaptor -Q 2 -G --polyX 50 --minLen 150" [52]. The high-quality reads were used in the next analyses.

Gene expression profiling

All clean reads of *M. amblycephala* and *C. alburnus* were mapped to their corresponding reference genomes using HISAT2 (v. 2.1.0) [37] with default parameters. Then, the mapped files were handled with SAMtools/BCFtools (RRID:SCR_005227) (v. 1.10) [53], while the unique mapped reads were obtained using htseq-count (RRID:SCR_011867) (v. 0.12.4) [54]. The gene expression value of mRNA-seq was normalized and calculated based on the transcripts per million (TPM) values. Genes with mapped reads < 5 in each sample were not used in our next analyses. Differential expression (DE) analysis was performed using Deseq2 (RRID:SCR_015687)[55] of R package with the thresholds: p-value < 0.001 and Padj < 0.001. Organ-specific genes (OSGs) were identified using

the following criteria: Gene expression in the target tissue or organ differs significantly from that in the other five tissues and organs. Orphan genes (OGs) are detected based on the thresholds of BLASTx with an e-value of $1e^{-5}$ and tBLASTx with an e-value of $1e^{-5}$. The sequences with no BLAST result in the public database were considered potential OGs. Then, the expressed OGs (TPM > 10) were considered OGs in the corresponding tissue or organ. GO analysis was performed with a significance threshold (false discovery rate of Benjamini–Hochberg method < 0.05).

Diversifying selection analysis

17,337 orthologous gene pairs (OGPs) between *M. amblycephala* and *C. alburnus* were obtained using the all-against-all reciprocal BLASTP (v. 2.8.1) with an e-value of $1e^{-6}$ based on protein sequences (sequence alignment > 70%). Then, transcripts that were shorter than 300 bp were discarded from OGPs. OGPs in the comparison of *M. amblycephala* and zebrafish and the comparison of *M. amblycephala* and zebrafish were obtained based on the above thresholds. We performed DE analyses on the OGPs between *M. amblycephala* and *C. alburnus* in the six tissues and organs. Differential expression (DE) analysis was performed using Deseq2 [55] with the thresholds: p-value < 0.001 and Padj < 0.001. The Ks and Ka/Ks values were calculated based on the below analysis process: 1) ParaAT2.0 and muscle software were used in sequence alignment of OGPs with the default parameters; 2) kaks_calculator3.0 program was used to calculate Ks and Ka/Ks values using maximum likelihood method [56]. The threshold of a p-value < 0.05 was used in our analyses.

Measurement of enzymatic content

Equal amounts of liver (0.1 g) from *M. amblycephala* and *C. alburnus* (10 individuals in each species) were collected from Engineering Center of Polyploid Fish Breeding of National Education Ministry in Hunan, China. Then, homogenates were used to determine the activity of trypsin and lipase. Trypsin assay kit (A080-2-2) and lipase assay kit (A054-1-1) were purchased from Nanjing Jiancheng

Bioengineering Institute (Jiangsu, China), and the experimental protocols followed the manufacturer's instructions. The significant difference was performed using Student's *t*-test.

Hematoxylin and eosin staining

A 10 mm-thick section of skeletal muscle from four fish species, *M. amblycephala*, *C. alburnus*, *C. idella*, and *E. bambusa*, was dissected from the dorsum region. A 10 mm-thick section of intestine was dissected from the abdominal cavity of each of the four fish species after removing food residues. The tissue sections were then fixed in Bouin's solution for 24 hours. After fixation, the tissues were washed with distilled water for 4 hours at room temperature. The fixed tissues were then dehydrated using a series of alcohol concentrations (e.g., 70%, 80%, 90%, and 100%) and embedded in paraffin blocks. The paraffin-embedded tissue blocks were sectioned into 10 μm-thick slices using a microtome. The tissue sections were processed for hematoxylin and eosin (HE) staining according to the manufacturer's instructions using an HE staining kit. Digital images of the stained sections were captured using a microscope (DX8; Olympus, Tokyo, Japan). The samples obtained from three individuals were performed for each hybrid variety, and quantitative data on HE staining were collected from them.

Results

Genome assembly

A total of eight species of cyprinid fishes from East Asia were sequenced using PacBio HiFi or Oxford Nanopore technology, resulting in over 602.21 Gb of raw data (Supplemental File 1: Table S1). *De novo* assembled genomes were obtained with contig N50 ranging from 7.57 Mb to 38.12 Mb. Chromosome-scale genomes were assembled for blunt snout bream *(Megalobrama amblycephala*, BSB) and topmouth culter (*Culter alburnus*, TC) using 204.8 Gb Hi-C data. The resulting assemblies exhibited scaffold N50 values of 42.91 Mb and 39.60 Mb, respectively (Table 1 and Supplemental File

1: Tables S1-S2). Assembly quality was assessed using BUSCO, scoring between 94.5% and 98.7% (Supplemental File 1: Table S3). We significantly improved the genome assemblies for both *M. amblycephala* (contig N50 increased from 2.4 Mb to 15.42 Mb [57]) and *C. alburnus* (contig N50 increased from 17.8 Mb to 18.55 Mb [58]). High-quality genome data for *Cirrhinus molitorella*, *Pseudorasbora parva*, and *Xenocypris davidi* were presented for the first time. Through a combination of *de novo*, protein homology, and cDNA-based prediction, we annotated 26,550 and 27,303 protein-coding genes for *M. amblycephala* and *C. alburnus*, respectively (Supplemental File 1: Tables S4-S5). Repetitive elements comprised 51.81% (568.18 Mb) and 50.49% (544.26 Mb) of the assemblies for *M. amblycephala* and *C. alburnus*, respectively (Supplemental File 1: Table S6). Non-coding RNA was predicted in 8.76% and 7.21% of the genome assemblies for *M. amblycephala* and *C. alburnus*, respectively (Supplemental File 1: Table S7). Furthermore, we obtained high-quality assembled genomes for 15 cyprinid fishes (with an average scaffold N50 of 33.37 Mb) and *Beaufortia kweichowensis* from public databases (Supplemental File 1: Table S8). These 23 cyprinid fishes represent seven non-polyploid subfamilies (*Danioninae*, *Xenocyprinae*, *Gobioninae*, *Leuciscinae*, *Cultrinae*, *Labeoninae*, and *Hypophthalmichthyinae*) and three polyploid subfamilies (*Schizothoracinae*, *Barbinae*, and *Cyprininae*), with genome sizes ranging from 0.86 Gb to 1.90 Gb and chromosome numbers varying widely from 48 to 150 (Table 1, Supplemental File 1: Tables S1-S2, and S8).

Phylogenomic analyses and introgression

To investigate the evolutionary relationships among extant cyprinids, we analyzed 24 genomes with butterfly hillstream loach (*B. kweichowensis*) as an outgroup (Fig. 1a and Supplemental File 2: Fig. S1). Our results showed that *Gobiocypris rarus* belongs to the subfamily *Gobioninae*, even though from a morphological perspective, it appears similar to zebrafish (which belongs to the subfamily *Danioninae* of Cyprinidae) [59]. Molecular clock analysis with fossil calibration indicated their divergence time ranging from 41.5-61.3 million years ago (MYA) (Fig. 1a and Supplemental File 1:

Table S9). Phylogenetic trees reconstructed the evolutionary history of subfamily *Leuciscinae*, showing that one group (including *Leuciscus idus, Abramis brama,* and *Rutilus rutilus*) diverged from another group (*Ctenopharyngodon idella* and *Elopichthys bambusa*) ranging from 23.8-35.1 MYA. This divergence occurred earlier than the divergence times observed among other subfamilies (*Cultrinae*, *Gobioninae, Xenocyprinae*, and *Hypophthalmichthyinae*) (Fig. 1a and Supplemental File 1: Table S9). Furthermore, phylogenomics analysis provided evidence regarding the divergence of common ancestors of extant cyprinids, which ranged from 81.9-100.0 MYA (Fig. 1a and Supplemental File 1: Table S9). The ancestor of *Danio rerio* (subfamily: *Danioninae*) diverged early in the evolution of extant cyprinids, while the ancestor of *Cirrhinus molitorella* and *Labeo rohita* (subfamily: *Labeoninae*) diverged between 36.5-53.6 MYA (Fig. 1a and Supplemental File 1: Table S9). These findings will assist us in understanding the evolutionary process of fish and constructing more reasonable classification relationships within the cyprinids, with the support of data from fields such as fossils, monsoons, and geography [60, 61]

Previous studies have reported phylogenetic discordance across genome regions in non-polyploid cyprinid fishes from the East Asian region. This discordance has been attributed to incomplete lineage sorting, introgression, and the fish's demographic history [62]. To investigate this, we conducted gene flow analysis and observed pervasive introgression among the 17 non-polyploid cyprinid fishes (f4-ratio > 0.0006, Z-score > 3, and p-value < 0.05) (Fig. 1b). The ABBA-BABA tests [49, 63] revealed strong gene flow events among subfamily *Gobioninae*, including *Paracanthobrama guichenoti* and *Gobiocypris rarus* (f4-ratio = 0.1, Z-score = 129 and p-value < 0.001), *G. rarus* and *Gobio gobio* (f4-ratio = 0.1, Z-score = 120 and p-value < 0.001), and *P. guichenoti* and *G. gobio* (f4-ratio = 0.1, Z-score = 96 and p-value < 0.001) (Fig. 1b and Supplemental File 1: Table S10). Notably, distinct gene flow signals (f4-ratio > 0.05) between two species were predominantly detected in 22 groups involving 12 species and 7 subfamilies (*Cultrinae*, *Danioninae*, *Gobioninae*, *Hypophthalmichthyinae*, *Leuciscinae_1*, *Leuciscinae_2*, and *Xenocyprinae*) (red dotted line in Fig. 1b and Supplemental File 1:

Table S10). Recent studies utilizing mitochondrial genomes and *de novo* nuclear genomes have also highlighted frequent gene flow events during the radiation of cyprinid fishes [61]. Our findings, based on high-quality genome assemblies, support the hypothesis that gene flow is the primary driver of the observed phylogenetic incongruence among non-polyploid cyprinid fishes.

Conservation of the reproductive system in speciation

The frequent occurrence of gene flow events between cyprinid fishes, including *M. amblycephala* and *C. alburnus*, suggests incomplete reproductive isolation as a potential contributing factor to their speciation in the East Asian region. To investigate the underlying genetic mechanisms, we focused on comparing the genomes of these two species, which belong to different genera within the *Cultrinae* subfamily but share overlapping habitats in the middle and lower reaches of the Yangtze River Basin (Fig. 2a). While laboratory experiments have indicated some degree of postzygotic isolation, no natural hybrid populations have been identified in the wild [64]. A conserved synteny analysis revealed a high degree of gene conservation between *M. amblycephala* and *C. alburnus* (Supplemental File 2: Figs. S2-S3), making them a suitable model for studying the genetic basis of their speciation.

To investigate the genetic differences between *M. amblycephala* and *C. alburnus*, we conducted comparative gene expression analyses in six tissues: brain, liver, intestine, muscle, kidney, and testis (Supplemental File 1: Tables S11-S12). Our findings revealed that the testis exhibited a higher proportion of organ-specific gene (OSG), accounting for 6.67% in *C. alburnus* and 7.95% in *M. amblycephala*, compared to the intestine and kidney (Fig. 2b). We focused on orphan genes (OGs) [65] and identified a greater number of OGs in the testis of both species, suggesting rapid divergence in this organ (Fig. 2c and Supplemental File 1: Table S13). However, the number of differentially expressed genes (DEGs) between the two species was lower in the testis than in the three tissues (Fig. 2d, Supplemental File 2: Fig. S4 and Supplemental File 1: Tables S14-S16). To explore functional divergence, we calculated Ka/Ks values for OSGs and found that the testis exhibited lower Ka/Ks

values compared to the intestine (*t*-test: $p = 0.04$) but higher values compared to the three tissues (liver, brain, and muscle, *t*-test: $p < 0.001$) (Fig. 2e). Similar patterns were observed for DEGs and the shared genes (OSGs and DEGs) (Supplemental File 2: Fig. S5). Finally, to assess the degree of sequence conservation [47], we calculated phastCons scores of OSGs and observed the highest median value in the testis, which was higher than in the intestine, kidney, and muscle (*t*-test: $p < 0.05$) (Fig. 2f). A similar phenomenon was noted when analyzing DEGs using phastCons scores (Supplemental File 2: Fig. S6). *M. amblycephala* and *C. alburnus* exhibited lower genetic variation in their testes compared to their intestines.

Rapid evolution of digestive system in speciation

The diverse digestive systems of cyprinid fishes allow them to adapt to various food sources, including plankton, aquatic plants, and benthic organisms, contributing to their ecological niche differentiation [66]. Differential expression analyses revealed that the intestine of *C. alburnus* had the lowest number of OSGs (752, 4.15%), while *M. amblycephala*'s intestine had the second lowest (942, 5.95%) (Fig. 2b). The number of OGs in the intestine (0.59% in *C. alburnus* and 0.51% in *M. amblycephala*) was lower compared to the testis, brain, and kidney (Fig. 2c). The study demonstrates that the genetic makeup of the intestine exhibits a higher degree of conservation compared to the other tissues and organs. However, rapid genetic divergence between the herbivorous *M. amblycephala* and the carnivorous *C. alburnus* was observed in their intestines. For instance, the highest number of DEGs between the two species was detected in their intestines (Fig. 2d and Supplemental File 1: Tables S14-S15). Moreover, the Ka/Ks values of OSGs were higher in the intestine compared to the brain, kidney, muscle, and testis (Fig. 2e). Lastly, the phastCons scores of OSGs in the intestine were lower than those in the brain, liver, muscle, and testis, although they were higher than in the kidney (Fig. 2f). Similar trends were observed in the phastCons scores of DEGs (Supplemental File 2: Fig. S6). Our findings provide preliminary evidence suggesting a potential rapid divergence in genetic diversity

within the digestive organs of *M. amblycephala* and *C. alburnus*.

To investigate the genetic basis of diet divergence between herbivorous *M. amblycephala* and carnivorous *C. alburnus*, we conducted a functional analysis of their DEGs in the intestine. These genes associated with digestive enzymes were enriched for hydrolyzing O-glycosyl compounds (GO: 0004553) and peptidase activity (GO: 0008233) in terms of Molecular Function annotation, while carbohydrate metabolic process (GO: 0005975) and lipid catabolic process (GO: 0016042) were enriched for Biological Process annotation (Supplemental File 2: Fig. S7). Among these genes, *dpp2*, *ctrl*, *psb7*, and *ppce* were identified as potential genes involved in peptidase activity, exhibiting higher expression in the digestive organs (liver and intestine) of *C. alburnus* compared to *M. amblycephala* (Fig. 3a). After detecting the enzyme activities of trypsin and lipase in digestive organs, we found that the enzyme activities were higher in the carnivorous *C. alburnus* compared to the herbivorous *M. amblycephala* (Fig. 3b). We conducted analyses on positively selected genes (PSGs) (Ka/Ks > 1) between the two species and identified 30 of them that belong to OSGs in the six tissues and organs (Supplemental File 1: Table S17). Among the share genes of PSGs and OSGs in the intestine, *caspbl* and *vsig* were found to be associated with peptidase activity (GO: 0008233), apoptosis, and immune responses, which are closely related to the types of digested food (Supplemental File 2: Fig. S8) [67, 68]. These findings suggest that the observed genetic diversity may be related to adaptations in digestive enzyme secretion, reflecting potential dietary adjustments.

Gene flow and its potential impact on feeding habits

Frequent gene flow events were observed among cyprinid fishes, including *M. amblycephala*, *C. alburnus*, *C. idella*, and *E. bambusa*. Among these, significant gene flow was detected between carnivorous *C. alburnus* (subfamily *Cultrinae*) and *E. bambusa* (subfamily *Leuciscinae*) (Z-score > 45.8, f4–ratio = 0.039, and p-value < 0.001). Additionally, gene flow event was identified between the

herbivorous *M. amblycephala* (subfamily Cultrinae) and *C. idella* (subfamily *Leuciscinae*) (Z-score > 38.3, f4–ratio = 0.044, and *p*-value < 0.001) (Fig. 1b and Supplemental File 1: Table S10). The overlapping habitats of these four species were primarily distributed in the eastern region of China (Fig. 4a).

To investigate the effects of gene flow events on diet diversity, we analyzed the 117 introgressed genomic regions (window size: 20 kb) between the two carnivorous fish species, which were associated with 69 genes (Supplemental File 1: Table S18). Similarly, the 102 introgressed regions (window size: 20 kb) between the two herbivorous fish species were associated with 68 genes (Supplemental File 1: Table S19). Among these genes, the top three molecular function annotations in both carnivores and herbivores were related to transcription regulator activity (GO: 0140110), DNA-binding transcription factor activity (GO: 0003700), and RNA polymerase II-specific activity (GO: 0000981) (Supplemental File 1: Tables S20-S21). Among these genes, 10 introgressed genes were shared between carnivores and herbivores (Fig. 4b). Moreover, 84 categories (38.36%) for biological processes and 16 categories (66.67%) for molecular functions were shared between carnivores and herbivores (FDR < 0.05, Fig. 4c and Supplemental File 2: Figs. S9-S10). These shared introgressed genes were associated with animal organ development, including skeletal muscle organ development (GO: 0060538; *sox6* and *ttn.2*) and head development (GO: 0060322; *zfhx3*, *tcf7l2*, and *meis1b*) (Supplemental File 2: Fig. S11). *Zbtb16a* (linked to osteogenic differentiation [69]) exhibited the broadest expression pattern among all introgressed genes, being detected in five different tissues and organs (Fig. 4d). This suggests that *zbtb16a* may be a hotspot for introgression events between *Cultrinae* and *Leuciscinae*.

There were potential relationships between diet habits and organ development, including mouth and pharyngeal tooth morphologies, intestinal morphology, and skeletal muscle structure (Fig. 4e). Comparative analyses revealed that carnivorous fishes exhibited larger and superior mouths, longer and sharper teeth, shorter intestines, thinner intestine linings, and smaller cross-sectional areas in

skeletal muscle fibers compared to herbivorous fishes (Fig. 4). It is noteworthy that some of the introgressed genes have been experimentally validated in zebrafish to have functional associations with dietary traits. For instance, *tp53* and *tle3a* are implicated in intestinal morphology [70, 71], while *grin2bb* and *grin1a* are linked to food intake behavior [72, 73]. Additionally, *znf536*, *zfhx3*, *elavl4*, *hoxc11a*, *pik3r3b*, and *irf2bpl* have been identified as potential regulators of swimming behavior. These findings suggest that these introgressed genes may relate to organ development and feeding behavior may contribute to diet divergence for these fishes.

Discussion

The East Asian region, characterized by its unique topography, including the uplift of the Qinghai-Tibet Plateau, abundant rivers, and diverse climatic environments, is home to a multitude of freshwater fish species [61, 74, 75]. Cyprinids represent the largest and most diverse vertebrate group, with over 654 species, including 440 endemics in China [76]. Investigating the genetic mechanisms underlying their rapid speciation is vital for understanding evolutionary radiation in East Asian cyprinids. Our findings suggest that frequent gene flow events among cyprinid fishes have contributed to their rapid adaptive radiation, as evidenced by our analyses of seven non-polypoid subfamilies. This prompts the question: How does introgressive hybridization in East Asian cyprinids relate to rapid speciation?

Rapid evolutionary changes in the mammalian testis manifest at the molecular level, contributing to reproductive isolation. Comparative gene expression studies across various mammalian organs reveal that the testis exhibits the highest rates of evolutionary expression change [77, 78]. Therefore, comparative genomic analysis of *M. amblycephala* and *C. alburnus* revealed a lower degree of genetic divergence in the testis compared to the intestine. Additionally, laboratory experiments have demonstrated the production of fertile hybrids between various cyprinid species [13, 64], suggesting the possibility of incomplete reproductive isolation (RI) and the prevalence of introgressive hybridization in East Asian cyprinids. However, further research is needed to elucidate the specific

factors hindering and driving rapid speciation in these cyprinid fishes.

The complex and variable inland water ecosystem plays a crucial role in the adaptive evolution of fish [79, 80]. Among these factors, the diversity of food sources gradually influences the feeding habits of different populations, resulting in the adaptive evolution of their digestive and locomotion systems [81]. Our findings suggest that speciation in *M. amblycephala* and *C. alburnus* may have been driven by diet-dependent adaptations. Cyprinid fishes display significant diversity in behavior, habitat, geography, and morphology, including variations in feeding and digestive organs [61, 76]. Robust pharyngeal teeth and toothless jaws enables them to consume a wide range of foods [82, 83]. Our results reveal that rapid genetic divergence between *M. amblycephala* and *C. alburnus* occurs in the intestine. The variations in digestive enzyme secretion and digestive organs reflect their distinct feeding preferences and the effectiveness with which they metabolize different food types [84]. Considering the absence of post-zygotic isolation and the overlapping habitat between *M. amblycephala* and *C. alburnus* [64, 85], our results suggest that ecological differentiation driven by dietary differences may be an important factor leading to the rapid formation of these two species.

When post-zygotic reproductive isolation is no longer a significant barrier to gene flow among East Asian cyprinids, natural selection, including monsoon activities [61], and the uplift of the Qinghai-Tibet Plateau [75], can attenuate pre-zygotic isolation, providing opportunities for gene flow, thus promoting speciation in cyprinid fishes [86]. To adapt to diverse food supplies in different aquatic environments, feeding habits have diverged in the subfamilies *Cultrinae* (herbivorous *M. amblycephala* and carnivorous *C. alburnus*) and *Leuciscinae* (herbivorous *C. idella* and carnivorous *E. bambusa*). Does gene flow facilitate the divergence of diets for adaptive evolution? Our results demonstrate the introgression of genes associated with skeletal muscle and head development between fishes with the same diet. These changes play crucial roles in feeding and digestive efficiency. Fishes with the same diet in different subfamilies exhibit similar phenotypes involving the mouth, teeth, intestine, and muscle. These results suggest that coevolving interactions of diet habits occur in their

speciation through introgressive hybridization. However, further evidence is needed to establish a definitive association between dietary convergent evolution and gene flow.

Additional Files

Supplemental File 1. Table S1. Summary of whole genome sequencing in eight species. Table S2. Genome assembly of eight species. Table S3. Completeness of the eight assembled genomes. Table S4. Statistics of gene prediction. Table S5. Gene function annotation of *M. amblycephala* and *C. alburnus*. Table S6. Summary of repeat contents. Table S7. The summary of predicted non-coding RNA in *M. amblycephala* and *C. alburnus*. Table S8. Information of downloaded genomes. Table S9. Range of divergence time in Fig. 1. Table S10. D statistic on the species tree based on genome-wide single nucleotide polymorphisms (SNPs). The outgroup was fixed as *Beaufortia kweichowensis*. Table S11. Summary of transcriptome sequencing data. Table S12. Summary of transcriptome mapping data. Table S13. List of orphan genes (OGs) in *M. amblycephala* and *C. alburnus*. Table S14. Summary of differentially expressed genes (DEGs) between *M. amblycephala* and *C. alburnus* in six organs. Table S15. The gene number of orthologous gene pairs and differential expressed genes (DEGs) in six organs. Table S16. The summary of organ-specific genes (OSGs), positive selective genes (PSGs), and differential expressed genes (DEGs) in six organs. Table S17. Summary of expressed positive selective genes (PSGs) between *C. alburnus* (TC) and *M. amblycephala* (BSB) in the six organs. Table S18. Summary of gene flow between carnivorous *C. alburnus* and *E. bambusa*. Table S19. Summary of gene flow between herbivorous *M. amblycephala* and *C. idella*. Table S20. GO enrichment of introgressed genes between carnivorous *C. alburnus* and *E. bambusa*. Table S21. GO enrichment of introgressed genes between herbivorous *M. amblycephala* and *C. idella*.

Supplemental File 2. Fig. S1. Phylogenetic trees constructed using multiple whole-genome alignments of 17 (no polyploid species) and 24 (including 7 polyploid species) species with *Beaufortia kweichowensis* as the root, respectively. (A) Concatenation-based method for estimating a phylogenetic tree of 17 species with 10-kb length windows. (B) Coalescent method for estimating a phylogenetic tree of 17 species with 10-kb length windows. (C) Concatenation-based method for estimating a phylogenetic tree of 24 species with 10-kb length windows. (D) Coalescent method for

estimating a phylogenetic tree of 24 species with 10-kb length windows. (E) Species tree with estimated divergence time. Fig. S2. The Hi-C interaction heatmap of 24 linkage groups in the genomes of *M. amblycephala* and *C. alburnus*. Fig. S3. The collinearity analysis between *M. amblycephala* and *C. alburnus*. Twenty-four pairs of homologous chromosomes were determined based on 17,337 orthologous gene pairs. Fig. S4. The differential expression between *M. amblycephala* and *C. alburnus* in six organs. Up-regulated genes in *M. amblycephala* are marked in blue, while the up-regulated genes in *C. alburnus* are marked in red. Fig. S5. The distribution of Ka/Ks values in the six organs. (A) The Ka/Ks values of DEGs. (B) The Ka/Ks values of share genes between DEGs and OSGs. The median value is indicated by a black dot, and the gene number is provided below each organ name. In the t-test, "*" represents 0.01 < p-value ≤ 0.05, "**" represents 0.001 < p-value ≤ 0.01, "***" represents p-value ≤ 0.001. Fig. S6. Conserved scores of DEGs (*M. amblycephala* vs. *C. alburnus*) in the six organs. "*" represents 0.01 < p-value ≤ 0.05, "**" represents 0.001 < p-value ≤ 0.01, "***" represents p-value ≤ 0.001. Fig. S7. The DEGs (*M. amblycephala* vs. *C. alburnus*) associated with diet habit. (A) The heatmap of the DEGs in the intestine. The hydrolyzing O-glycosyl compounds and peptidase activity in Molecular Function, as well as carbohydrate metabolic process and lipid catabolic process in Biological Process. (B) The gene distribution of DEGs in the intestine. Fig. S8. The alignment of two positively selected genes (PSGs) in intestine. Fig. S9. The GO terms of introgressed genes in the carnivorous (*C. alburnus* and *E. bambusa*) and herbivorous *(M. amblycephala* and *C. idella*) fishes. Fig. S10. The distribution of enriched functional categories (FDR < 0.05) in Biological Process and Molecular Function for the introgressed genes. Fig. S11. Heatmap exhibiting the expression of introgressed genes in the carnivorous (*C. alburnus* and *E. bambusa*) and herbivorous (*M. amblycephala* and *C. idella*) fishes.

Abbreviations

BSB: blunt snout bream; TC: topmouth culter; RI: reproductive isolation; TRF: tandem repeats finder;

TPM: transcripts per million; DE: differential expression; OGs: orphan genes; OGPs: orthologous gene pairs; MYA: million years ago; OSG: organ-specific gene; PSGs: positively selected genes; DEGs: differentially expressed genes.

Acknowledgements

We thank Min Xie at Hunan Fisheries Science Institute for their invaluable assistance in collecting the fish samples.

Author contributions

S.J.L., L.R., and X.L.T. wrote the manuscript. S.J.L., D.D.W., L.R., and Q.Z.L. modified the manuscript and designed the study. X.L.T., L.R., and M.X.L. carried out bioinformatics analyses. J.L.C., X.G., H.Z., Y.K.T., Y.Y.Z., M.D.L., W.H.L., C.W., and J.W. extracted the raw material. All authors read and approved the final manuscript.

Funding

This research was supported by National Natural Science Foundation of China (32293252, 32341057, and U19A2040), Hunan Provincial Natural Science Foundation (2022JJ10035), Huxiang Young Talent Project of China (2021RC3093), National Key Research and Development Plan Program (2023YFD2401602), Laboratory of Lingnan Modern Agriculture Project (NT2021008), Special Funds for Construction of Innovative Provinces in Hunan Province (2021NK1010), earmarked fund for China Agriculture Research System (CARS-45), and 111 Project (D20007).

Competing Interests

The authors have declared that no competing interests exist.

Data Availability

Genomic sequencing data obtained from PacBio HiFi, Oxford Nanopore, and DNBSEQ-T7 technologies, as well as Hi-C data, have been submitted to the National Center for Biotechnology Information (NCBI) (accession numbers: SRR26190421-SRR26190427, SRR26139312-SRR26139313, SRR26139214-SRR26139215, and SRR26319599-SRR26319600). The assembled genome and annotation files of eight cyprinid fishes have been deposited on Figshare [88] and the National Genomics Data Center (NGDC) (accession numbers: GWHDOEU00000000, GWHDOEX00000000, GWHDOEV00000000, GWHDOEW00000000, GWHDOEB00000000, GWHDOEC00000000, GWHDOES00000000, and GWHDOET00000000). The raw reads of the mRNA-seq data have been submitted to NGDC (accession number: subCRA017373) and NCBI (accession numbers: SRR26087118-SRR26087153). All additional supporting data are available in the *GigaScience* repository, GigaDB [89-97].

Animal ethics declarations

All procedures performed on animals were approved by the academic committee at Hunan Normal University, Hunan, China (approval number: 2020C034).

References

1. Froese R and Pauly D. FishBase. World Wide Web electronic publication. In: 2014.
2. Nelson J, Grande T and Wilson M. Fishes of the World, Fifth Edition. 2016.
3. Yang L, Sado T, Vincent Hirt M, Pasco-Viel E, Arunachalam M, Li JB, et al. Phylogeny and polyploidy: Resolving the classification of cyprinine fishes (Teleostei: Cypriniformes). Mol Phylogenet Evol. 2015;85:97-116. doi:doi: 10.1016/j.ympev.2015.01.014.
4. He FZ, Zarfl C, Bremerich V, David JN, Hogan Z, Kalinkat G, et al. The global decline of freshwater megafauna. Global Change Biol. 2019;25 11:3883-92.
5. Jacquemin SJ and Pyron M. A century of morphological variation in Cyprinidae fishes. BMC Ecology. 2016;16 1:48. doi:10.1186/s12898-016-0104-x.
6. German DP, Nagle BC, Villeda JM, Ruiz AM, Thomson AW, Contreras Balderas S, et al. Evolution of herbivory in a carnivorous clade of minnows (teleostei: cyprinidae): effects on gut size and digestive physiology. Physiological and biochemical zoology: PBZ. 2010;83 1:1-18. doi:10.1086/648510.
7. Yue PQ, Shan XH and Lin RD. Fauna sinica, osteichthyes, cypriniformes III. Science, Beijing (in Chinese). 2000.
8. Haenen O, Way K, Gorgoglione B, Ito T, Paley R, Bigarré L, et al. Novel viral infections threatening Cyprinid fish. Bulletin of the European Association of Fish Pathologists. 2016;36 1:11-23.
9. Brauer CJ, Sandoval-Castillo J, Gates K, Hammer MP, Unmack PJ, Bernatchez L, et al. Natural hybridization reduces vulnerability to climate change. Nat Clim Change. 2023;13:282–9. doi:10.1038/s41558-022-01585-1.
10. Mallet J. Hybridization as an invasion of the genome. Trends Ecol Evol. 2005;20 5:229-37. doi:10.1016/j.tree.2005.02.010.
11. Schumer M, Powell DL, Delclós PJ, Squire M, Cui R, Andolfatto P, et al. Assortative mating and persistent reproductive isolation in hybrids. Proc Natl Acad Sci U S A. 2017;114 41:10936-41. doi:doi:10.1073/pnas.1711238114.
12. Birkhead TR and Brillard JP. Reproductive isolation in birds: postcopulatory prezygotic barriers. Trends Ecol Evol. 2007;22 5:266-72. doi:10.1016/j.tree.2007.02.004.
13. Wang S, Tang CC, Tao M, Qin QB, Zhang C, Luo KK, et al. Establishment and application of distant hybridization technology in fish. Sci China Life Sci. 2019;62 1:22-45. doi:10.1007/s11427-018-9408-x.
14. Su GH, Logez M, Xu J, Tao SG, Villéger S and Brosse S. Human impacts on global freshwater fish biodiversity. Science. 2021;371 6531:835-8.
15. Dias MS, Oberdorff T, Hugueny B, Leprieur F, Jézéquel C, Cornu JF, et al. Global imprint of historical connectivity on freshwater fish biodiversity. Ecology Letters. 2014;17 9:1130-40.
16. Seehausen O. Hybridization and adaptive radiation. Trends Ecol Evol. 2004;19 4:198-207. doi:10.1016/j.tree.2004.01.003.
17. Geiger MF, Herder F, Monaghan MT, Almada V, Barbieri R, Bariche M, et al. Spatial heterogeneity in the Mediterranean Biodiversity Hotspot affects barcoding accuracy of its freshwater fishes. Mol Ecol Resour. 2014;14 6:1210-21.
18. Costedoat C, Pech N, Salducci M-D, Chappaz R and Gilles A. Evolution of mosaic hybrid zone between invasive and endemic species of Cyprinidae through space and time. Biological Journal of the Linnean Society. 2005;85 2:135-55.
19. Broughton RE, Vedala KC, Crowl TM and Ritterhouse LL. Current and historical hybridization with differential introgression among three species of cyprinid fishes (genus Cyprinella). Genetica. 2011;139:699-707.
20. Pereira CSA, Aboim MA, Ráb P and Collares-Pereira MJ. Introgressive hybridization as a promoter of genome reshuffling in natural homoploid fish hybrids (Cyprinidae, Leuciscinae). Heredity. 2014;112 3:343-50.

doi:10.1038/hdy.2013.110.

21. Aboim M, Mavárez J, Bernatchez L and Coelho M. Introgressive hybridization between two Iberian endemic cyprinid fish: a comparison between two independent hybrid zones. Journal of Evolutionary Biology. 2010;23 4:817-28.

22. Rønnestad I, Yufera M, Ueberschär B, Ribeiro L, Sæle Ø and Boglione C. Feeding behaviour and digestive physiology in larval fish: current knowledge, and gaps and bottlenecks in research. Reviews in Aquaculture. 2013;5:S59-S98.

23. Kuang ZR, Li F, Duan QJ, Tian CC, Nevo E and Li KX. Host diet shapes functionally differentiated gut microbiomes in sympatric speciation of blind mole rats in Upper Galilee, Israel. Front Microbiol. 2022;13:1062763. doi:10.3389/fmicb.2022.1062763.

24. Chen HY, Li CQ, Liu T, Chen SY and Xiao H. A Metagenomic Study of Intestinal Microbial Diversity in Relation to Feeding Habits of Surface and Cave-Dwelling Sinocyclocheilus Species. Microb Ecol. 2020;79 2:299-311. doi:10.1007/s00248-019-01409-4.

25. Sibbing F. Food capture and oral processing. Cyprinid fishes: systematics, biology and exploitation. 1991:377-412.

26. Hulsey CD, Machado-Schiaffino G, Keicher L, Ellis-Soto D, Henning F and Meyer A. The integrated genomic architecture and evolution of dental divergence in East African cichlid fishes (Haplochromis chilotes x H. nyererei). G3: Genes, Genomes, Genetics. 2017;7 9:3195-202.

27. Jeon SA, Park JL, Park S-J, Kim JH, Goh S-H, Han J-Y, et al. Comparison between MGI and Illumina sequencing platforms for whole genome sequencing. Genes & Genomics. 2021;43:713-24.

28. Chen SF, Zhou YQ, Chen YR and Gu J. fastp: an ultra-fast all-in-one FASTQ preprocessor. Bioinformatics. 2018;34 17:i884-i90. doi:10.1093/bioinformatics/bty560.

29. Hu J, Wang Z, Sun Z, Hu B, Ayoola AO, Liang F, et al. NextDenovo: an efficient error correction and accurate assembly tool for noisy long reads. Genome Biol. 2024; 26;25(1):107. doi: 10.1186/s13059-024-03252-4.

30. Hu J, Fan JP, Sun ZY and Liu SL. NextPolish: a fast and efficient genome polishing tool for long-read assembly. Bioinformatics. 2020;36 7:2253-5. doi:10.1093/bioinformatics/btz891.

31. Cheng HY, Jarvis ED, Fedrigo O, Koepfli KP, Urban L, Gemmell NJ, et al. Haplotype-resolved assembly of diploid genomes without parental data. Nat Biotechnol. 2022;40 9:1332-5. doi:10.1038/s41587-022-01261-x.

32. Rao SSP, Huntley MH, Durand NC, Stamenova EK, Bochkov ID, Robinson JT, et al. A 3D Map of the Human Genome at Kilobase Resolution Reveals Principles of Chromatin Looping. Cell. 2014;159 7:1665-80. doi:10.1016/j.cell.2014.11.021.

33. Li H and Durbin R. Fast and accurate long-read alignment with Burrows-Wheeler transform. Bioinformatics. 2010;26 5:589-95. doi:10.1093/bioinformatics/btp698.

34. Servant N, Varoquaux N, Lajoie BR, Viara E, Chen CJ, Vert JP, et al. HiC-Pro: an optimized and flexible pipeline for Hi-C data processing. Genome Biol. 2015;16:259. doi:10.1186/s13059-015-0831-x.

35. Burton JN, Adey A, Patwardhan RP, Qiu R, Kitzman JO and Shendure J. Chromosome-scale scaffolding of de novo genome assemblies based on chromatin interactions. Nature biotechnology. 2013;31 12:1119-25. doi:10.1038/nbt.2727.

36. Stanke M, Diekhans M, Baertsch R and Haussler D. Using native and syntenically mapped cDNA alignments to improve de novo gene finding. Bioinformatics. 2008;24 5:637-44. doi:10.1093/bioinformatics/btn013.

37. Kim D, Paggi JM, Park C, Bennett C and Salzberg SL. Graph-based genome alignment and genotyping with HISAT2 and HISAT-genotype. Nat Biotechnol. 2019;37 8:907-15. doi:10.1038/s41587-019-0201-4.

38. Eddy SR. A probabilistic model of local sequence alignment that simplifies statistical significance estimation. PLoS Comput Biol. 2008;4 5:e1000069. doi:10.1371/journal.pcbi.1000069.

39. Ou SJ and Jiang N. LTR_FINDER_parallel: parallelization of LTR_FINDER enabling rapid identification of long terminal repeat retrotransposons. Mob DNA. 2019;10:48. doi:10.1186/s13100-019-0193-0.
40. Ellinghaus D, Kurtz S and Willhoeft U. LTRharvest, an efficient and flexible software for de novo detection of LTR retrotransposons. Bmc Bioinformatics. 2008;9:18. doi:10.1186/1471-2105-9-18.
41. Ou SJ and Jiang N. LTR_retriever: A Highly Accurate and Sensitive Program for Identification of Long Terminal Repeat Retrotransposons. Plant Physiol. 2018;176 2:1410-22. doi:10.1104/pp.17.01310.
42. Chan PP, Lin BY, Mak AJ and Lowe TM. tRNAscan-SE 2.0: improved detection and functional classification of transfer RNA genes. Nucleic Acids Res. 2021;49 16:9077-96. doi:10.1093/nar/gkab688.
43. Armstrong J, Hickey G and Diekhans M. Progressive Cactus is a multiple-genome aligner for the thousand-genome era. Nature. 2020;587 7833:246-51. doi:10.1038/s41586-020-2871-y.
44. Höhler D, Pfeiffer W, Ioannidis V, Stockinger H and Stamatakis A. RAxML Grove: an empirical phylogenetic tree database. Bioinformatics. 2022;38 6:1741-2. doi:10.1093/bioinformatics/btab863.
45. Zhang C, Scornavacca C, Molloy EK and Mirarab S. ASTRAL-Pro: Quartet-Based Species-Tree Inference despite Paralogy. Mol Biol Evol. 2020;37 11:3292-307. doi:10.1093/molbev/msaa139.
46. Yang ZH. PAML 4: phylogenetic analysis by maximum likelihood. Mol Biol Evol. 2007;24 8:1586-91. doi:10.1093/molbev/msm088.
47. Cooper GM, Stone EA, Asimenos G, Green ED, Batzoglou S and Sidow A. Distribution and intensity of constraint in mammalian genomic sequence. Genome research. 2005;15 7:901-13. doi:10.1101/gr.3577405.
48. Chen L and Qiu Q. Large-scale ruminant genome sequencing provides insights into their evolution and distinct traits. 2019;364 6446 doi:10.1126/science.aav6202.
49. Malinsky M, Matschiner M and Svardal H. Dsuite - Fast D-statistics and related admixture evidence from VCF files. Mol Ecol Resour. 2021;21 2:584-95. doi:10.1111/1755-0998.13265.
50. Rio DC, Ares M, Hannon GJ and Nilsen TW. Purification of RNA using TRIzol (TRI reagent). Cold Spring Harb Protoc. 2010;2010 6:pdb.prot5439. doi:10.1101/pdb.prot5439.
51. Patterson J, Carpenter EJ, Zhu Z, An D, Liang X, Geng C, et al. Impact of sequencing depth and technology on de novo RNA-Seq assembly. BMC genomics. 2019;20 1:604. doi:10.1186/s12864-019-5965-x.
52. Chen YX, Chen YS, Shi CM, Huang ZB, Zhang Y, Li SK, et al. SOAPnuke: a MapReduce acceleration-supported software for integrated quality control and preprocessing of high-throughput sequencing data. GigaScience. 2017;7 1:1-6. doi:10.1093/gigascience/gix120.
53. Danecek P, Bonfield JK, Liddle J, Marshall J, Ohan V, Pollard MO, et al. Twelve years of SAMtools and BCFtools. Gigascience. 2021,10(2):giab008. doi: 10.1093/gigascience/giab008.
54. Srinivasan KA, Virdee SK and McArthur AG. Strandedness during cDNA synthesis, the stranded parameter in htseq-count and analysis of RNA-Seq data. Brief Funct Genomics. 2020;19 5-6:339-42. doi:10.1093/bfgp/elaa010.
55. Varet H, Brillet-Gueguen L, Coppee JY and Dillies MA. SARTools: A DESeq2- and EdgeR-Based R Pipeline for Comprehensive Differential Analysis of RNA-Seq Data. PLoS One. 2016;11 6:e0157022. doi:10.1371/journal.pone.0157022.
56. Zhang Z. KaKs_Calculator 3.0: Calculating Selective Pressure on Coding and Non-coding Sequences. Genomics Proteomics Bioinformatics. 2022; doi:10.1016/j.gpb.2021.12.002.
57. Liu H, Chen C, Lv M, Liu N, Hu Y, Zhang H, et al. A Chromosome-Level Assembly of Blunt Snout Bream (Megalobrama amblycephala) Genome Reveals an Expansion of Olfactory Receptor Genes in Freshwater Fish. Molecular Biology and Evolution. 2021;38 10:4238-51. doi:10.1093/molbev/msab152.
58. Jiang H, Qian Y, Zhang Z, Meng M, Deng Y, Wang G, et al. Chromosome-level genome assembly and whole-genome resequencing of topmouth culter (Culter alburnus) provide insights into the intraspecific variation of its semi-buoyant and adhesive eggs. Molecular ecology resources. 2023;23 8:1841-52. doi:10.1111/1755-

0998.13845.
59. Ye M. DESCRIPTION OF A NEW GENUS AND SPECIES OF DANIONINAE FROM CHINA (CYPRINIFORMES: CYPRINIDAE). 1983.
60. Chen F, Xue G, Wang YK, Zhang HC, Clift PD, Xing YW, et al. Evolution of the Yangtze River and its biodiversity. Innovation (Cambridge (Mass)). 2023;4 3:100417. doi:10.1016/j.xinn.2023.100417.
61. Feng CG, Wang K, Xu WJ, Yang LD, Wanghe KY, Sun N, et al. Monsoon boosted radiation of the endemic East Asian carps. Sci China Life Sci. 2023;66 3:563-78. doi:10.1007/s11427-022-2141-1.
62. Feng SH, Bai M, Rivas-Gonzalez I, Li C, Liu SP, Tong YJ, et al. Incomplete lineage sorting and phenotypic evolution in marsupials. Cell. 2022;185 10:1646-60.e18. doi:10.1016/j.cell.2022.03.034.
63. Martin SH, Davey JW and Jiggins CD. Evaluating the use of ABBA-BABA statistics to locate introgressed loci. Mol Biol Evol. 2015;32 1:244-57. doi:10.1093/molbev/msu269.
64. Ren L, Li WH, Qin QB, Dai H, Han FM, Xiao J, et al. The subgenomes show asymmetric expression of alleles in hybrid lineages of Megalobrama amblycephala x Culter alburnus. Genome Res. 2019;29 11:1805-15. doi:10.1101/gr.249805.119.
65. Tautz D and Domazet-Lošo T. The evolutionary origin of orphan genes. Nat Rev Genet. 2011;12 10:692-702. doi:10.1038/nrg3053.
66. Hayden B, Palomares MLD, Smith BE and Poelen JH. Biological and environmental drivers of trophic ecology in marine fishes - a global perspective. Sci Rep. 2019;9 1:11415. doi:10.1038/s41598-019-47618-2.
67. Zhou X, Khan S, Huang DB and Li L. V-Set and immunoglobulin domain containing (VSIG) proteins as emerging immune checkpoint targets for cancer immunotherapy. Front Immunol. 2022;13:938470. doi:10.3389/fimmu.2022.938470.
68. Miao EA, Rajan JV and Aderem A. Caspase-1-induced pyroptotic cell death. Immunological reviews. 2011;243 1:206-14.
69. Felthaus O, Gosau M and Morsczeck C. ZBTB16 induces osteogenic differentiation marker genes in dental follicle cells independent from RUNX2. Journal of periodontology. 2014;85 5:e144-51. doi:10.1902/jop.2013.130445.
70. Sribudiani Y, Chauhan RK, Alves MM, Petrova L, Brosens E, Harrison C, et al. Identification of Variants in RET and IHH Pathway Members in a Large Family With History of Hirschsprung Disease. Gastroenterology. 2018;155 1:118-29.e6. doi:10.1053/j.gastro.2018.03.034.
71. Rai K, Sarkar S, Broadbent TJ, Voas M, Grossmann KF, Nadauld LD, et al. DNA demethylase activity maintains intestinal cells in an undifferentiated state following loss of APC. Cell. 2010;142 6:930-42. doi:10.1016/j.cell.2010.08.030.
72. Zoodsma JD, Keegan EJ, Moody GR and Bhandiwad AA. Disruption of grin2B, an ASD-associated gene, produces social deficits in zebrafish. 2022;13 1:38. doi:10.1186/s13229-022-00516-3.
73. Zoodsma JD, Chan K, Bhandiwad AA, Golann DR, Liu G, Syed SA, et al. A Model to Study NMDA Receptors in Early Nervous System Development. The Journal of neuroscience: the official journal of the Society for Neuroscience. 2020;40 18:3631-45. doi:10.1523/jneurosci.3025-19.2020.
74. Li DY, Jiang XD, Gong W and Li CY. Tectonic uplift along the northeastern margin of the Qinghai–Tibetan Plateau: Constraints from the lithofacies sequence and deposition rate of the Qaidam Basin. Tectonophysics. 2022;827:229279. doi:10.1016/j.tecto.2022.229279.
75. Hren MT, Sheldon ND, Grimes ST, Collinson ME, Hooker JJ, Bugler M, et al. Terrestrial cooling in Northern Europe during the eocene-oligocene transition. Proceedings of the National Academy of Sciences of the United States of America. 2013;110 19:7562-7. doi:10.1073/pnas.1210930110.
76. Xing YC, Zhang CG, Fan E and Zhao YH. Freshwater fishes of China: species richness, endemism, threatened species and conservation. Divers Distrib. 2016;22.

77. Murat F, Mbengue N, Winge SB, Trefzer T, Leushkin E, Sepp M, et al. The molecular evolution of spermatogenesis across mammals. Nature. 2023;613 7943:308-16. doi:10.1038/s41586-022-05547-7.
78. Brawand D, Soumillon M, Necsulea A, Julien P, Csardi G, Harrigan P, et al. The evolution of gene expression levels in mammalian organs. Nature. 2011;478 7369:343-8. doi:10.1038/nature10532.
79. Carruthers M, Edgley DE, Saxon AD, Gabagambi NP, Shechonge A, Miska EA, et al. Ecological Speciation Promoted by Divergent Regulation of Functional Genes Within African Cichlid Fishes. Mol Biol Evol. 2022;39 11:msac251. doi:10.1093/molbev/msac251.
80. Olave M, Nater A, Kautt AF and Meyer A. Early stages of sympatric homoploid hybrid speciation in crater lake cichlid fishes. Nature communications. 2022;13 1:5893. doi:10.1038/s41467-022-33319-4.
81. He S, Li L, Lv LY, Cai WJ, Dou YQ, Li J, et al. Mandarin fish (Sinipercidae) genomes provide insights into innate predatory feeding. Commun Biol. 2020;3 1:361. doi:10.1038/s42003-020-1094-y.
82. Jawad LA, Agha GF, Abdullah SMA, Aguilar G and Qasim AM. Morphology and morphometry of pharyngeal bone and teeth in cyprinid species from the Kurdistan Region, Iraq. Anatomical record. 2022;305 11:3356-66. doi:10.1002/ar.24906.
83. Gu QH, Yuan H, Zhong H, Wei ZH, Shu YQ, Wang J, et al. Spatiotemporal characteristics of the pharyngeal teeth in interspecific distant hybrids of cyprinid fish: Phylogeny and expression of the initiation marker genes. Front Genet. 2022;13:983444. doi:10.3389/fgene.2022.983444.
84. Hartenstein V and Martinez P. Structure, development and evolution of the digestive system. Cell Tissue Res. 2019;377 3:289-92. doi:10.1007/s00441-019-03102-x.
85. Xiao J, Kang XW, Xie LH, Qin QB, He ZL, Hu FZ, et al. The fertility of the hybrid lineage derived from female Megalobrama amblycephala x male Culter alburnus. Anim Reprod Sci. 2014;151 1-2:61-70. doi:10.1016/j.anireprosci.2014.09.012.
86. Owens GL and Samuk K. Adaptive introgression during environmental change can weaken reproductive isolation. Nat Clim Change. 2019;10 1:58-62. doi:10.1038/s41558-019-0628-0.
87. Chen J, Liu H, Gooneratne R, Wang Y and Wang WM. Population Genomics of Megalobrama Provides Insights into Evolutionary History and Dietary Adaptation. Biology (Basel). 2022;11 2:186. doi:10.3390/biology11020186.
88. Ren L. The assembled genomes and annotation files of eight cyprinid fishes. figshare. Dataset. 2023. https://doi.org/10.6084/m9.figshare.24125487.v1
89. Ren L, Tu X, Luo M, Liu Q, Cui J, Gao X, et al. Supporting data for "Genomes reveal pervasive distant hybridization in nature among cyprinid fishes". GigaScience Database. 2024; https://doi.org/10.5524/102603
90. Ren L, Tu X, Luo M, Liu Q, Cui J, Gao X, et al. Genome data of the cyprinid fish, Culter alburans (Topmouth Culter). GigaScience Database. 2024; https://doi.org/10.5524/102610
91. Ren L, Tu X, Luo M, Liu Q, Cui J, Gao X, et al. Genome data of the cyprinid fish, Megalobrama amblycephala (blunt snout bream). GigaScience Database. 2024; https://doi.org/10.5524/102611
92. Ren L, Tu X, Luo M, Liu Q, Cui J, Gao X, et al. Genome data of the cyprinid fish, Gobiocypris rarus (rare gudgeon). GigaScience Database. 2024; https://doi.org/10.5524/102612
93. Ren L, Tu X, Luo M, Liu Q, Cui J, Gao X, et al. Genome data of the cyprinid fish, Cirrhinus molitorella (mud carp). GigaScience Database. 2024; https://doi.org/10.5524/102613
94. Ren L, Tu X, Luo M, Liu Q, Cui J, Gao X, et al. Genome data of the cyprinid fish, Pseudorasbora parva (stone moroko). GigaScience Database. 2024; https://doi.org/10.5524/102614
95. Ren L, Tu X, Luo M, Liu Q, Cui J, Gao X, et al. Genome data of the cyprinid fish, Xenocypris davidi (Bleekers yellow tail). GigaScience Database. 2024; https://doi.org/10.5524/102615
96. Ren L, Tu X, Luo M, Liu Q, Cui J, Gao X, et al. Genome data of the cyprinid fish, Elopichthys bambusa (yellow cheek carp). GigaScience Database. 2024; https://doi.org/10.5524/102616
97. Ren L, Tu X, Luo M, Liu Q, Cui J, Gao X, et al. Genome data of the cyprinid fish, Ctenopharyngodon idella (grass

carp). GigaScience Database. 2024; https://doi.org/10.5524/102617

Figures legend

Figure 1: Phylogenomic analyses of cyprinid fish

(a) Time-calibrated phylogenetic tree. Red pot represents fossil calibration time points, which were obtained from “Timetree of Life”. Black number in the branch represents the median of the range of divergence times. The name marked in red represents the species sequenced in this study. (b) Gene flow determined using the f-branch method. Dashed red line represents gene flow (f4-ratio > 0.05) between two species.

Figure 2: Divergent evolution of *M. amblycephala* and *C. alburnus* in East Asia

(a) Habitat distribution of extant *M. amblycephala* [87] and *C. alburnus*. Partial overlap in the habitats of the two species. (b) The number and percentage of OSGs in *M. amblycephala* and *C. alburnus*. (c) The number and percentage of OGs in *M. amblycephala* and *C. alburnus*. (d) Differential expression between *M. amblycephala* and *C. alburnus* in six tissues and organs. (e) The distribution of Ka/Ks values relating to OSGs in the six tissues and organs. The median value (black dot) was signed in figure. (f) Conserved scores of OSGs in the six tissues and organs. The median value (black line and number) was signed in figure. The comparisons involve the intestine and testis. “*” represents “$0.01 < p$-value ≤ 0.05”, “**” represents “$0.001 < p$-value ≤ 0.01”, and “***” represents “p-value ≤ 0.001”.

Figure 3: Diet divergences between *M. amblycephala* and *C. alburnus*

(a) The four genes relating to differential expression between *M. amblycephala* (BSB) and *C. alburnus* (TC) in both the intestine and liver (three biological replicates showed “_1”, “_2”, and “_3”). (b) Significant differences in the enzyme activity of lipase and trypsin for the comparison between *M. amblycephala* and *C. alburnus.*

Figure 4: Gene flow in herbivorous and carnivorous fishes

(a) Habitat distribution of *M. amblycephala* [87], *C. alburnus*, *C. idella*, and *E. bambusa* in East Asia. Their overlap and unique habitats reflect their speciation of endemic East Asian cyprinid fishes in the river-lake ecosystems of East Asia. (b) Venn diagram showing introgressed genes between carnivores and herbivores. (c) The introgressed genes were expressed in different tissues and organs, while there was no introgressed gene expressed in the brain. (d) GO analyses of the shared 10 introgressed genes in carnivores and herbivores. (e) Phylogenomic analyses of the six endemic cyprinid species in China, introgressions visualizing at SNPs of *zbtb16a* (potential introgressed SNPs marking red arrow), the morphologies of mouth, pharyngeal teeth, and intestine, microstructure of intestine (thickness of intestinal wall marking in figure) and muscle (average area marking in figure) in the four endemic cyprinid species. Blue dot represents filter-feeding fish, green dot represents herbivorous fish, and red dot represents carnivorous fish.

Table

Table 1. Assembly statistics of eight species of cyprinid fishes

Species	Common name	Subfamily	Scaffold N50 (Mb)	Contig N50 (Mb)	Contig length (Mb)	BUSCO Completeness (%)
Megalobrama amblycephala	Blunt snout bream	*Cultrinae*	42.91	15.42	1096.68	94.5%
Culter alburnus	Topmouth culter	*Cultrinae*	39.60	18.55	1077.98	98.3%
Ctenopharyngodon idella	Grass carp	*Leuciscinae*	/	35.62	901.49	98.5%
Cirrhinus molitorella	Mud carp	*Labeoninae*	/	38.12	1066.79	98.6%
Pseudorasbora parva	Stone moroko	*Gobioninae*	/	7.57	1292.12	98.5%
Xenocypris davidi	Bleeker's yellow tail	*Xenocyprinae*	/	38.11	1044.52	98.7%
Gobiocypris rarus	Raregudgeon	*Danioninae*	/	13.24	1108.08	98.3%
Elopichthys bambusa	Yellow cheek carp	*Leuciscinae*	/	30.38	863.54	98.4%

Figure 1

Click here to access/download;Figure;fig1.tif

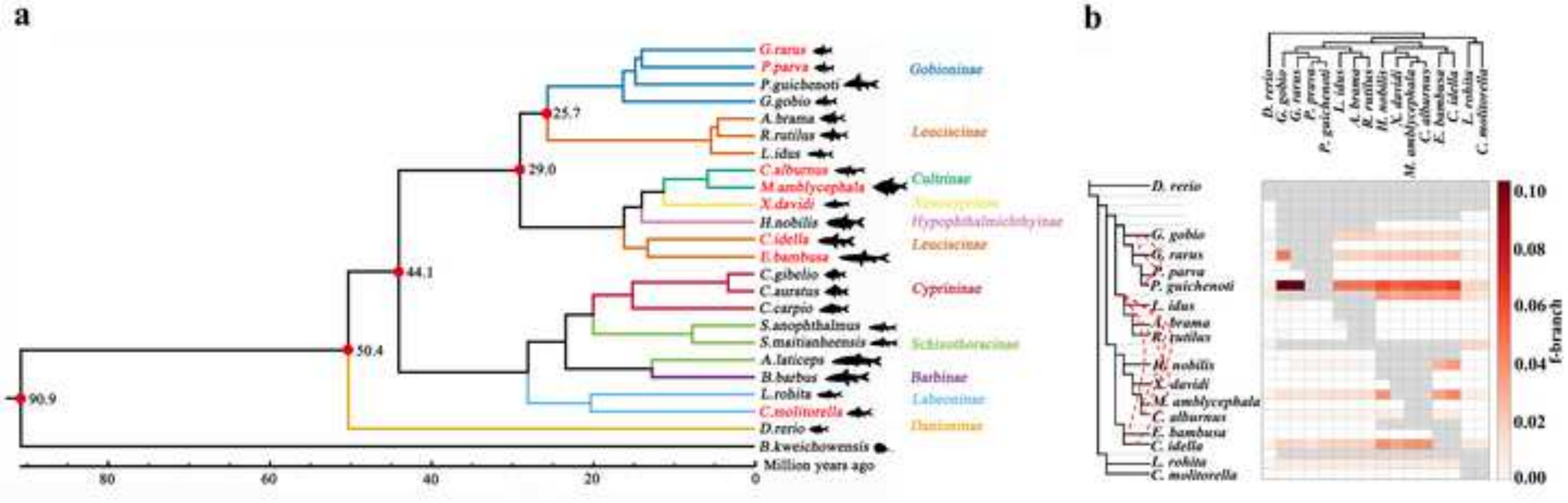

Figure 2

Click here to access/download;Figure;fig2.tif

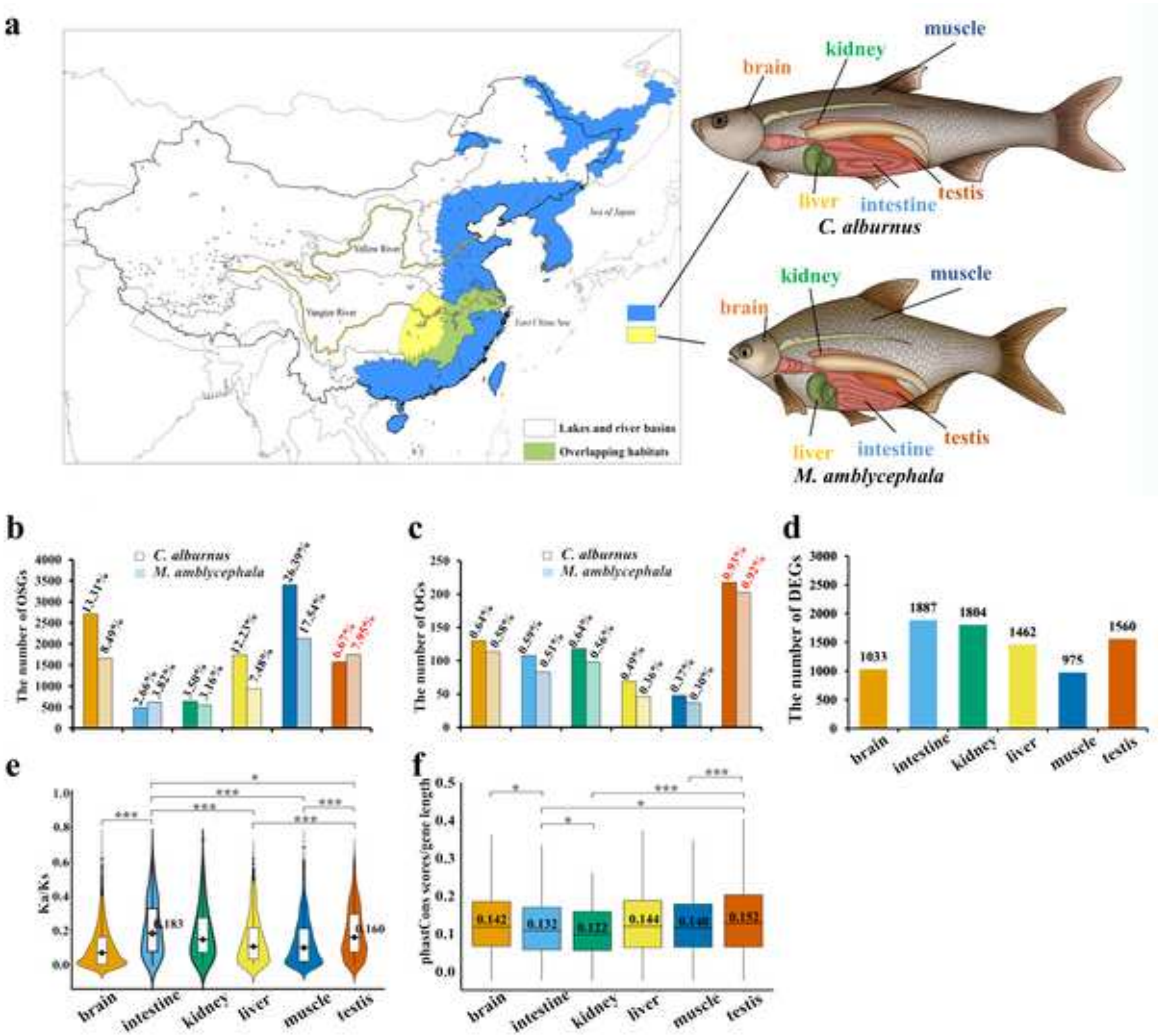

Figure 3

Click here to access/download;Figure;fig3.tif

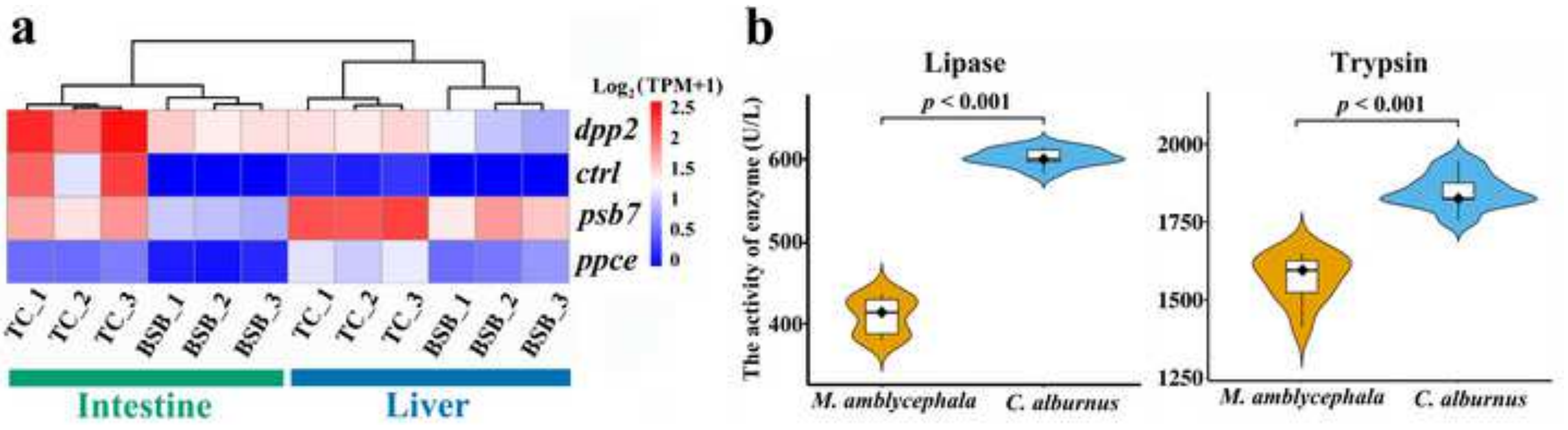

Figure 4

Click here to access/download;Figure;fig4.tif

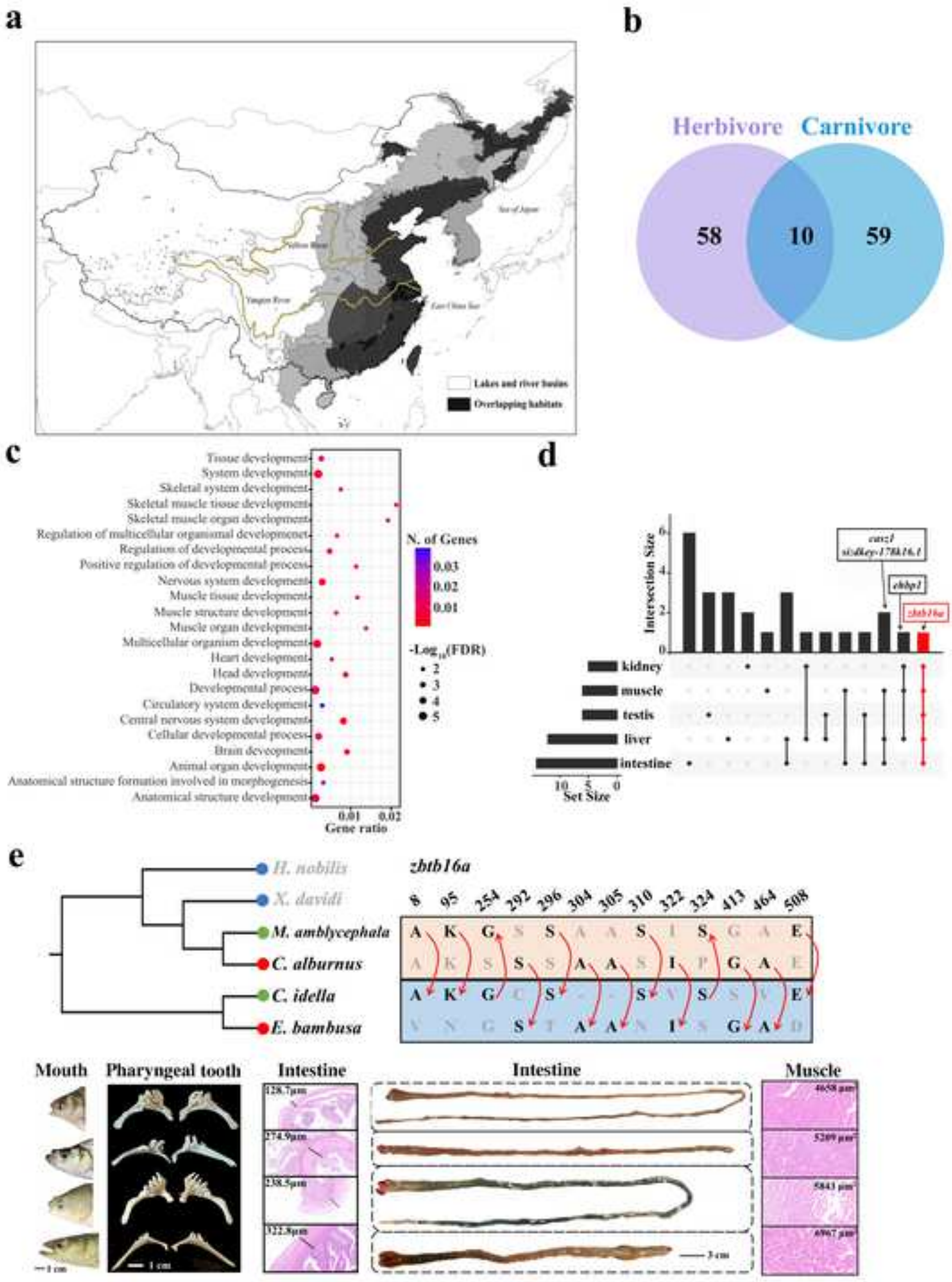

Supplemental File 1

Click here to access/download
Supplementary Material
Supplementary Tables.xlsx

Supplemental File 2

Click here to access/download
Supplementary Material
Supplementary Figures.pdf
