# Supplementary material for: Genomes reveal pervasive distant hybridization in nature among cyprinid fishes
Source: Gigascience. 2025 Jan 30;14:giae117. doi: 10.1093/gigascience/giae117 (PMC11779505; doi:10.1093/gigascience/giae117)
Supplement: giae117_Supplemental_Figures_and_Tables [file giae117_supplemental_figures_and_tables.zip › Supplementary Figures.pdf]

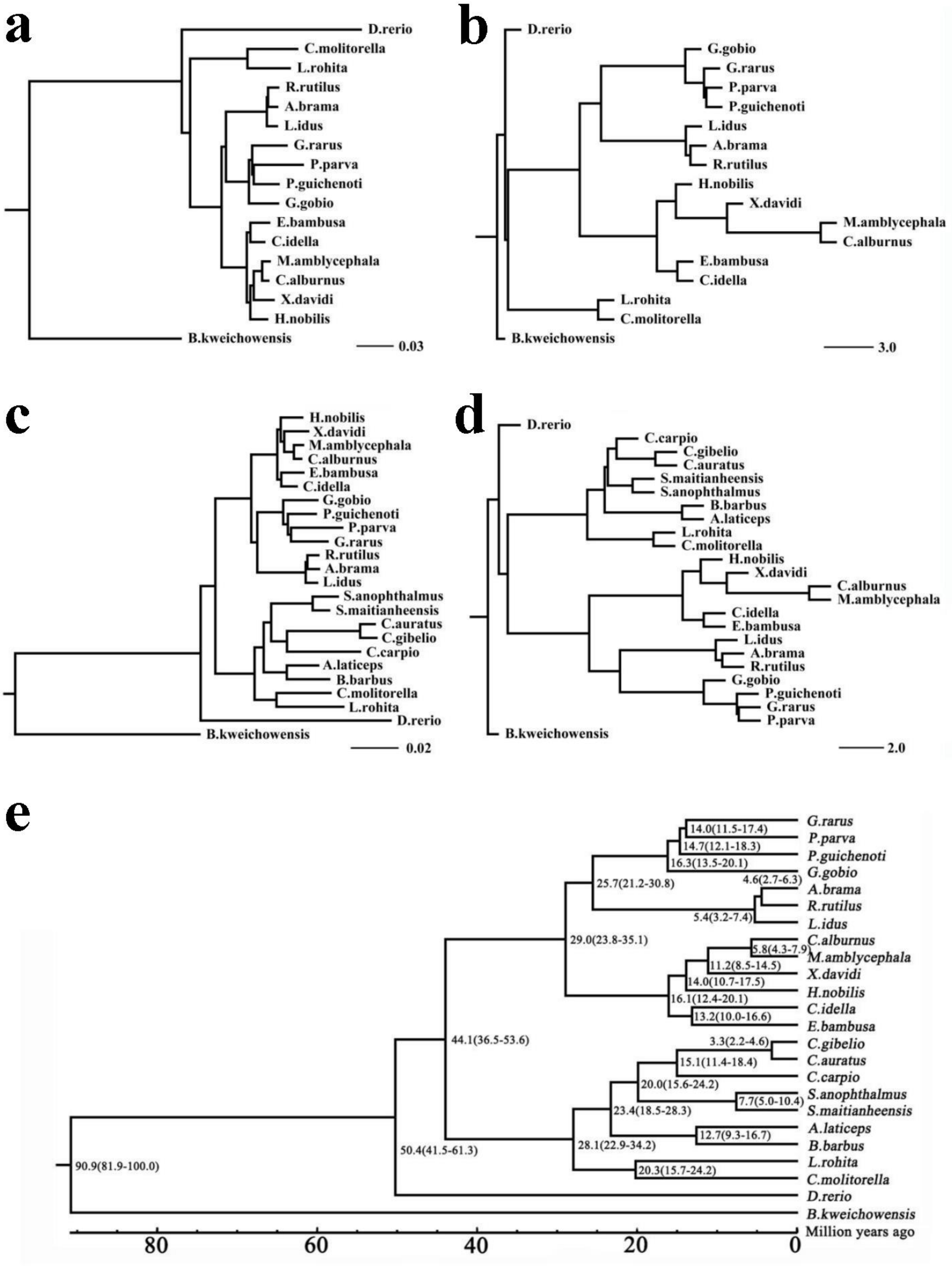

Figure S1. Phylogenetic trees constructed using multiple whole-genome alignments of 17 (no polyploid species) and 24 (including 7 polyploid species) species with *Beaufortia kweichowensis* as the root, respectively.

a. Concatenation-based method for estimating a phylogenetic tree of 17 species with 10-kb length windows.

Bootstrap support values = 100% in each tree.

b. Coalescent method for estimating a phylogenetic tree of 17 species with 10-kb length windows. Bootstrap support values = 100% in each tree.

c. Concatenation-based method for estimating a phylogenetic tree of 24 species with 10-kb length windows. Bootstrap support values = 100% in each tree.

d. Coalescent method for estimating a phylogenetic tree of 24 species with 10-kb length windows. Bootstrap support values = 100% in each tree.

d. Species tree with estimated divergence time.

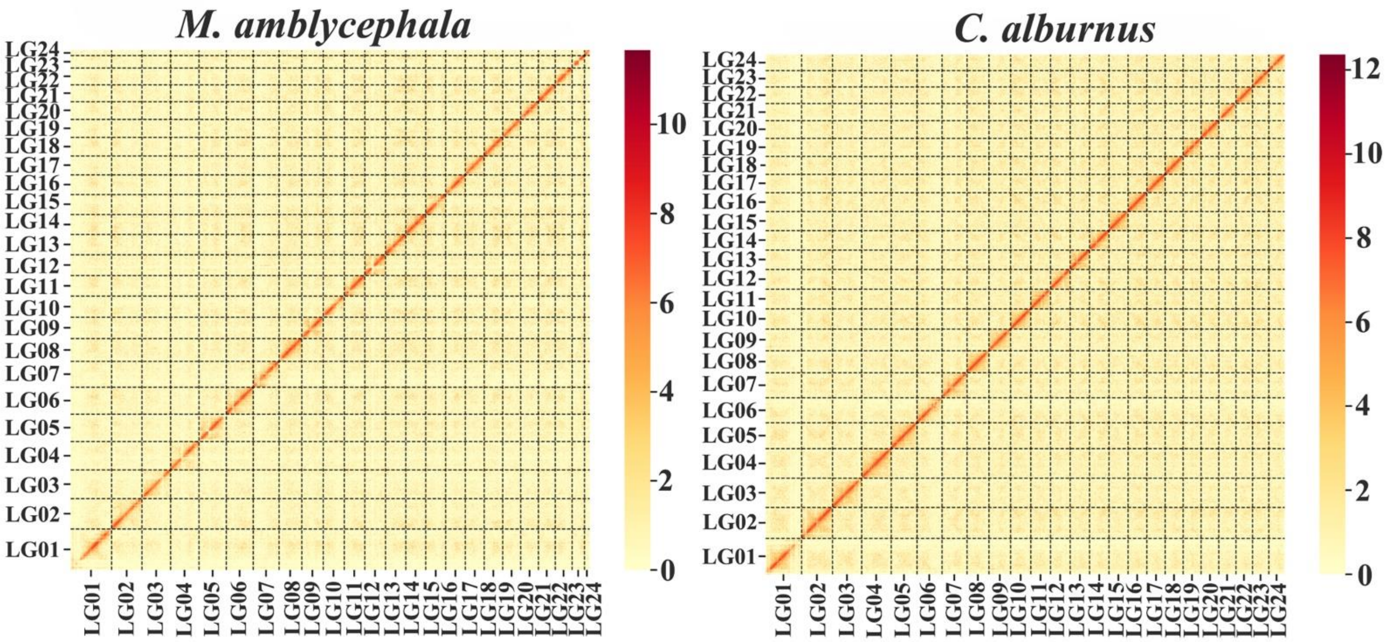

Figure S2. The Hi-C interaction heatmap of 24 linkage groups in the genomes of *Megalobrama amblycephala* and *Culter alburnus*.

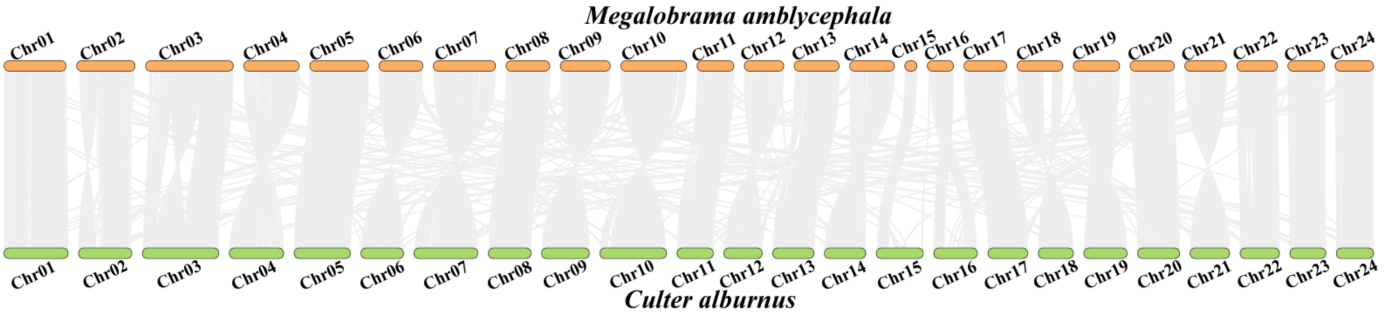

Figure S3. The collinearity analysis between *M. amblycephala* and *C. alburnus*. Twenty-four pairs of homologous chromosomes were determined based on 17,337 orthologous gene pairs.

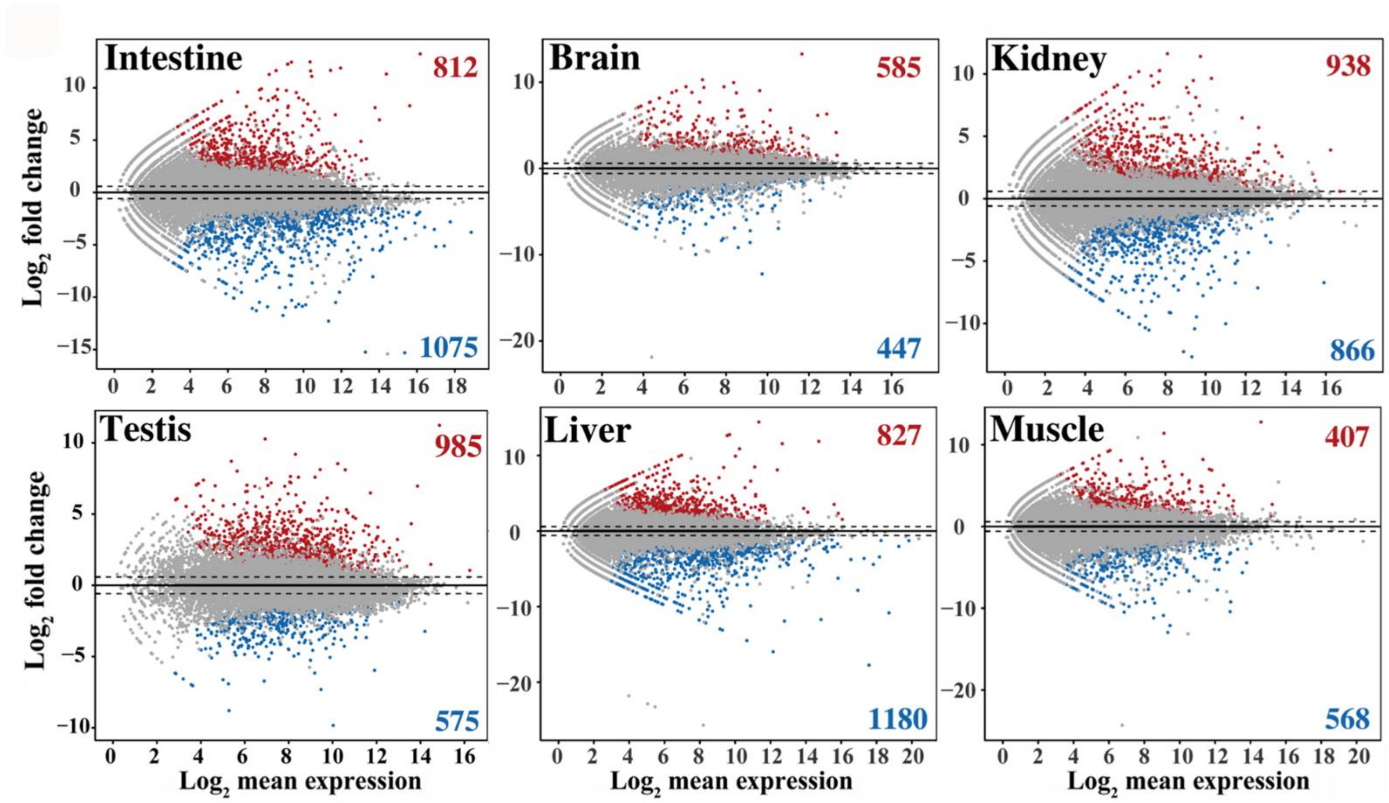

Figure S4. The differential expression between *M. amblycephala* and *C. alburn*us in six organs. Up-regulated genes in *M. amblycephala* are marked in blue, while the up-regulated genes in *C. alburnus* are marked in red.

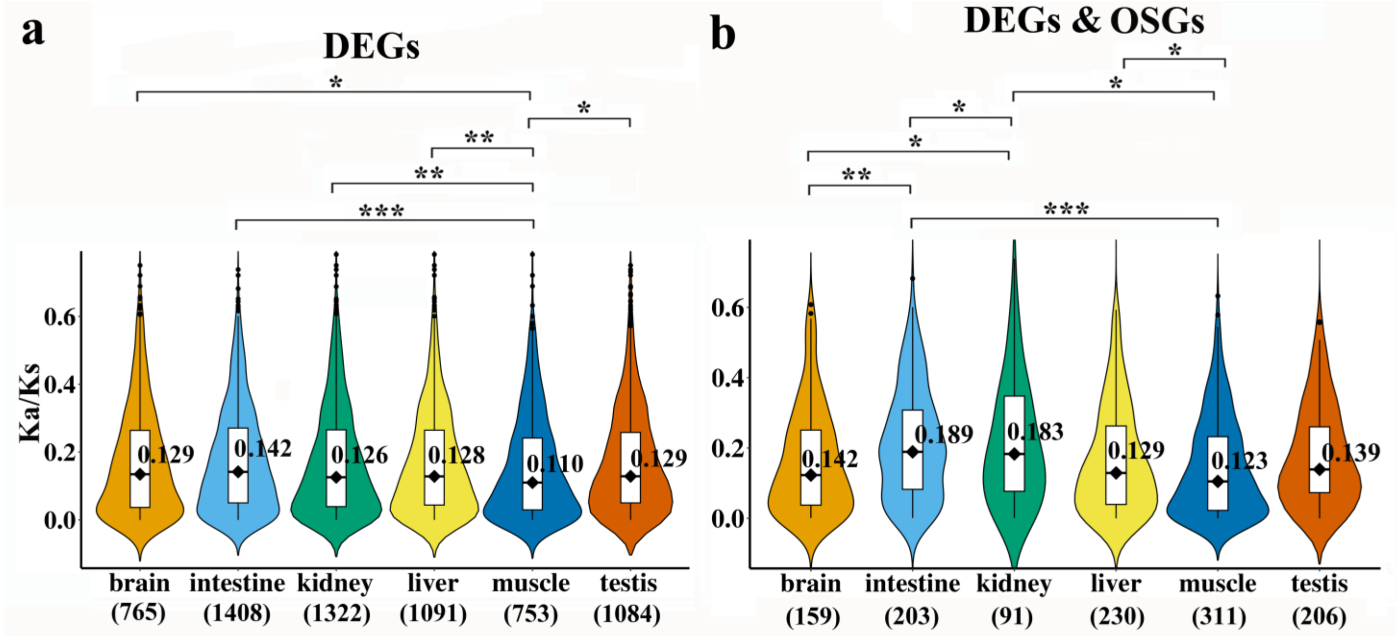

Figure S5. The distribution of Ka/Ks values in the six organs.

a. The Ka/Ks values of DEGs.

b. The Ka/Ks values of share genes between DEGs and OSGs. The median value is indicated by a black dot, and the gene number is provided below each organ name. In the *t*-test, “*” represents $0.01 < p\text{-value} \leq 0.05$,

"**" represents 0.001 < *p*-value ≤ 0.01, "***" represents *p*-value ≤ 0.001.

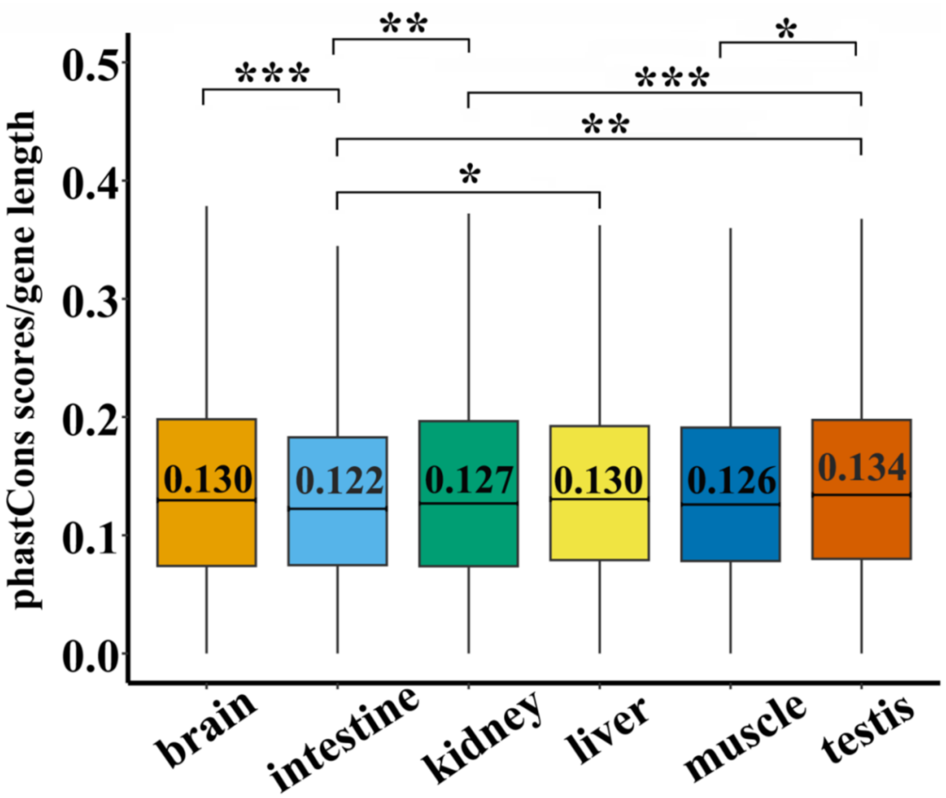

Figure S6. Conserved scores of DEGs (*M. amblycephala vs. C. alburnus*) in the six organs. "*" represents 0.01 < *p*-value ≤ 0.05, "**" represents 0.001 < *p*-value ≤ 0.01, "***" represents *p*-value ≤ 0.001.

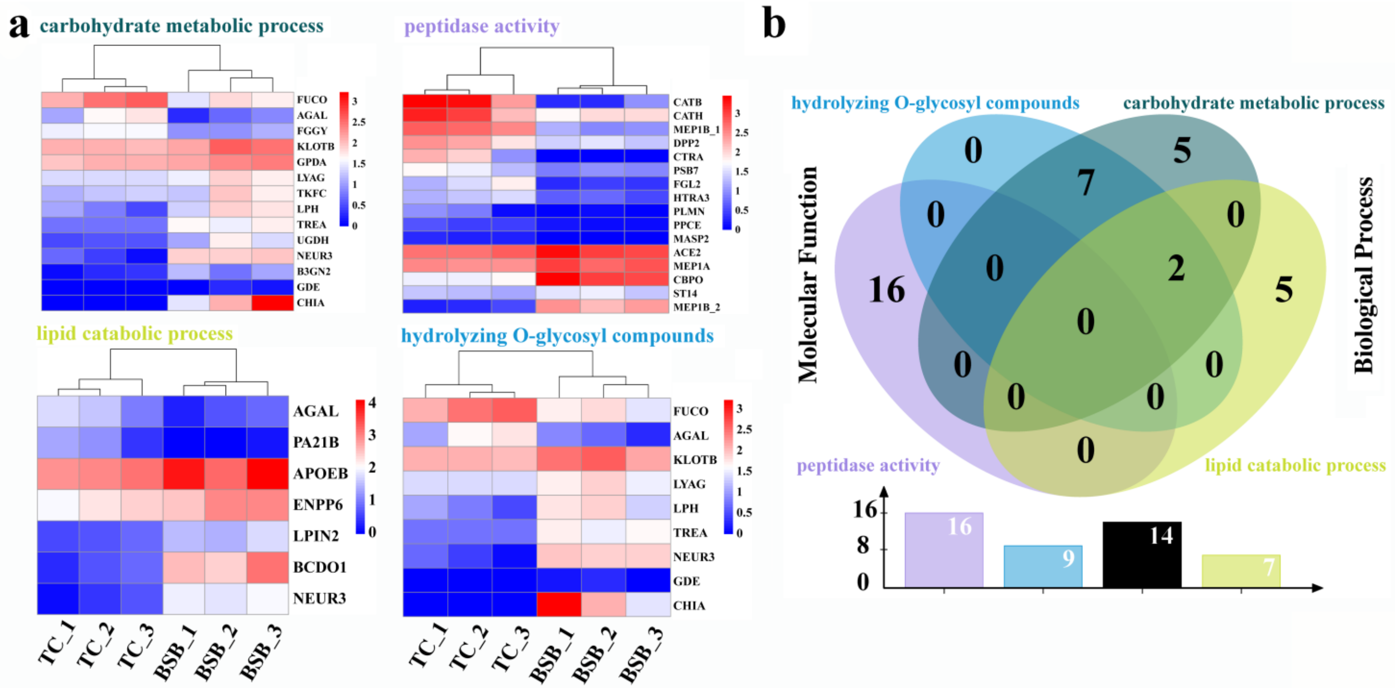

Figure S7. The DEGs (*M. amblycephala vs. C. alburnus*) associated with diet habit.

a. The heatmap of the DEGs in the intestine. The hydrolyzing O-glycosyl compounds and peptidase activity

in Molecular Function, as well as carbohydrate metabolic process and lipid catabolic process in Biological Process. TC: *C. alburnus*, BSB: *M. amblycephala.*

b. The gene distribution of DEGs in the intestine.

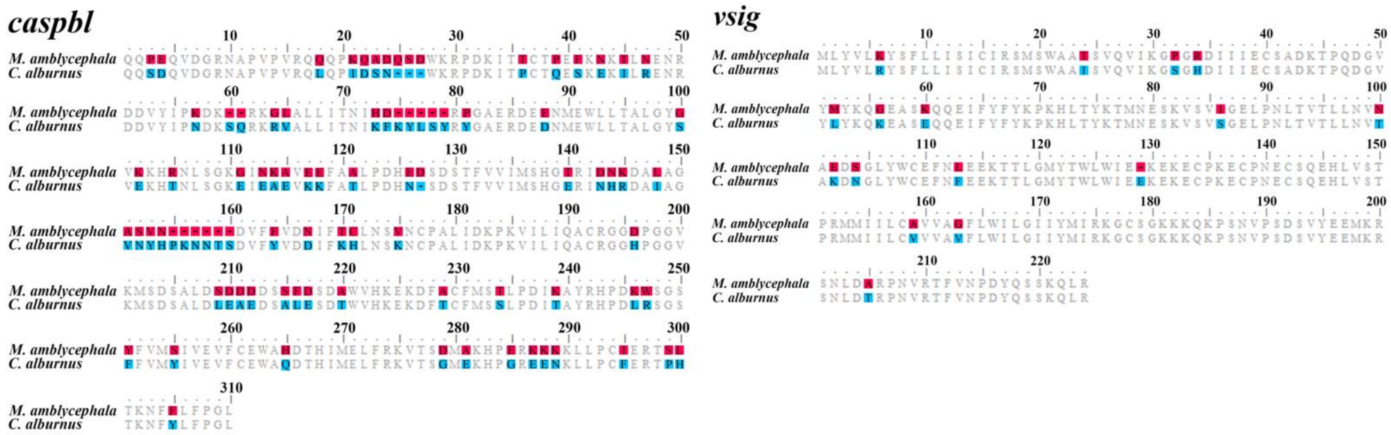

Figure S8. The alignment of two positively selected genes (PSGs) in intestine.

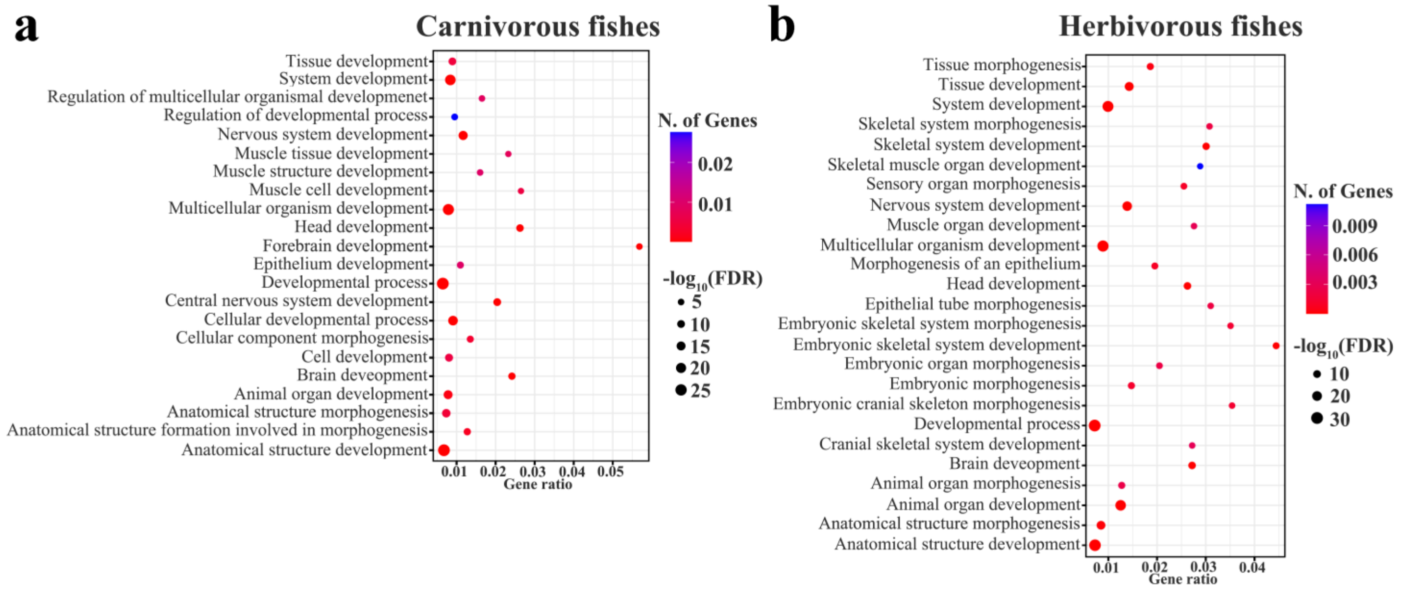

Figure S9. The GO terms of introgressed genes in the carnivorous (*C. alburnus* and *E. bambusa*) and herbivorous (*M. amblycephala* and *C. idella*) fishes.

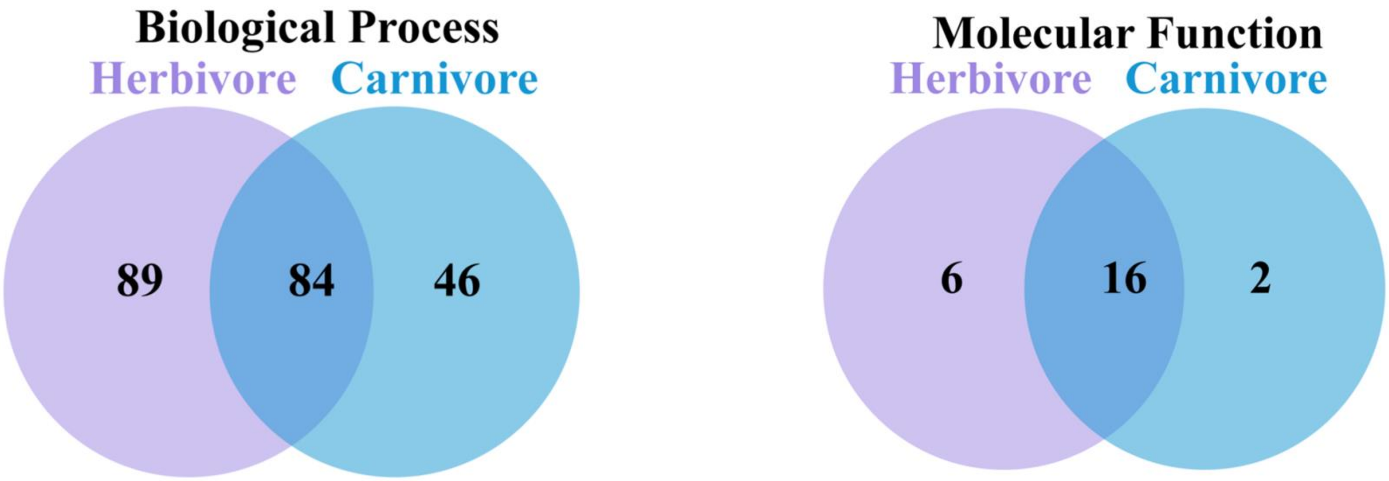

Figure S10. The distribution of enriched functional categories (FDR < 0.05) in Biological Process and Molecular Function for the introgressed genes. Carnivore: *C. alburnus* and *Elopichthys bambusa*, herbivore: *M. amblycephala* and *Ctenopharyngodon idella*.

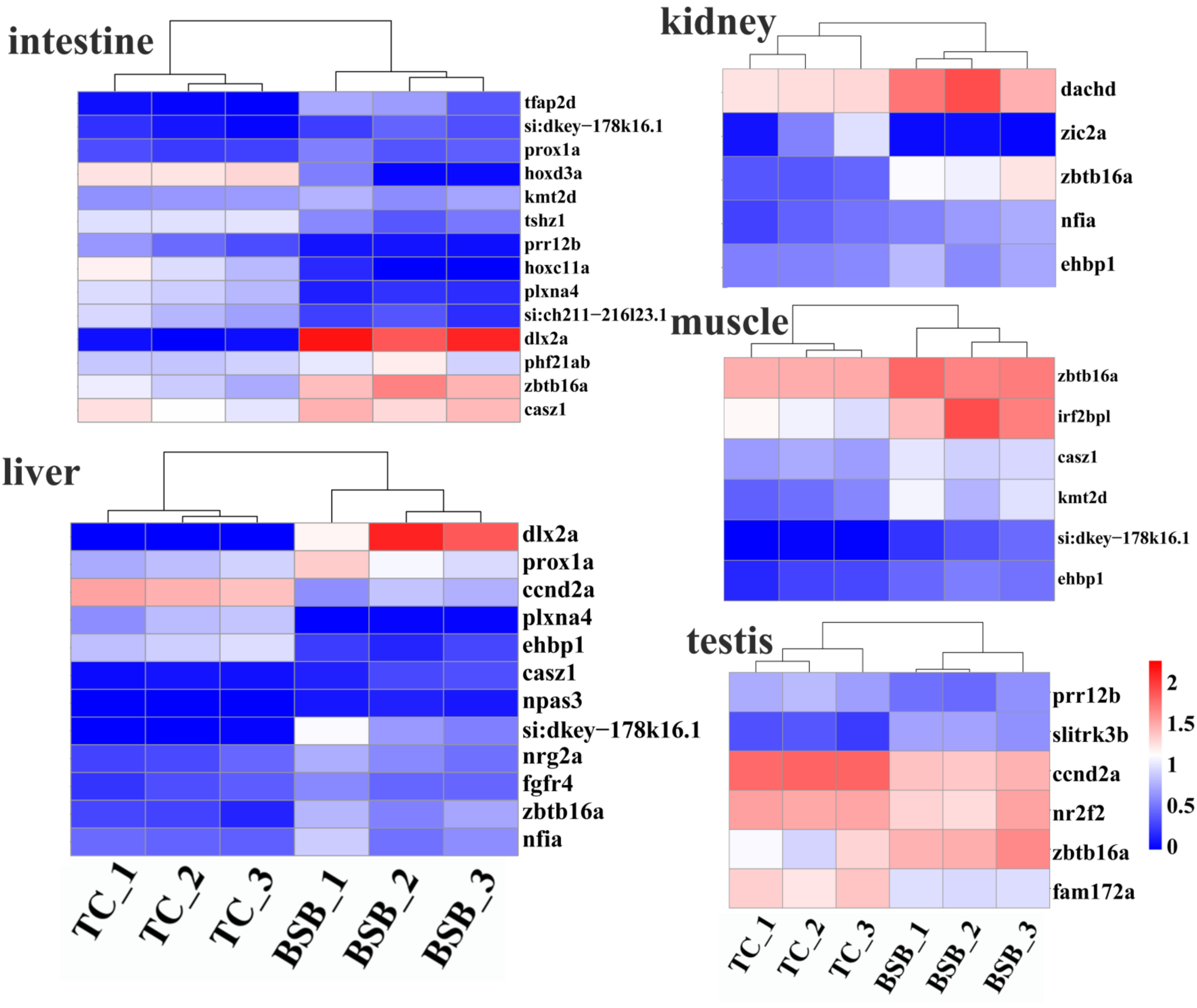

Figure S11. Heatmap exhibiting the expression of introgressed genes in the carnivorous (*C. alburnus*

and *E. bambusa*) and herbivorous (*M. amblycephala* and *C. idella*) fishes. TC: *C. alburnus*, BSB: *M. amblycephala*.
