# Supplementary material for: Genomes reveal pervasive distant hybridization in nature among cyprinid fishes
Source: Gigascience. 2025 Jan 30;14:giae117. doi: 10.1093/gigascience/giae117 (PMC11779505; doi:10.1093/gigascience/giae117)
Supplement: giae117_Response_to_Reviewer_Comments_Original_Submission [file giae117_response_to_reviewer_comments_original_submission.pdf]

Author's Response To Reviewer Comments

Dear Dr. Hongfang Zhang,
Please find the revised manuscript entitled “Genomes reveal pervasive distant hybridization in nature among cyprinid fishes” (GIGA-D-24-00199R1), which we would like to submit for publication as original research in GigaScience.
The comments provided by the editors and reviewers have enabled us to further improve the quality of our manuscript. In the revised version of the manuscript, we have made comprehensive revisions to enhance the conciseness and biological perspective of the entire text. We have highlighted the revised sections in yellow. Detailed explanations and our point-by-point responses to each of the comments are provided in the following pages.

We are looking forward to hearing from you at your earliest convenience.

Sincerely!

Corresponding author: Shaojun Liu
State Key Laboratory of Developmental Biology of Freshwater Fish, Hunan Normal University, Changsha, 410081, P.R. of China

Corresponding author: Dongdong Wu
State Key Laboratory of Genetic Resources and Evolution, Kunming Institute of Zoology, Chinese Academy of Sciences, Kunming, 650201, China

Responses to the comments of the editors:
Please include a point-by-point within the 'Response to Reviewers' box in the submission system. Please ensure you describe additional experiments that were carried out and include a detailed rebuttal of any criticisms or requested revisions that you disagreed with. Please also ensure that your revised manuscript conforms to the journal style, which can be found in the Instructions for Authors on the journal homepage. If the data and code has been modified in the revision process please be sure to update the public versions of this too.
Answer
We appreciate your positive feedback. We have addressed all of the reviewers’ comments and made the necessary revisions to the manuscript. Furthermore, we have ensured that the manuscript adheres to the journal’s formatting requirements. Please note that we have not updated the code.

Responses to the comments of the reviewer 1:
This paper reported their work of genome sequencing and assembly of eight cyprinid fishes and phylogenetic analyses using genomic sequences from a couple of dozens of species. They found widespread allele sharing across species boundaries within seven subfamilies of cyprinid fishes. The major findings are incomplete reproductive isolation and frequent gene flow among some of these species. In spite of being expected, this research provided empirical evidence, which is significant. As such, it is suitable for publication in GigaScience. A number of minor concerns, however, need to be addressed before publication:
1) The authors need to tone down in drawing conclusions related to genes, gene expression and behavior (e.g., food selection) and organ development. Although the differences in genes and gene expression may eventually be responsible for the observed biological differences in traits, a direct link of gene expression, as presented with differentially expressed genes, to traits is still far-fetched.
Answer 1
Thank you for your insightful comments. We acknowledge that our study has demonstrated correlations between gene expression and phenotypic traits, but these correlations do not necessarily establish a causal relationship. We have revised the manuscript to more accurately reflect the limitations of our study and have tempered the claims regarding the functional significance of the identified genes.

The details are as follows:
“Our findings provide preliminary evidence suggesting a potential rapid divergence in genetic diversity within the digestive organs of M. amblycephala and C. alburnus.” (page 17, lines 5-7)
“Among these genes, dpp2, ctrl, psb7, and ppce were identified as potential genes involved in peptidase activity, exhibiting higher expression in the digestive organs (liver and intestine) of C. alburnus compared to M. amblycephala (Fig. 3a).” (page 17, lines 13-16)
“These findings suggest that the observed genetic diversity may be related to adaptations in digestive enzyme secretion, reflecting potential dietary adjustments.” (page 17, lines 23-24)
“Gene flow and its potential impact on feeding habits” (page 18, line 2)
“Zbtb16a (linked to osteogenic differentiation (Felthaus et al. 2014)) exhibited the broadest expression pattern among all introgressed genes, being detected in five different tissues and organs (Fig. 4d). This suggests that zbtb16a may be a hotspot for introgression events between Cultrinae and Leuciscinae.” (page 18, lines 24-25, page 19, lines 1-2)

2) The readability of the paper needs to be improved. The paper, as it is written the way it stands now, is fairly technical. The authors are suggested to present the major findings in biologist's language such that its readability is enhanced.
Answer 2
We appreciate your kind comments. The Discussion section has been revised to incorporate a more biological viewpoint.
The major modifications are as follows:
“Among these, significant gene flow was detected between carnivorous C. alburnus (subfamily Cultrinae) and E. bambusa (subfamily Leuciscinae) (Z-score > 45.8, f4–ratio = 0.039, and p-value < 0.001).” (page 18, lines 4-6)
“Moreover, 84 categories (38.36%) for biological processes and 16 categories (66.67%) for molecular functions were shared between carnivores and herbivores (FDR < 0.05, Fig. 4c and Supplemental File 2: Figs. S9-S10).” (page 18, lines 19-22)
“Zbtb16a (linked to osteogenic differentiation (Felthaus et al. 2014)) exhibited the broadest expression pattern among all introgressed genes, being detected in five different tissues and organs (Fig. 4d). This suggests that zbtb16a may be a hotspot for introgression events between Cultrinae and Leuciscinae. ” (page 18, lines 24-25, page 19, lines 1-2)
“Chromosome-scale genomes were assembled for blunt snout bream (Megalobrama amblycephala, BSB) and topmouth culter (Culter alburnus, TC) using 204.8 Gb Hi-C data. The resulting assemblies exhibited scaffold N50 values of 42.91 Mb and 39.60 Mb, respectively (Table 1 and Supplemental File 1: Tables S1-S2).” (page 13, lines 1-4)

Responses to the comments of the reviewer 2:
Reviewer #2: This study aims to unravel the intricate relationship between hybridization events and the evolutionary origins of cyprinid fish. By assembling the high-quality genomes of eight cyprinid fish species and integrating publicly available genomic data, this study sheds light on the pivotal role of gene flow events in the evolution of cyprinid fish. Through comprehensive multi-tissue transcriptomic analyses, the study delves into the intricate interplay among reproductive isolation, gene flow dynamics, and the development of digestive organs. This is an intriguing study exploring the mutual influence between the origin of hybridization and dietary adaptation in cyprinid fishes.
Answer
We are grateful for the insightful comments. These suggestions have greatly enhanced the quality of our manuscript.

To enhance the research's value and readability, it is advisable to address some minor issues before publication:
1. The figure legends of both main and supplemental figures are generally uninformative and lack adequate detail. A few examples:
Scale of the heatmaps. Are these z-scores?
Several figures feature TC and BSB acronyms. While I understand each of these refers to either M. amblycephala or C. alburnus, I cannot find anywhere in the manuscript or supplemental material any information on which is which.

Answer 1
For Figure 1B, Scale of the heatmap is f-branch value. We have revised it in the Figure 1B.

We have added a description of the full Latin names corresponding to the acronyms used in the legends of Figures 3, S2, S7, S10, and S11.
“Figure 3: Diet divergences between M. amblycephala and C. alburnus. (a) The four genes relating to differential expression between M. amblycephala (BSB) and C. alburnus (TC) in both the intestine and liver (three biological replicates showed “_1”, “_2”, and “_3”).” (page 31, lines 20-23 in main text)
“Figure S2. The Hi-C interaction heatmap of 24 linkage groups in the genomes of Megalobrama amblycephala and Culter alburnus.” (page 2, lines 16-17 in Supplementary Figures)
“Figure S7. The DEGs (M. amblycephala vs. C. alburnus) associated with diet habit. a. The heatmap of the DEGs in the intestine. The hydrolyzing O-glycosyl compounds and peptidase activity in Molecular Function, as well as carbohydrate metabolic process and lipid catabolic process in Biological Process. TC: C. alburnus, BSB: M. amblycephala.” (page 4, lines 40-41 in Supplementary Figures)
“Figure S10. The distribution of enriched functional categories (FDR < 0.05) in Biological Process and Molecular Function for the introgressed genes. Carnivore: C. alburnus and Elopichthys bambusa, herbivore: M. amblycephala and Ctenopharyngodon idella.” (page 6, lines 53-55 in Supplementary Figures)
“Figure S11. Heatmap exhibiting the expression of introgressed genes in the carnivorous (C. alburnus and E. bambusa) and herbivorous (M. amblycephala and C. idella) fishes. TC: C. alburnus, BSB: M. amblycephala.” (page 6, lines 58 in Supplementary Figures)

2. Add a sentence in the legend describing the color scheme used in Figure 3.
Answer 2
In Figure 3A, we have implemented a new color scheme to differentiate between liver and intestine samples. Furthermore, we have added a scale bar with appropriate units to the heatmap to provide a quantitative measure of gene expression levels.

Figure 3: Diet divergences between M. amblycephala and C. alburnus
(A) The four genes relating to differential expression between M. amblycephala (BSB) and C. alburnus (TC) in both the intestine and liver (three biological replicates showed “_1”, “_2”, and “_3”). (B) Significant differences in the enzyme activity of lipase and trypsin for the comparison between M. amblycephala and C. alburnus.

3. Please unify Mya in the introduction and results sections (MYA). Pay attention to the writing as sometimes it is a full name and sometimes it is an abbreviation.
Answer 3
We appreciate your feedback. To ensure consistency, we have standardized the age terminology throughout the manuscript, using "million years ago" (MYA) in all relevant instances.

You can consider including a "List of Abbreviations" section at the beginning of the manuscript to list all abbreviations used and their full forms. This would improve readability for future readers.
Go through the entire manuscript one more time to ensure consistency in terminology, punctuation, and formatting.
Answer 4
To enhance clarity and readability, we have added a section of abbreviations at the end of the manuscript, providing the full terms for all abbreviations used. (page 23, lines 24-25, page 24, lines 1-3)

Responses to the comments of the reviewer 3:
Reviewer #3: Ren et al. present new cyprinid genome assemblies and RNA-seq expression data to evaluate hybridization occurrence across lineages in this ecologically diverse and species family.
Assembly Quality: The genome assemblies appear to be high quality. I have noted some confusion about methods and results which could be clarified.
Answer 1
Thanks for your comments on the genome assembly. In the revised manuscript, we have highlighted the revised methods and results in yellow.

The data was analyzed extensively, which I appreciate, but the manuscript is very long and the extensive analyses make the paper difficult to read and follow in detail. Honing down a tighter story in favor of fewer, but more relevant analyses would be helpful.
Answer 2
Your comments have significantly improved the clarity of our manuscript. We have made substantial revisions to the presentation of our analyses, particularly in the results section, which are now highlighted in yellow. These changes should enhance the reader's understanding of our findings.

Hybridization tests: forgive my ignorance on how these tests work, but I wonder if there is a possibility that significant departures from null for ABBA-BABA or f4 tests could instead be the result of which sex was sequenced for each species, e.g., if there is a bias introduced by some genome assemblies coming from male vs. female parents? I might have missed the SNP calling methods but this could affect the results - did these come from phased assemblies? Or mapping reads to collapsed haploid assemblies? For the polyploid species, this could also impact hybridization tests. How much of this signal of introgressive hybridization is due to long (or short) histories of artificial breeding (sometimes intentional) in these species?
Answer 3
As described in the hybridization tests on page 15, lines 13-17, while no natural hybrid populations of M. amblycephala and C. alburnus were found in overlapping habitats, we successfully produced fertile F1 hybrids through artificial breeding. Subsequent self-crossing of these F1 hybrids generated F2 generations, establishing multi-generational hybrid lines between the two species. These results indicate the absence of reproductive isolation between M. amblycephala and C. alburnus.
Genome assemblies were constructed using DNA from non-inbred individuals. The absence of sex chromosomes in these cyprinid fish reduced potential biases associated with sex-specific genomic regions. SNPs were identified using a modified version of Chen's method (Chen and Qiu 2019), aligning whole genomes to the zebrafish reference (page 9, lines 16-20). Our analysis focused solely on diploid genomes from non-inbred populations to avoid complications introduced by phased assemblies or inbreeding. It has come to our attention that previous studies have employed ABBA-BABA or f4 ratio tests to investigate gene flow without accounting for sex-specific effects (Flury et al. 2023).
While phylogenetic analyses were conducted for the all 24 species, ABBA-BABA tests were restricted to 17 non-polyploid cyprinid fish. The detail decryption is as follow:
“The conserved scores of the 17 non-polyploid species were estimated using the phastCons tool from the phast packages (Cooper et al. 2005). VCF files for each species were generated by aligning whole genomes to the zebrafish genome using Chen's methods (Chen and Qiu 2019). To investigate gene flow, we exclusively analyzed the 17 non-polyploid genomes from non-inbred populations using the ABBA-BABA test implemented in the Dsuite (0.4 r38) software (Malinsky et al. 2021) with the D-statistic method” (page 9, lines 15-20)
To further clarify our findings, we rephrased the results section as follows:
“To investigate this, we conducted gene flow analysis and observed pervasive introgression among the 17 non-polyploid cyprinid fishes (f4-ratio > 0.0006, Z-score > 3, and p-value < 0.05) (Fig. 1b).” (page 14, lines 19-21)
You raised an interesting question about the potential impact of artificial breeding on our observed introgression signals. While our samples were collected from wild populations, as detailed in the 'sample collection' section of the Methods, we cannot entirely rule out the possibility of escaped farmed individuals. Addressing this question definitively would require additional studies and a more complex experimental design. However, this is an intriguing area for future research.

Conclusion (pg 2, line 15-17): Why does gene flow drive speciation? The mechanism here is unclear, as pure introgression simply homogenizes existing variation (although more generally I recognize the *potential* creative role of hybridization). Diet can play a role in habitat preferences, but this only matters if those differences prevent reproduction (and persist at that time), but I do not see evidence of actual mechanistic drivers of reproductive isolation (the authors correctly acknowledge this), but the divergence of dietary enzymes is likely even in the absence of hybridization, particularly as copy numbers of these expand and contract (see amylase gene copy number variation in domestic mammals, for example). That variation is not related to hybridization, so the link is tenuous.
Answer 4
We appreciate your insightful comments on the role of gene flow in driving speciation. We agree that gene

flow is a complex phenomenon and its impact on speciation can vary among species. Previous studies have shown that gene flow can promote speciation in some cases, such as through ecological niche divergence or genomic conflict. However, in other cases, gene flow can hinder speciation, for example, by homogenizing existing variation.
In our study, we found frequent introgression among East Asian Cyprinidae, suggesting that gene flow may have a significant impact on the speciation of these species. However, we were unable to fully determine the specific mechanisms by which gene flow drives speciation in these species. Future studies may need to further explore potential mechanisms, such as ecological niche divergence, genomic conflict, or hybrid vigor.
We thank you for your comments and agree that differences in dietary enzymes may be an important factor in the speciation of East Asian Cyprinidae. However, our study did not directly demonstrate a causal relationship between dietary enzyme differences and speciation. Future research may need to further explore the link between dietary enzyme differences and speciation.

Minor comments:
1. Pg2, line 9: "conservative divergence in the testis" is unclear. I assume this means minimal expression differences?
Answer 1
Our comparative analysis of six tissues in M. amblycephala and C. alburnus indicated that, contrary to the reviewer's suggestion, the testis showed a relatively conserved pattern of evolutionary change. This conclusion was drawn from an assessment of multiple metrics, including gene expression divergence, rates of nonsynonymous to synonymous substitutions (Ka/Ks), and evolutionary conservation scores.
The detailed revisions are as follows:
“Based on a systematic analysis of multiple tissues, we found that the testis exhibited a conserved pattern of divergence between the herbivorous Megalobrama amblycephala and the carnivorous Culter alburnus, suggesting a potential link to incomplete reproductive isolation.” (page 2, lines 8-11)

2. Pg3, line 13 - the terms 'hybridization' and 'introgression' are conflated here - they are not synonymous.
Answer 2
Thank you for pointing out the error in our previous statement. We have revised the sentence to:
“Introgression plays a significant and frequent role in adaptive evolution” (page 3, line 13)

3. Pg3, line 14 - the Mallet citation refers to 10% of animal *species* being involved in hybridization, rather than 10% of wild animals (individuals).
Answer 3
We have revised this sentence according to the kind comment as follow:
"At least 10% of animal species are involved in hybridization, although most hybrid individuals have low viability or are sterile (Mallet 2005)" (page 3, lines 13-15)

4. Pg3, line 15 - The sentence is unclear. I think the authors are trying to say that intergeneric hybrids are rare in birds and mammals because of reproductive isolation, but this is tautological, unless the authors have a different definition of reproductive isolation.
Answer 4
We have revised this sentence according to the kind comment as follow:
“While reproductive isolation (RI) is frequently observed in intergeneric hybridization among birds and mammals, it is less common in cyprinid fish species, as evidenced by the documented instances of disrupted isolation” (page 3, lines 15-17)

5. Pg5, line 7 - I don't quite understand. The three males are hybrid offspring of the two parental species? Which was the maternal vs paternal species?
Answer 5
To clarify, we have rephrased the sentence as follows:
“Three sexually mature male Culter alburnus and Megalobrama amblycephala, raised in identical controlled conditions for 24 months post-hatching, were bred at the Engineering Center of Polyploid Fish Breeding, National Education Ministry, Changsha, Hunan, China. The broodstock were sourced from the Yangtze River (30°25'56" N, 114°50'32" E).” (page 5, lines 6-9)

Pg6, line 8 - this says nanopore sequencing, but later it says PacBio was conducted. Above (line 3) it says Ampure beads, manufacturer PacBio, but this also is incorrect (Beckman-Coulter, Inc.).
Answer 6
There was an error in the methodology regarding the sequencing platform used for C. alburnus and M. amblycephala. While we employed Nanopore sequencing for these two species, the other six fish species (C. idella, C. molitorella, P. parva, X. davidi, G. rarus, and E. bambusa) were sequenced using PacBio Sequel II. Specifically, the statement regarding the use of AMPure PB beads for purifying the genomic DNA of C. alburnus and M. amblycephala was incorrect. We have corrected this error in the methods section as follow:
"After quality checking, the genomic DNA of C. alburnus and M. amblycephala was randomly sheared using Megaruptor (Diagenode, Denville, NJ, USA). DNA was size-selected using a SPRI bead protocol. The purity of the extracted DNA was determined using a Nanodrop spectrophotometer (Thermo Fisher Scientific). All procedures were carried out at room temperature. Large DNA fragments were separated using BluePippin DNA Size Selection System. DNA damage repair and end-repair were performed. Barcoded overhang hairpin adapters were ligated to the fragment ends. The connection reaction was performed using Ligation Sequencing Kit (Oxford Nanopore, SQK-LSK108). A constructed DNA library was quantified using Qubit. Lastly, sequencing was performed using Nanopore Sequencing." (page 5, line 25, page 6, lines 1-8)

7. Was 'ligation sequencing kit' (line 7) Oxford Nanopore Technologies, Inc? Or another kit? Pg 12, lists Oxford Nanopore, but no mention of this is in the methods, including which platform.
Answer 7
Thank you for your kind comments! We have incorporated the information about the 'ligation sequencing kit' and revised the sentence as follows:
"The connection reaction was performed using Ligation Sequencing Kit (Oxford Nanopore, SQK-LSK108)." (page 6, lines 6-7)

8. Pg6, line 25 - No citation given for the 'standard protocol' for DNBSEQ-T7 - could the authors provide one?
Answer 8
Thank you for your valuable comment! We have added a reference to the literature: "Comparison between MGI and Illumina sequencing platforms for whole genome sequencing." (page 27, lines 20-21)

9. Pg7, line9 - some of these species already have high quality reference genomes - what is the rationale for additional sequencing? What sex were the individuals that were sequenced for genome assembly? Were haplotigs purged from draft assemblies or evaluated? Contamination from microbiome?
Answer 9
Prior to our study, high-quality genome assemblies generated using PacBio HiFi reads were absent for these six species. These high-quality assemblies are essential for conducting comprehensive comparative genomic analyses. Currently, to our knowledge, only C. idella and G. rarus among these six species have publicly high-quality genome assemblies produced using PacBio HiFi reads.
Due to the immature stage of some collected fish, we were unable to collect sex information for these species.
The assembled genome is not a haplotype genome. Comparisons of our assembly with published genomes of the same or closely related species indicate that its size is consistent with the expected genome size for this species, rather than representing a haploid genome.
We have rigorously assessed our assembly for microbial contamination through both annotation analyses and National Genomics Data Center (NGDC) submission checks. These analyses have confirmed the absence of significant microbial contamination in our data.

10. Pg8, line 4 - its unclear why the reference genomes of e.g., Ctenopharyngodon idella, were not used for genome annotation, given these are the species being assembled.
Answer 10
Yes, there is a PacBio Sequel-based genome assembly of Ctenopharyngodon idella available online, released in February 2023 (https://ftp.ncbi.nlm.nih.gov/genomes/all/GCF/019/924/925/GCF_019924925.1_HZGC01/). However, we had already completed our analysis by that time. Therefore, when we conducted genome annotation, we did not use the annotation file from this specific genome version.

11. Pg 12, line 20 - how were these data split across the various species and sequencing technologies?
Answer 11
We have presented a detailed illustration of this in Supplementary Table 1.
Moreover, we have elaborated on this point in the revised manuscript as follows:
"A total of eight species of cyprinid fishes from East Asia were sequenced using PacBio HiFi or Oxford Nanopore technology, resulting in over 602.21 Gb of raw data (Supplemental File 1: Tables S1)." (page 12, lines 23-25)

12. Pg 14, line 19-20 - what about the various genome papers that have been published and included genome scale phylogenomics? Are the results concordant with previous findings? (Molecular vs morphology discordance is expected).
Answer 12
By integrating our phylogenomic analysis with previous genomic studies, we have estimated divergence times for major lineages within the Cyprinidae family.
Our analysis suggested that the common ancestor of extant cyprinids diverged between 81.9 and 100.0 MYA (Fig. 1a, page 14, lines 9-11), which is consistent with the estimated divergence time of 81.9-100.1 MYA for the most recent common ancestor of Nemacheilidae and Cyprinidae in a previous study (Xu et al. 2023).
Furthermore, we calculated that the ancestor of Danio rerio (subfamily: Danioninae) diverged early in the evolution of extant cyprinids, while the ancestor of Cirrhinus molitorella and Labeo rohita (subfamily: Labeoninae) diverged between 36.5 and 53.6 MYA (Fig. 1a and Supplemental File 1: Table S9, page 14 lines 11-14). These divergence times are comparable to the estimates of 40.4–48.6 MYA (Xu et al. 2023), ~37 MYa (Feng et al. 2023), and ~60 Mya (Xu et al. 2019) reported in previous studies.
Additionally, we calculated that the divergence time of Leuciscinae (C. idella and E. bambusa), Hypophthalmichthyinae, Xenocypridinae, and Cultrinae is about 17 MYA. (Fig. 1a, page 14, lines 7-8). These results are generally consistent with previous estimates of 11.6–12.7 MYA (Xu et al. 2023), 15.8–14.5 Ma (Feng et al. 2023), and 12.69 Ma (Wang et al. 2024).
Finally, our analysis indicates that Cyprininae diverged from other subfamilies approximately 20 MYA (Fig. 1a), which is comparable to the estimate of 25.18 MYA (95% CI: 20.75–28.03 Ma) reported in a previous study (Li and Guo 2020).

13. Table 1 - it would be helpful to have duplicate and single copy (and missing and fragmented) busco information here to evaluate possible ploidy issues.
Answer 13
The BUSCO analysis results for the eight assembled genomes are summarized in Supplementary Table 3.

14. FigS1 - Branch support is needed here to evaluate phylogenetic support.
Answer 14
As shown in Figure S1, the bootstrap support values for all branches in the phylogenetic trees were 100%. We have included the above information in the legend of Figure S1.

Reference:
Chen L, Qiu Q. 2019. Large-scale ruminant genome sequencing provides insights into their evolution and distinct traits. 364(6446).
Cooper GM, Stone EA, Asimenos G, Green ED, Batzoglou S, Sidow A. 2005. Distribution and intensity of constraint in mammalian genomic sequence. Genome research 15(7): 901-913.
Felthaus O, Gosau M, Morsczeck C. 2014. ZBTB16 induces osteogenic differentiation marker genes in dental follicle cells independent from RUNX2. Journal of periodontology 85(5): e144-151.
Feng C, Wang K, Xu W, Yang L, Wanghe K, Sun N, Wu B, Wu F, Yang L, Qiu Q et al. 2023. Monsoon boosted radiation of the endemic East Asian carps. Science China Life sciences 66(3): 563-578.
Flury JM, Meusemann K, Martin S, Hilgers L, Spanke T, Böhne A, Herder F, Mokodongan DF, Altmüller J, Wowor D et al. 2023. Potential Contribution of Ancient Introgression to the Evolution of a Derived Reproductive Strategy in Ricefishes. Genome biology and evolution 15(8).
Li X, Guo B. 2020. Substantially adaptive potential in polyploid cyprinid fishes: evidence from biogeographic, phylogenetic and genomic studies. Proceedings Biological sciences / The Royal Society 287(1920): 20193008.

Malinsky M, Matschiner M, Svardal H. 2021. Dsuite - Fast D-statistics and related admixture evidence from VCF files. Mol Ecol Resour 21(2): 584-595.
Mallet J. 2005. Hybridization as an invasion of the genome. Trends Ecol Evol 20(5): 229-237.
Wang C, Yang L, Lu Y, Fang C, Gan X, Chen Y, He S. 2024. Genomic features for adaptation and evolutionary dynamics of four major Asian domestic carps. Science China Life sciences 67(6): 1308-1310.
Xu M-R-X, Liao Z-Y, Brock JR, Du K, Li G-Y, Chen Z-Q, Wang Y-H, Gao Z-N, Agarwal G, Wei KHC et al. 2023. Maternal dominance contributes to subgenome differentiation in allopolyploid fishes. Nature communications 14(1): 8357.
Xu P, Xu J, Liu GJ, Chen L, Zhou ZX, Peng WZ, Jiang YL, Zhao ZX, Jia ZY, Sun YH et al. 2019. The allotetraploid origin and asymmetrical genome evolution of the common carp Cyprinus carpio. Nature communications 10.
