# Supplementary material for: Genomes reveal pervasive distant hybridization in nature among cyprinid fishes
Source: Gigascience. 2025 Jan 30;14:giae117. doi: 10.1093/gigascience/giae117 (PMC11779505; doi:10.1093/gigascience/giae117)
Supplement: giae117_Reviewer_1_Report_Original_Submission — Zhanjiang Liu -- 8/7/2024 Reviewed [file giae117_reviewer_1_report_original_submission.pdf]

Reviewer Report

Title: Genomes reveal pervasive distant hybridization in nature among cyprinid fishes

Version: Original Submission **Date:** 8/7/2024

Reviewer name: Zhanjiang Liu

Reviewer Comments to Author:

This paper reported their work of genome sequencing and assembly of eight cyprinid fishes and phylogenetic analyses using genomic sequences from a couple of dozens of species. Thedy found widespread allele sharing across species boundaries within seven subfamilies of cyprinid fishes. The major findings are incomplete reproductive isolationand frequent gene flow among some of these species. In spite of being expected, this research provided empirical evidence, which is significant. As such, it is suitable for publication in GigaScience. A number of minor concerns, however, need to be addressed before publication:1) The authors need to tone down in drawing conclusions related to genes, gene expression and behavior (e.g., food selection) and organ development. Although the differences in genes and gene expression may eventually be responsible for the observed biological differences in traits, a direct link of gene expression, as presented with differentially expressed genes, to traits is still far-fetched.2) The readability of the paper needs to be improved. The paper, as it is written the way it stands now, is fairly technical. The authors are suggested to present the major findings in biologist's language such that its readability is enhanced.

Level of Interest

Please indicate how interesting you found the manuscript: Choose an item.

Quality of Written English

Please indicate the quality of language in the manuscript: Choose an item.

Declaration of Competing Interests

Please complete a declaration of competing interests, considering the following questions:

- Have you in the past five years received reimbursements, fees, funding, or salary from an organisation that may in any way gain or lose financially from the publication of this manuscript, either now or in the future?
- Do you hold any stocks or shares in an organisation that may in any way gain or lose financially from the publication of this manuscript, either now or in the future?
- Do you hold or are you currently applying for any patents relating to the content of the manuscript?

- Have you received reimbursements, fees, funding, or salary from an organization that holds or has applied for patents relating to the content of the manuscript?
- Do you have any other financial competing interests?
- Do you have any non-financial competing interests in relation to this paper?

If you can answer no to all of the above, write 'I declare that I have no competing interests' below. If your reply is yes to any, please give details below.

I declare that I have no competing interests

I agree to the open peer review policy of the journal. I understand that my name will be included on my report to the authors and, if the manuscript is accepted for publication, my named report including any attachments I upload will be posted on the website along with the authors' responses. I agree for my report to be made available under an Open Access Creative Commons CC-BY license (http://creativecommons.org/licenses/by/4.0/). I understand that any comments which I do not wish to be included in my named report can be included as confidential comments to the editors, which will not be published.

Choose an item.

To further support our reviewers, we have joined with Publons, where you can gain additional credit to further highlight your hard work (see: https://publons.com/journal/530/gigascience). On publication of this paper, your review will be automatically added to Publons, you can then choose whether or not to claim your Publons credit. I understand this statement.

Yes Choose an item.
