# Supplementary material for: Genomes reveal pervasive distant hybridization in nature among cyprinid fishes
Source: Gigascience. 2025 Jan 30;14:giae117. doi: 10.1093/gigascience/giae117 (PMC11779505; doi:10.1093/gigascience/giae117)
Supplement: giae117_Reviewer_2_Report_Original_Submission — Liandong Yang -- 9/3/2024 Reviewed [file giae117_reviewer_2_report_original_submission.pdf]

Reviewer Report

Title: Genomes reveal pervasive distant hybridization in nature among cyprinid fishes

Version: Original Submission **Date:** 9/3/2024

Reviewer name: Liandong Yang

Reviewer Comments to Author:

This study aims to unravel the intricate relationship between hybridization events and the evolutionary origins of cyprinid fish. By assembling the high-quality genomes of eight cyprinid fish species and integrating publicly available genomic data, this study sheds light on the pivotal role of gene flow events in the evolution of cyprinid fish. Through comprehensive multi-tissue transcriptomic analyses, the study delves into the intricate interplay among reproductive isolation, gene flow dynamics, and the development of digestive organs. This is an intriguing study exploring the mutual influence between the origin of hybridization and dietary adaptation in cyprinid fishes.To enhance the research's value and readability, it is advisable to address some minor issues before publication:1. The figure legends of both main and supplemental figures are generally uninformative and lack adequate detail. A few examples:Scale of the heatmaps. Are these z-scores?Several figures feature TC and BSB acronyms. While I understand each of these refers to either M. amblycephala or C. alburnus, I cannot find anywhere in the manuscript or supplemental material any information on which is which.2. Add a sentence in the legend describing the color scheme used in Figure 3.3. Please unify Mya in the introduction and results sections (MYA). Pay attention to the writing as sometimes it is a full name and sometimes it is an abbreviation.You can consider including a "List of Abbreviations" section at the beginning of the manuscript to list all abbreviations used and their full forms. This would improve readability for future readers.Go through the entire manuscript one more time to ensure consistency in terminology, punctuation, and formatting.

Level of Interest

Please indicate how interesting you found the manuscript: Choose an item.

Quality of Written English

Please indicate the quality of language in the manuscript: Choose an item.

Declaration of Competing Interests

Please complete a declaration of competing interests, considering the following questions:

- Have you in the past five years received reimbursements, fees, funding, or salary from an organisation that may in any way gain or lose financially from the publication of this manuscript, either now or in the future?

- Do you hold any stocks or shares in an organisation that may in any way gain or lose financially from the publication of this manuscript, either now or in the future?
- Do you hold or are you currently applying for any patents relating to the content of the manuscript?
- Have you received reimbursements, fees, funding, or salary from an organization that holds or has applied for patents relating to the content of the manuscript?
- Do you have any other financial competing interests?
- Do you have any non-financial competing interests in relation to this paper?

If you can answer no to all of the above, write 'I declare that I have no competing interests' below. If your reply is yes to any, please give details below.

I declare that I have no competing interests

I agree to the open peer review policy of the journal. I understand that my name will be included on my report to the authors and, if the manuscript is accepted for publication, my named report including any attachments I upload will be posted on the website along with the authors' responses. I agree for my report to be made available under an Open Access Creative Commons CC-BY license (http://creativecommons.org/licenses/by/4.0/). I understand that any comments which I do not wish to be included in my named report can be included as confidential comments to the editors, which will not be published.

Choose an item.

To further support our reviewers, we have joined with Publons, where you can gain additional credit to further highlight your hard work (see: https://publons.com/journal/530/gigascience). On publication of this paper, your review will be automatically added to Publons, you can then choose whether or not to claim your Publons credit. I understand this statement.

Yes Choose an item.
