# Supplementary material for: Genomes reveal pervasive distant hybridization in nature among cyprinid fishes
Source: Gigascience. 2025 Jan 30;14:giae117. doi: 10.1093/gigascience/giae117 (PMC11779505; doi:10.1093/gigascience/giae117)
Supplement: giae117_Reviewer_3_Report_Original_Submission — Trevor Krabbenhoft -- 9/30/2024 Reviewed [file giae117_reviewer_3_report_original_submission.pdf]

Reviewer Report

Title: Genomes reveal pervasive distant hybridization in nature among cyprinid fishes

Version: Original Submission **Date:** 9/30/2024

Reviewer name: Trevor Krabbenhoft

Reviewer Comments to Author:

Ren et al. present new cyprinid genome assemblies and RNA-seq expression data to evaluate hybridization occurrence across lineages in this ecologically diverse and speciose family.Assembly Quality: The genome assemblies appear to be high quality. I have noted some confusion about methods and results which could be clarified.The data was analyzed extensively, which I appreciate, but the manuscript is very long and the extensive analyses make the paper difficult to read and follow in detail. Honing down a tighter story in favor of fewer, but more relevant analyses would be helpful.Hybridization tests: forgive my ignorance on how these tests work, but I wonder if there is a possibility that significant departures from null for ABBA-BABA or f4 tests could instead be the result of which sex was sequenced for each species, e.g., if there is a bias introduced by some genome assemblies coming from male vs female parents? I might have missed the SNP calling methods but this could affect the results - did these come from phased assemblies? Or mapping reads to collapsed haploid assemblies? For the polyploid species, this could also impact hybridization tests. How much of this signal of introgressive hybridization is due to long (or short) histories of artificial breeding (sometimes intentional) in these species?Conclusion (pg 2, line 15-17): Why does gene flow drive speciation? The mechanism here is unclear, as pure introgression simply homogenizes existing variation (although more generally I recognize the *potential* creative role of hybridization). Diet can play a role in habitat preferences, but this only matters if those differences prevent reproduction (and persist at that time), but I do not see evidence of actual mechanistic drivers of reproductive isolation (the authors correctly acknowledge this), but the divergence of dietary enzymes is likely even in the absence of hybridization, particularly as copy numbers of these expand and contract (see amylase gene copy number variation in domestic mammals, for example). That variation is not related to hybridization, so the link is tenuous.Minor comments:Pg2, line 9: "conservative divergence in the testis" is unclear. I assume this means minimal expression differences?Pg3, line 13 - the terms 'hybridization' and 'introgression' are conflated here - they are not synonymous.Pg3, line 14 - the Mallet citation refers to 10% of animal *species* being involved in hybridization, rather than 10% of wild animals (individuals).Pg3, line 15 - The sentence is unclear. I think the authors are trying to say that intergeneric hybrids are rare in birds and mammals because of reproductive isolation, but this is tautological, unless the authors have a different definition of reproductive isolation.Pg5, line 7 - I don't quite understand. The three males are hybrid offspring of the two parental species? Which was the maternal vs paternal species?Pg6, line 8 - this says nanopore sequencing, but later it says PacBio was conducted. Above (line 3) it says Ampure beads, manufacturer PacBio, but this also is incorrect (Beckman-Coulter, Inc.).Was 'ligation sequencing kit' (line 7) Oxford Nanopore Technologies, Inc? Or another kit? Pg 12, lists Oxford Nanopore, but no mention of this is in the methods, including which platform.Pg6, line 25 - No citation given for the 'standard

protocol' for DNBSEQ-T7 - could the authors provide one?Pg7, line9 - some of these species already have high quality reference genomes - what is the rationale for additional sequencing? What sex were the individuals that were sequenced for genome assembly? Were haplotigs purged from draft assemblies or evaluated? Contamination from microbiome?Pg8, line 4 - its unclear why the reference genomes of e.g., Ctenopharyngodon idella, were not used for genome annotation, given these are the species being assembled.Pg 12, line 20 - how were these data split across the various species and sequencing technologies?Pg 14, line 19-20 - what about the various genome papers that have been published and included genome scale phylogenomics? Are the results concordant with previous findings? (Molecular vs morphology discordance is expected).Table 1 - it would be helpful to have duplicate and single copy (and missing and fragmented) busco information here to evaluate possible ploidy issues.FigS1 - Branch support is needed here to evaluate phylogenetic support.

Level of Interest

Please indicate how interesting you found the manuscript: Choose an item.

Quality of Written English

Please indicate the quality of language in the manuscript: Choose an item.

Declaration of Competing Interests

Please complete a declaration of competing interests, considering the following questions:

- Have you in the past five years received reimbursements, fees, funding, or salary from an organisation that may in any way gain or lose financially from the publication of this manuscript, either now or in the future?
- Do you hold any stocks or shares in an organisation that may in any way gain or lose financially from the publication of this manuscript, either now or in the future?
- Do you hold or are you currently applying for any patents relating to the content of the manuscript?
- Have you received reimbursements, fees, funding, or salary from an organization that holds or has applied for patents relating to the content of the manuscript?
- Do you have any other financial competing interests?
- Do you have any non-financial competing interests in relation to this paper?

If you can answer no to all of the above, write 'I declare that I have no competing interests' below. If your reply is yes to any, please give details below.

no

I agree to the open peer review policy of the journal. I understand that my name will be included on my report to the authors and, if the manuscript is accepted for publication, my named report including any attachments I upload will be posted on the website along with the authors' responses. I agree for my report to be made available under an Open Access Creative Commons CC-BY license (http://creativecommons.org/licenses/by/4.0/). I understand that any comments which I do not wish to be included in my named report can be included as confidential comments to the editors, which will not be published.

Choose an item.

To further support our reviewers, we have joined with Publons, where you can gain additional credit to further highlight your hard work (see: https://publons.com/journal/530/gigascience). On publication of this paper, your review will be automatically added to Publons, you can then choose whether or not to claim your Publons credit. I understand this statement.

Yes Choose an item.
